# The Use of Innovative Two-Component Cluster Analysis and Serodiagnostic Cut-Off Methods to Estimate Prevalence of Pertussis Reinfections

**DOI:** 10.1371/journal.pone.0148507

**Published:** 2016-02-05

**Authors:** Inonge van Twillert, Axel A. Bonačić Marinović, Jacqueline A. M. van Gaans-van den Brink, Betsy Kuipers, Guy A. M. Berbers, Nicoline A. T. van der Maas, Theo J. M. Verheij, Florens G. A. Versteegh, Peter F. M. Teunis, Cécile A. C. M. van Els

**Affiliations:** 1 Centre for Infectious Disease Control, National Institute for Public Health and the Environment (RIVM), Bilthoven, the Netherlands; 2 Julius Center Health Sciences and Primary Care, University Medical Center Utrecht, Utrecht, the Netherlands; 3 Department of Pediatrics, Groene Hart Ziekenhuis, Gouda, the Netherlands; 4 Ghent University Hospital, department of pediatrics, Ghent, Belgium; 5 RSPH, Emory University, Atlanta, United States of America; Melbourne School of Population Health, AUSTRALIA

## Abstract

*Bordetella pertussis* circulates even in highly vaccinated countries affecting all age groups. Insight into the scale of concealed reinfections is important as they may contribute to transmission. We therefore investigated whether current single-point serodiagnostic methods are suitable to estimate the prevalence of pertussis reinfection. Two methods based on IgG-Ptx plasma levels alone were used to evaluate the proportion of renewed seroconversions in the past year in a cohort of *retrospective* pertussis cases ≥ 24 months after a proven earlier symptomatic infection. A Dutch population database was used as a baseline. Applying a classical 62.5 IU/ml IgG-Ptx cut-off, we calculated a seroprevalence of 15% in *retrospective cases*, higher than the 10% observed in the population baseline. However, this method could not discriminate between renewed seroconversion and waning of previously infection-enhanced IgG-Ptx levels. Two-component cluster analysis of the IgG-Ptx datasets of both pertussis cases and the general population revealed a continuum of intermediate IgG-Ptx levels, preventing the establishment of a positive population and the comparison of prevalence by this alternative method. Next, we investigated the complementary serodiagnostic value of IgA-Ptx levels. When modelling datasets including both *convalescent* and *retrospective cases* we obtained new cut-offs for both IgG-Ptx and IgA-Ptx that were optimized to evaluate renewed seroconversions in the ex-cases target population. Combining these cut-offs two-dimensionally, we calculated 8.0% reinfections in *retrospective cases*, being below the baseline seroprevalence. Our study for the first time revealed the shortcomings of using only IgG-Ptx data in conventional serodiagnostic methods to determine pertussis reinfections. Improved results can be obtained with two-dimensional serodiagnostic profiling. The proportion of reinfections thus established suggests a relatively increased period of protection to renewed infection after clinical pertussis.

## Introduction

*Bordetella pertussis* causes the respiratory infectious disease ‘whooping cough’ (pertussis) that has re-emerged as a public health issue since the 1990’s, affecting all age groups [1–3]. Pathogen adaptation [4, 5] and waning immunity after both vaccination and natural infection [6–8] are considered important causes. At present diagnosis is achieved by bacterial culture, polymerase chain reaction (PCR) and/or a serological test. The latter is the favoured method when diagnosis is performed > 3 weeks after onset [9]. Pertussis serodiagnosis is based on determining immunoglobulin G (IgG) antibodies in serum or plasma against pertussis toxin (Ptx), the only antigen specific for *B*. *pertussis*. Seroconversion of IgG-Ptx to higher levels is used as a biomarker for recent infection, elevations being temporary due to subsequent decay [10, 11]. While formerly pertussis serodiagnosis relied on the evaluation of a fold-increase in IgG-Ptx levels in paired sera taken a few weeks apart, presently absolute single-point IgG-Ptx values are often evaluated against a predefined cut-off. In an EU Pertstrain group comparison study, a single cut-off with optimal sensitivity and specificity was determined to be in the range 60–75 IU/ml [12]. An additional approach to single data point serodiagnostics are modelling based procedures that fit distribution mixtures and can refine the choice of diagnostic cut-offs. As recently reported, a dataset of fitted positive and negative clusters of IgG-Ptx from sera routinely submitted after disease onset from patients clinically suspected of pertussis, could indicate the proportion of true seroconversions [13]. Post-infection enhancement of pertussis specific immune mechanisms, such as levels of circulating antibodies, reduces the risk of subsequent infection, yet acquired protection is not lifelong. Estimates for the duration of protection vary from 4–20 years [6, 14, 15], depending on surveillance settings, pertussis circulation, case definitions, and vaccinations.

Because pertussis is endemic, repeat exposures and infections will occur [16], however at an unknown and likely underestimated rate, due to a- or mildly symptomatic cases [17]. Gaining insight into the scale of such concealed reinfections is important, since they may contribute to transmission when presenting with nonspecific symptoms like a persistent cough [18, 19]. Also, comparison of the estimated frequency of concealed reinfections in historic cases with the proportion of concealed infections in the general population may generate relevant information in the current debate regarding the duration of pertussis protective immunity after a previous (symptomatic) infection [20]. Not known thus far is whether current single-point serodiagnostic methods can be used to establish rates of reinfection in previously proven symptomatic cases, which is challenging because of residual antibody decay. Here we addressed this question by using various methodologies, taking advantage of a unique sample collection of a large Dutch cohort of clinically symptomatic, confirmed (ex) pertussis cases, from the observational clinical study SKI [21, 22]. Plasma samples were taken at a known, early or late time point after standard laboratory diagnostics, and were in-house serologically tested using an innovative widely accepted multiplex-based immunoassay (MIA) [23, 24]. We asked whether (concealed) reinfections could be serodiagnosed in *retrospective cases* sampled >24 months after their proven symptomatic infection, based on single point IgG-Ptx values, either employing a predefined cut-off of 62.5 IU/ml for likelihood of infection in the previous year [10, 23, 25, 26] or using mathematical modelling for seroclassification [13]. Population data from the national PIENTER-2 serosurvey were applied as a reference. Furthermore, we explored whether elevated plasma levels of Ptx specific immunoglobulin A (IgA-Ptx) with diagnostic potential [27–29] but a shorter half-life than IgG [30, 31], could improve methods to estimate the proportion of reinfections.

## Subjects and Methods

### Ethics Statement

This study was conducted according to the principles expressed in the Declaration of Helsinki. Participants were included from two studies. One study was a cross-sectional observational study in Dutch symptomatic pertussis cases and household contacts (Specifieke Kinkhoest Immuniteit, SKI) approved by the accredited Review Board Stichting Therapeutische Evaluatie Geneesmiddelen (STEG), Almere, and further managed by the Medical Ethics Testing Committee, University Medical Centre Utrecht, (METC UMC Utrecht) (CCMO nr: NL16334.040.07). The other study was a cross-sectional population-based serosurveillance study in the Netherlands (PIENTER-2) approved by the Review Board Stichting Therapeutische Evaluatie Geneesmiddelen (STEG), Almere (clinical trial number: ISRCTN 20164309). All participants provided written informed consent for the collection of blood samples, the usage of a completed questionnaire, and the immunological analysis of samples. Informed written consent for minor participants was provided by both parents or guardians.

### Study populations

**Fig 1:**
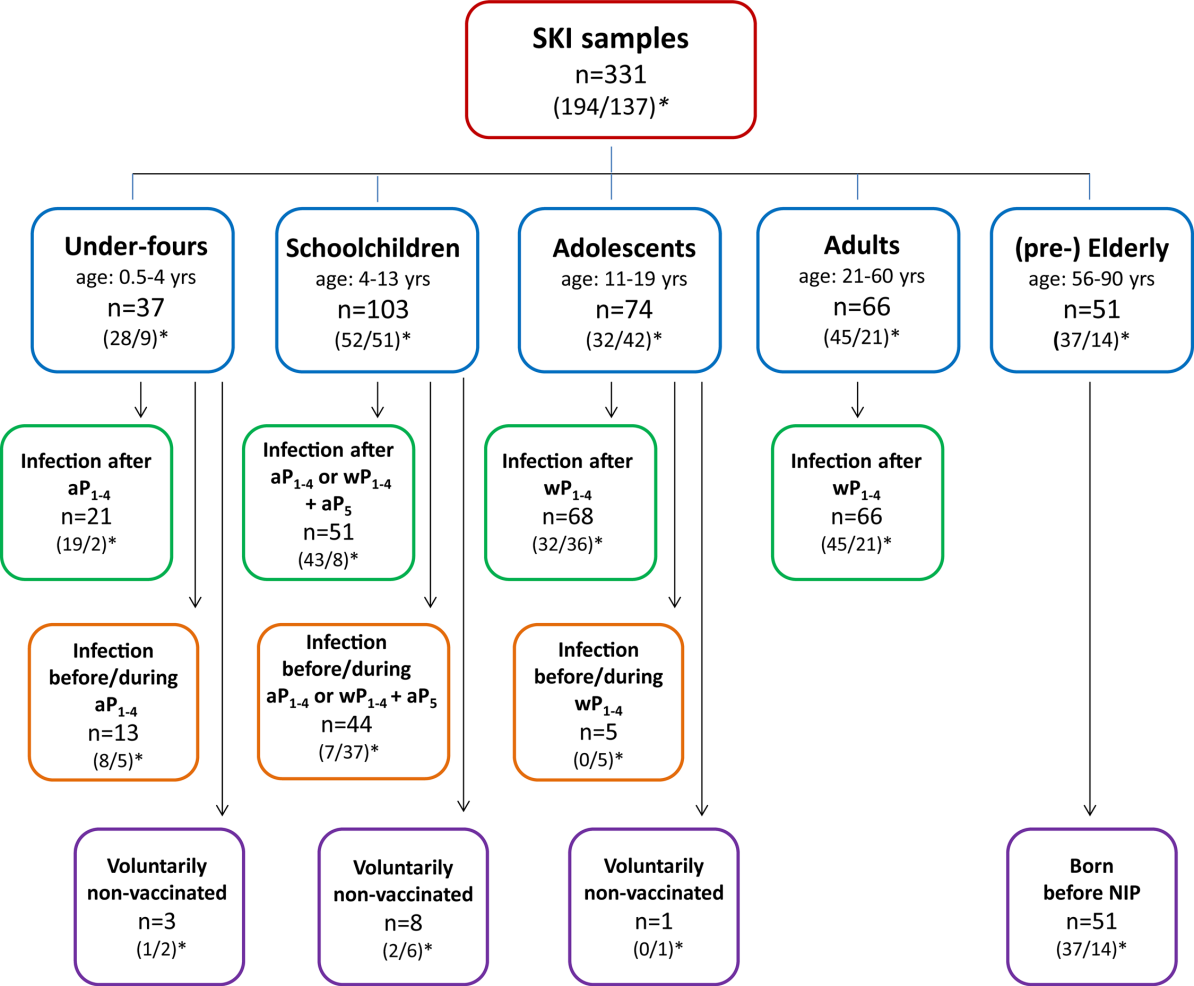
Flowchart of SKI samples stratified according to age and vaccination status. Fourtyfour percent of all SKI samples were from male (ex-)cases. (1,2)*: first number: number of cases having τ <24 months; second number: number of cases having τ ≥ 24 months. aP_1-4_: acellular pertussis vaccination doses 1–4; aP_5_: acellular pertussis preschool booster dose 5; wP_1-4_: whole cell pertussis vaccination doses 1–4. According to the Dutch National Immunization Program (NIP) pertussis vaccine primary doses 1–4 are given at 2,3,4, and 11 months of age, and the preschool booster dose 5 is given at 4 yrs of age.

Participants from the SKI study consisted of 292 (ex-) pertussis cases, who had presented themselves with clinical symptoms to their physicians and whose pertussis infections were confirmed by standardized diagnostic PCR-, culture- or serology-based assays in accredited laboratories. SKI cases represented all ages, and donated blood at a known time interval after diagnosis. All SKI blood samples were collected between 2008 and 2012. Of one cohort of cases (n = 194) blood was drawn within 24 months after the date of diagnosis, defined as “*convalescent cases”*. Within this cohort a subgroup of cases whose date of diagnosis was < 2 months before blood withdrawal was defined as “*acute cases*” (n = 145). Of the other cohort of cases (n = 98) blood was drawn ≥ 24 months after confirmed symptomatic pertussis. These samples, together with second blood samples obtained from 39 *acute case*s ≥ 24 months after their date of diagnosis, represented the “*retrospective cases*” (n = 137). As illustrated in Fig 1, the total of 331 samples represented five categories of participants, under-fours, schoolchildren, adolescents, adults and (pre-) elderly, based on age and vaccination status. All SKI samples were given a parameter *time after exposure* (τ, in months), indicating the time passed between the date of the pertussis diagnosis or of a last pertussis vaccination (if applicable), whichever was the more recent, and the date of blood sampling. As indicated in Fig 1, for 269 blood samples, the date of diagnosis was the last known date of exposure to pertussis antigens (green and purple framed boxes); for 62 samples, a pertussis vaccination of participants had taken place between their clinical pertussis episode and blood withdrawal (orange framed boxes). The PIENTER-2 study cohort consisted of 5740 subjects, randomly selected from the general Dutch population, excluding oversampled subcohorts from the original database [32]. PIENTER-2 blood samples were obtained in 2006/2007. Pertussis epidemic years were contained in the time frames of both PIENTER-2, i.e. 2007, and the SKI study, i.e. 2008, 2011 and 2012, respectively, with on average 6050 yearly cases in the PIENTER-2 time frame versus 7873 yearly pertussis notifications in the SKI time frame.

### Blood sampling

For the SKI study venous blood samples were collected using vacutainer cell preparation (CPT) tubes. Peripheral blood mononuclear cells (PBMC) and plasma were isolated using standard procedures. For the PIENTER-2 study venous blood samples were collected in serum tubes. Serum samples were isolated using standard procedures. Plasma and serum samples were stored at -80°C until testing.

### Serological assay

IgG-Ptx in SKI plasma samples was measured applying an in-house multiplex immunoassay described earlier [21, 24], using Goat anti-human IgG, R-PE (Jackson ImmunoResearch, USA) as reporter antibody. IgA-Ptx levels were measured likewise, using Goat anti-human-IgA-PE (Southern Biotech, USA) as reporter antibody, with the exception that serum samples were prepared in the dilutions 1/50 and 1/500. Beads were coated with Ptx protein (Kaketsuken, Japan) and the International WHO pertussis standard (06/140) was used as reference, therefore units are given in IU/ml. In our lab setting, these units are equivalent to the previously used ELISA units/ml (EU/ml) derived from the FDA human reference preparations lot 3, 4 and lot 5 [12]. IgG-Ptx in PIENTER-2 serum samples was previously measured with the multiplex immunoassay and expressed in EU/ml, as the used in-house reference was calibrated against the FDA human reference lot 3 [23]. Our IgG-Ptx (SKI and PIENTER-2 samples) and IgA-Ptx (SKI samples) data can be found in the supporting file: S1 table.

### Two-component cluster analyses

Serological two-component cluster analysis (also known as binary mixed modelling) assumes that the population sample can be described as one distribution of seropositive concentrations (the sero-converted component) and one of seronegative concentrations (the baseline component). The logarithms of seronegative and seropositive concentrations are assumed to be normally distributed with averages μ_0_ and μ_1_, respectively, and standard deviations σ_0_ and σ_1_. The mixed probability density function then becomes p*Norm(μ_1_, σ_1_) + (1-p)*Norm(μ_0_, σ_0_), where p is the prevalence. The parameters μ_1_, μ_0_, σ_1_ and σ_0_ were estimated for IgG-Ptx and for IgA-Ptx by means of the maximum likelihood method using combined data from all cohorts from the SKI study. Given their prevalence is assumed close to 100%, the *convalescent* and *acute* cohorts were included to feed the seropositive concentrations components for IgG-Ptx and IgA-Ptx, respectively. Using the estimated μ_0_, σ_0_, μ_1_, and σ_1_ parameters for pertussis specific IgG-Ptx seronegative and seropositive concentration components, prevalence (p) was estimated by two-component cluster analysis for the target IgG-Ptx data sets (*retrospective* SKI and PIENTER-2 data). Receiver operating characteristic (ROC) curves of the two-component distributions of IgG-Ptx and IgA-Ptx data sets of SKI, respectively, allowed the selection of model derived IgG-Ptx and IgA-Ptx cut-offs based on specificity and sensitivity to establish seroprevalence. Modelling was performed using software program “R”, version 3.1.2.

### Seroprevalence estimation based on cut-off analyses

Seroprevalence in *retrospective cases* and in the PIENTER-2 cohort was calculated using the pre-defined cut-off value for IgG-Ptx of 62.5 IU/ml to determine the proportion of infections in the past 12 months [23]. Alternatively, seroprevalence in *retrospective cases* was calculated by the use of a two-dimensional serodiagnostic profile that combined model-based experimental IgG-Ptx and IgA-Ptx cut-offs, optimized at 90% specificity.

## Results

### Increased IgG-Ptx seroprevalence in retrospective pertussis cases based on a classic cut-off method

**Fig 2:**
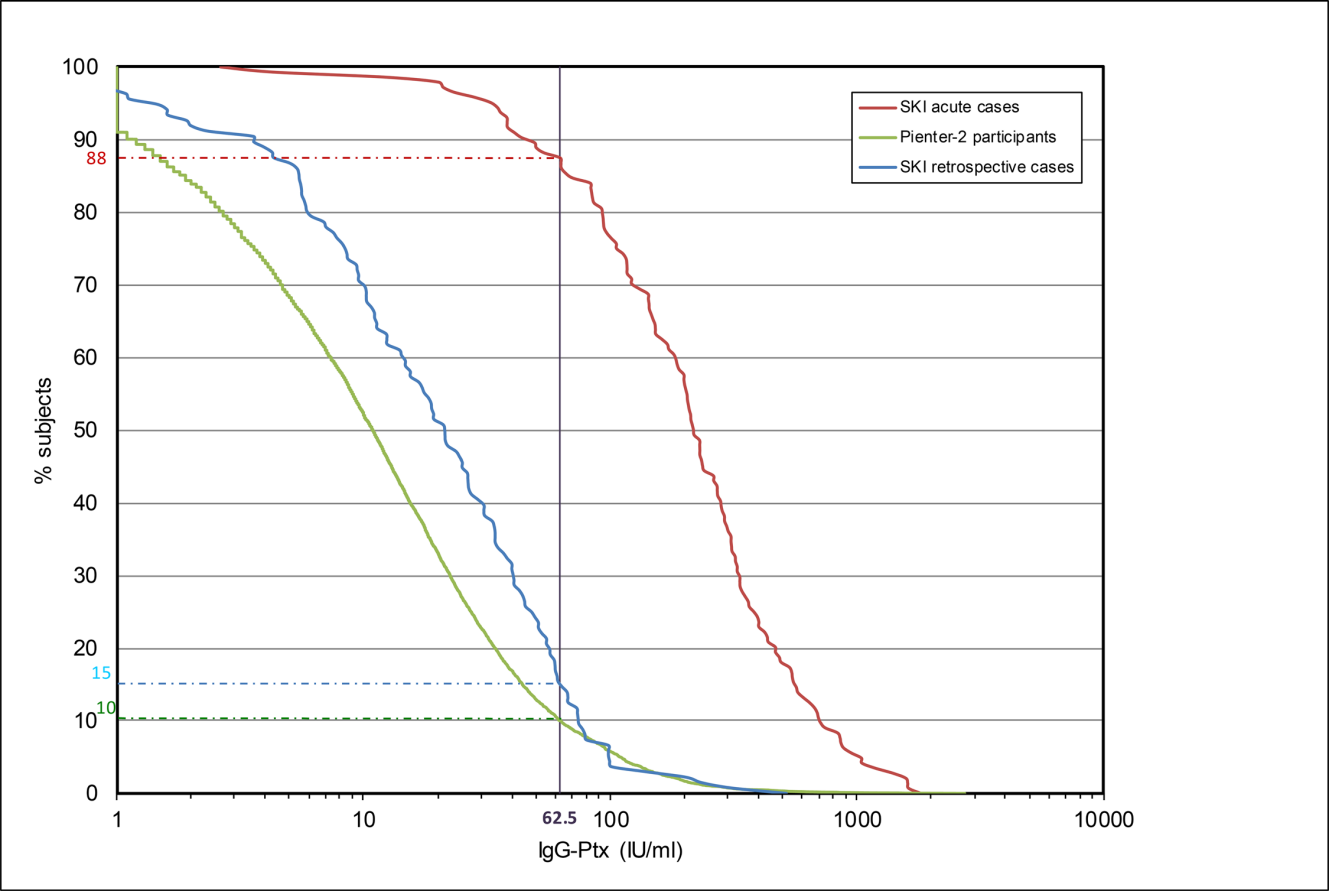
Reverse cumulative distribution curves of IgG-Ptx levels (IU/ml) measured in SKI and Pienter-2 cohorts. Shown are SKI *acute cases*, (dotted red line), SKI *retrospective cases* (blue line) and the population sample PIENTER-2 (green line). The vertical line indicates the pre-defined cut-off level of 62.5 IU/ml. The horizontal coloured lines indicate the percentages of the respective cohorts above the cut-off.

The IgG-Ptx antibody response profile of *retrospective cases* from the SKI study is depicted in a reverse cumulative response plot (Fig 2). Possible pertussis reinfected subjects are defined as the proportion of *retrospective cases* that displayed IgG-Ptx concentrations above the predefined cut-off level of 62.5 IU/ml [23]. Fifteen percent (21 of 137) of *retrospective cases* met these criteria (median 80 IU/ml, range 64–517 IU/ml, median time since known antigen exposure (τ): 47 months, range 24–135 months). This seroprevalence was higher than the 10% found for the population cohort PIENTER-2, comprising all age groups. (Fig 2). Of *acute cases* from the SKI study, 88% had plasma IgG-Ptx levels above the cut-off (median 218 IU/ml, range 3–1815 IU/ml) (Fig 2).

**Fig 3:**
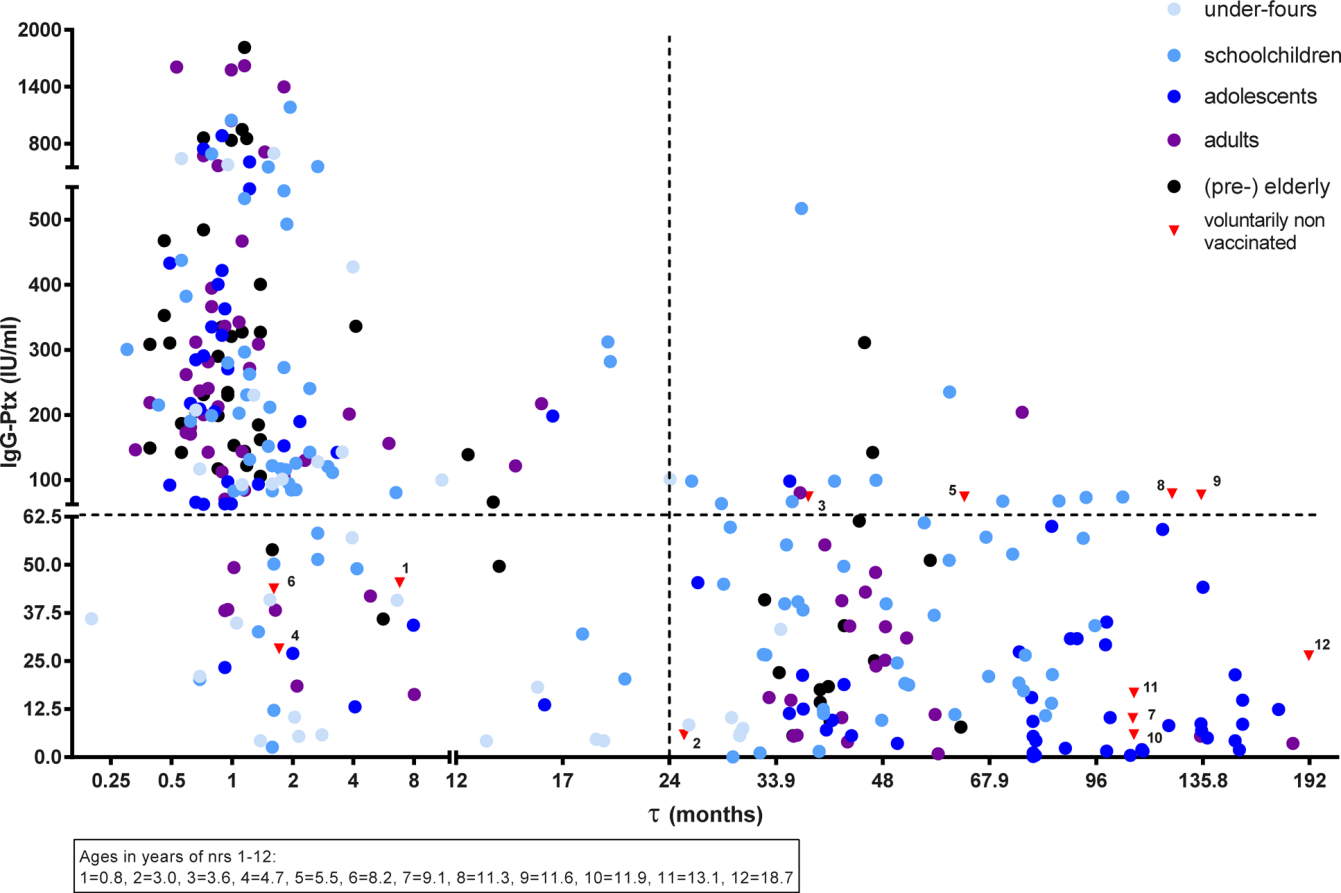
Levels of IgG-Ptx observed in time since last known pertussis antigen exposure in symptomatically infected cases of the SKI study. Cases were stratified according to age and vaccination status as is depicted in Fig 1.

To evaluate sero-responses of all SKI cases in more detail, individual IgG-Ptx levels were stratified according to exact τ (in months), age and vaccination history of the subject. A large variability in levels of IgG-Ptx can be seen in all cases (Fig 3). The twenty-one *retrospective cases* with IgG-Ptx level above the cut-off value of 62.5 IU/ml include participants from all age groups, with diverse vaccination histories. However, a relatively large part of this group (n = 12) consisted of children vaccinated with an acellular pertussis vaccine (aP) in the range 24–105 (median 44) months before blood sampling.

### Estimating IgG-Ptx seroprevalence in retrospective pertussis cases by a two-component cluster analysis

**Fig 4:**
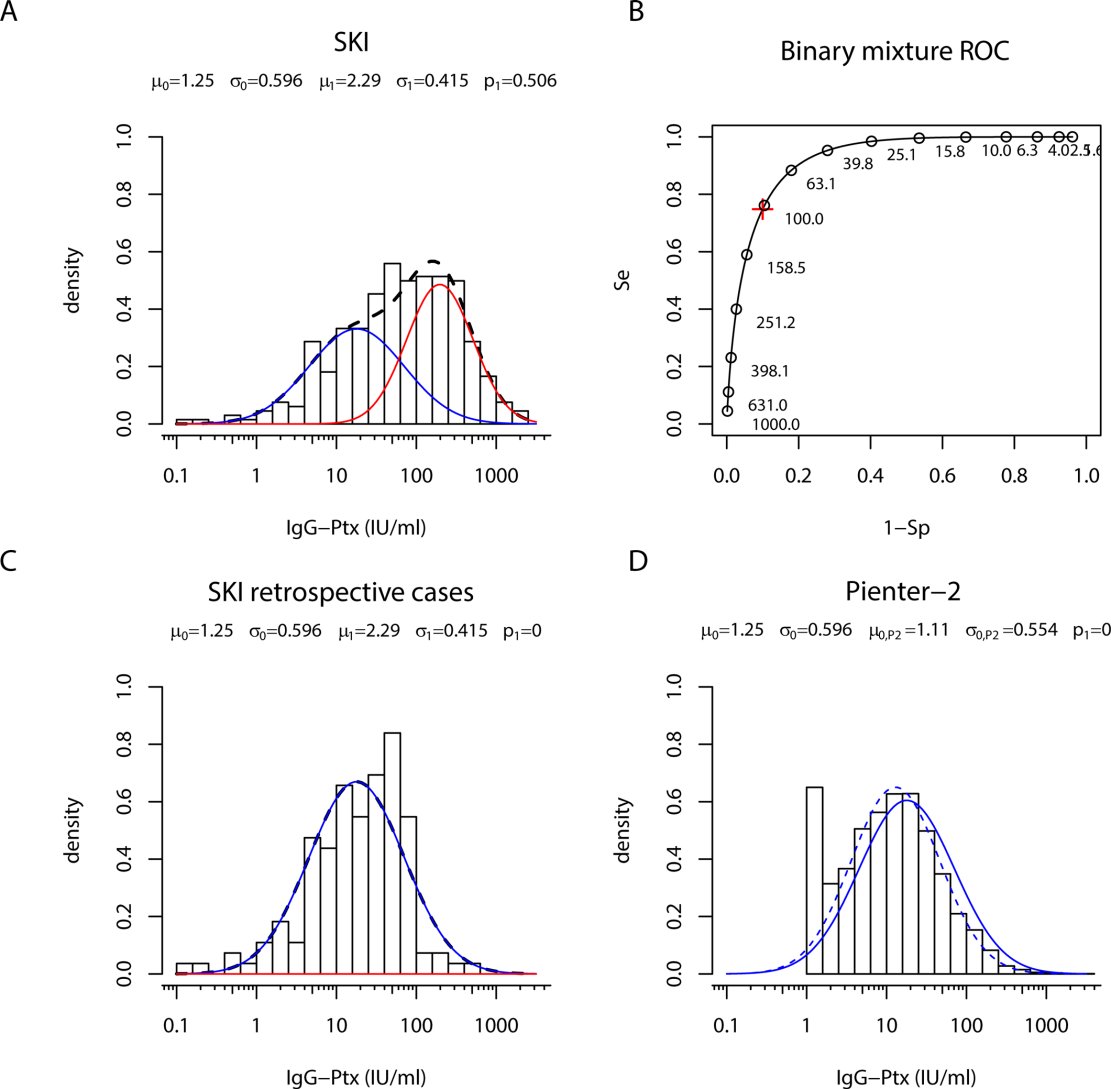
Two component cluster analysis of IgG-Ptx data sets from SKI and PIENTER-2. (A) Density distribution of IgG-Ptx (IU/ml) in samples obtained from all (ex-) pertussis cases in the SKI study (bars). Lines indicate the fitted negative (blue line), positive (red line) and combined (dotted black) components. (B) ROC curve for the model fitted in A. Se, sensitivity; 1-Sp, 1-specificity. Red cross: IgG-Ptx value corresponding to Sp = 90%. (C) Density distribution of IgG-Ptx (IU/ml) in samples from *retrospective cases* (bars). Lines indicate the fitted negative (blue line) component. No positive component was found. Model parameter values for IgG-Ptx were not affected when taking out data from paired samples (data not shown). (D) Density distribution of IgG-Ptx (IU/ml) in samples from the PIENTER-2 population cohort (bars). The dotted blue line indicates the fitted negative component from the PIENTER-2 data. No positive component was found. The overlay of the negative component from the retrospective *cases* is given as the blue line.

Next, we studied whether the elevated seroprevalence in *retrospective cases* as compared to population data could be confirmed by two-component cluster analysis [13]. When the model was fitted to IgG-Ptx data from all 331 SKI samples, two component distributions could indeed be identified (Fig 4A). The corresponding ROC diagram shows sensitivity and specificity for a range of cut-offs (Fig 4B). The pre-defined IgG-Ptx cut-off of 62.5 IU/ml corresponded to a specificity of 82% and sensitivity of 88.5% in our model.

When fixing μ (and σ) parameter values for the positive and negative components in this cluster model of all SKI IgG-Ptx data and applying these on *retrospective cases* only, the estimated prevalence was zero (Fig 4C), not confirming the proportion of reinfections based on the classical 62.5 IU/ml cut-off level. When performing two-component cluster analysis for the PIENTER-2 IgG-Ptx data, no clustering in two components could be seen either (Fig 4D). Hence, in both the clinical SKI study and the PIENTER-2 population survey setting, high levels of IgG-Ptx, indicating the positive component of recently seroconverted samples, were not well separable from lower levels representing the negative component. The analyses rather showed one normally distributed component of IgG-Ptx concentrations in both data sets, however with a higher median for the fitted data from the *retrospective cases*, refuting the use of the single population base IgG-Ptx cut-off 62.5 IU/ml to estimate seroprevalence in historical symptomatic cases.

### Finding an alternative parameter for recent seroconversion using two-component cluster analysis of IgA-Ptx levels

**Fig 5:**
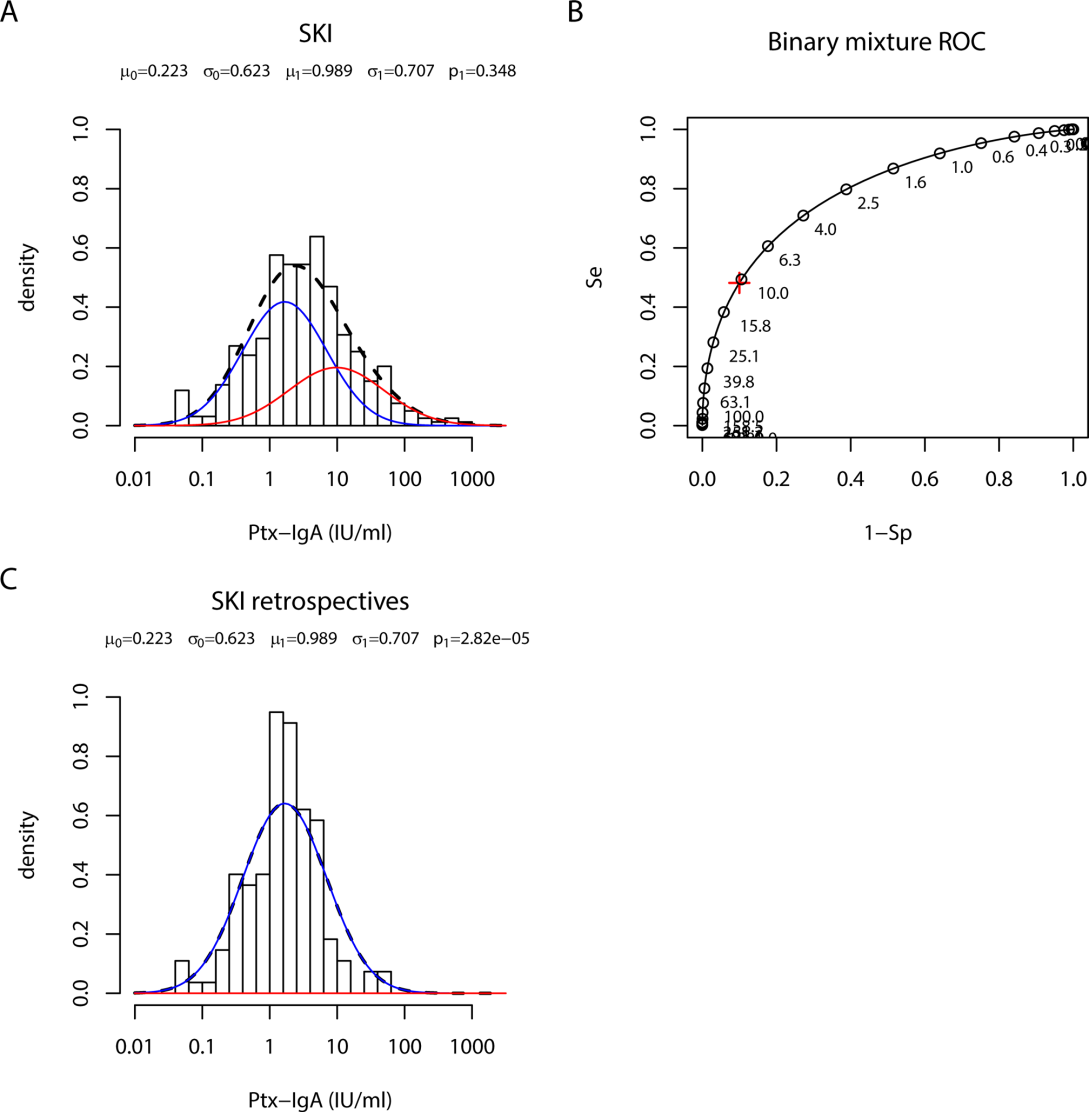
Two component cluster analysis of IgA-Ptx data from SKI cases. (A) Density distribution of IgA-Ptx (IU/ml) in samples obtained from all SKI cases (bars), including *acute* (τ < 2 months) and *non-acute* (τ >2 months) cases. Lines indicate the fitted negative (blue line) and positive (red line). (B) ROC curve for the model fitted in A and B. Se, sensitivity; 1-Sp, 1-specificity. Red cross: IgA-Ptx value corresponding to Sp = 90%. (C) Density distribution of IgA-Ptx (IU/ml) in samples from *retrospective cases* (bars). Lines indicate the fitted negative (blue line) component. No positive component was found. Model parameter values for IgA-Ptx were not affected when taking out data from paired samples (data not shown).

We then investigated whether IgA-Ptx has an added value in estimating proportions of reinfections in *retrospective cases*. When modelling Ptx specific IgA (IgA-Ptx) data from all SKI cases, samples from *acute cases* appeared to optimally fit the positive component of the model (Fig 5A). The corresponding ROC curve shows the sensitivity and specificity of the model for a range of cut-offs (Fig 5B). However, when applying the μ (and σ) parameter values obtained for the positive and negative components on the IgA-Ptx data from the *retrospective cases* only, zero prevalence was established (Fig 5C).

### Applying model derived optimized IgG-Ptx and IgA-Ptx cut-offs to estimate the proportion of reinfections in retrospective SKI cases

**Fig 6:**
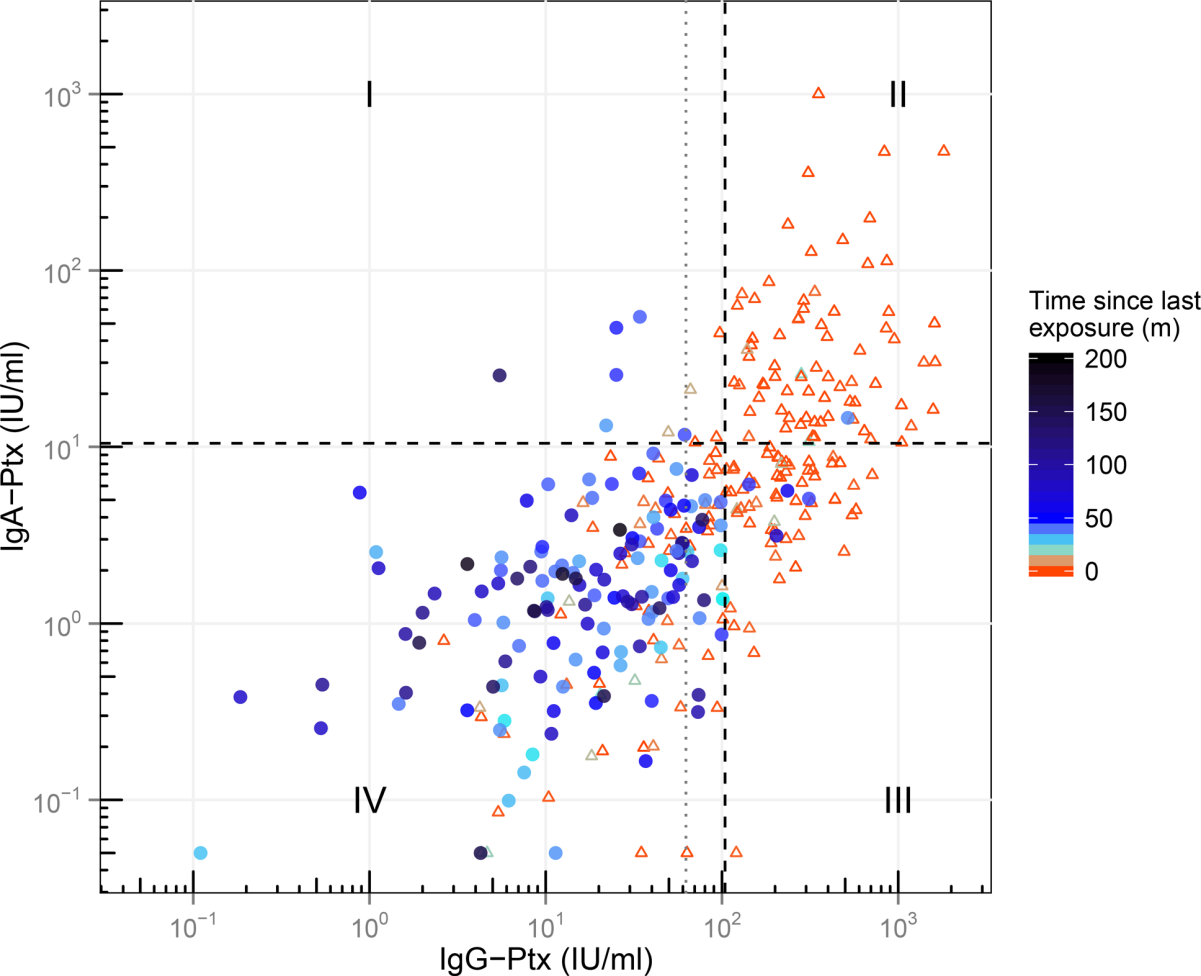
Two-dimensional serodiagnostic profile of all samples from SKI cases according to levels of IgA-Ptx and IgG-Ptx. Blue circles and red triangles represent *retrospective* and *convalescent cases* respectively. Grey dotted line represents 62.5 IU/ml IgG-Ptx cut-off. Black dotted lines represent model based cut-offs at 10.5 IU/ml and 104 IU/ml for IgA-Ptx and IgG-Ptx respectively. Samples are divided in 4 quadrants; Q_I_: IgG-Ptx^lo^IgA-Ptx^hi^, Q_II_: IgG-Ptx^hi^IgA-Ptx^hi^, Q_III_: IgG-Ptx^hi^IgA-Ptx^lo^, Q_IV_: IgG-Ptx^lo^IgA-Ptx^lo^. m = months.

Data from all SKI cases were then individually analysed in a two-dimensional (2D) IgG-Ptx and IgA-Ptx based serodiagnostic profile. For this model-derived IgG-Ptx and IgA-Ptx cut-offs were identified, optimized on a specificity of 90%, i.e. 104 IU/ml for IgG-Ptx (with a sensitivity of 75%) and 10.5 IU/ml for IgA-Ptx (with a sensitivity of 48%) (Fig 6). As expected, *convalescent cases* had the highest IgG-Ptx and IgA-Ptx levels and represented for the larger part the IgG-Ptx^hi^IgA-Ptx^hi^ upper right quadrant (Q_II_). Likewise, the IgG-Ptx^lo^IgA-Ptx^lo^ lower left quadrant (Q_IV_) represented primarily *retrospective cases* (τ ≥ 24 months): 126 of the 137 *retrospective cases* (92%) were located in this quadrant. The other *retrospective cases*, having either IgG-Ptx or IgA-Ptx levels or both above the newly defined cut-offs, were distributed over the remaining quadrants associated with different serological patterns occurring after seroconversion. Six *retrospective* cases (4.4%) fell in the IgG-Ptx^lo^IgA-Ptx^hi^ upper left quadrant (Q_I_). Because of the high IgA component we suggest these samples to represent a recent seroconversion (hence reinfection) but sampled too early for the IgG component to have risen concomitantly. These six cases were all (pre-) elderly. One *retrospective case* (0.7%) was located in Q_II_. We suggest this case to represent a renewed infection as well but sampled at a time interval having allowed elevation of the IgG component. Finally, four (2.9%) *retrospective cases* were located in the IgG-Ptx^hi^IgA-Ptx^lo^ lower right quadrant (Q_III_) for their elevated IgG-Ptx component only. This last profile can be interpreted in two ways: to reflect re-seroconversions of IgG-Ptx levels in the preceding year, however with the IgA-Ptx component already dropped below the cut-off. Alternatively, the profile might indicate slow decay of the IgG-Ptx component originated from the earlier diagnosed symptomatic pertussis episode. Taking a closer look at these last 4 cases; τ was relatively long (45–75 months; median 53 months) and the IgG-Ptx levels relatively high (142–311 IU/ml; median 220 IU/ml), making these values less likely to indicate remaining decay. By applying the model-based cut-offs of IgG-Ptx and IgA-Ptx the prevalence of reinfections was therefore determined by adding quadrants I, II and III, giving 8.0% in *retrospective cases*.

### Base levels of IgG-Ptx long term after symptomatic seroconversion

**Fig 7:**
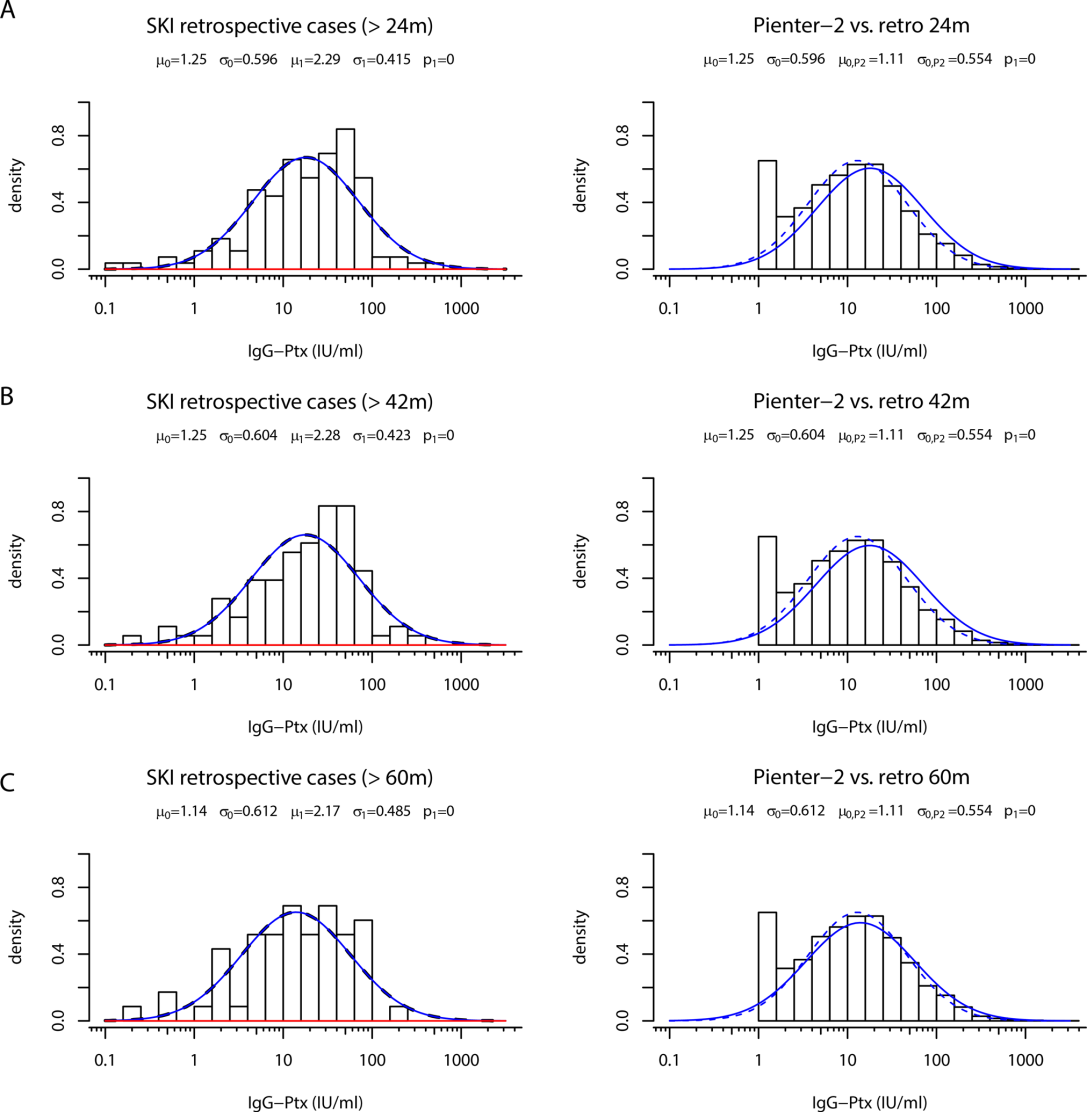
Time shift analysis of the negative IgG-Ptx component of SKI *retrospective cases* and overlay with PIENTER-2 data. Left panels: Density distribution of IgG-Ptx (IU/ml) in samples obtained from *retrospective cases* (bars) having τ >24 months (A), τ >42 months (B), or τ >60 months (C), respectively. Lines indicate the fitted negative (blue line) and positive (red line). Right panels: Density distribution of IgG-Ptx (IU/ml) in samples obtained from the PIENTER-2 study (bars + dotted line), the blue line indicates the time shifted overlay of the *retrospective cases* indicated in the left panel.

The observation that the median IgG-Ptx level of the modelled PIENTER-2 population data was lower than its counterpart of *retrospective cases* (Fig 4D) could indicate that IgG-Ptx levels elevated after symptomatic infections do not drop back to population base-levels, but to an elevated post-infection base-line. To investigate this hypothesis time-shifted IgG-Ptx density distribution profiles from *retrospective cases* were compared to the density distribution of the PIENTER-2 population cohort. Not confirming the hypothesis, gradual decline towards the PIENTER-2 median IgG-Ptx level was observed for *retrospective cases* when being selected on more distant time intervals after their symptomatic laboratory confirmed diagnosis: >24, >42, >60, months post-exposure, respectively (Fig 7). The negative component in the mixture curve of the SKI cohort with cases >60 months post exposure was almost equal to the PIENTER-2 curve. This indicated that decline of IgG-Ptx levels in ex-pertussis cases is ongoing for at least 3,5 years.

## Discussion

Serodiagnostic methods for single point IgG-Ptx values using a previously defined cut-off and a two-component cluster model fall short in estimating the proportion of pertussis reinfections in previously symptomatically infected individuals. The continuum of intermediate IgG-Ptx levels between clear seropositive and seronegative opposites, observed in both *retrospective* and population datasets, is the main obstacle preventing these methods to work. This was the first major outcome of our analysis of the stand-alone IgG-Ptx dataset from (ex-) symptomatic pertussis cases in the SKI study. In this situation, values above a predefined cut-off suffer the risk of being interpreted as new sero-conversions, while ongoing decay can be involved. This is the first study to demonstrate that the continuous distribution shape of the IgG-Ptx response prevents seroclassification for both *retrospective cases* and population data when using a population based cut-off and two-component cluster analysis. Hence, modelling of pertussis IgG-Ptx based sero-responses by two-component cluster analysis and the use of cut-off values only seems to work well in the early diagnostic setting [13]. Baughman *et al*. did propose a diagnostic IgG-Ptx cut-off in a US population survey by using a four component mixture model. However, moderate levels of IgG-Ptx were also seen, likely caused by either a gradual decay or recent infection or vaccination, and were defined as indeterminate for acute *B*. *pertussis* infection [33].

As a second outcome of our study, we found that two-component mixture analysis of the IgG-Ptx data set combined with a second IgA-Ptx dataset, using data from both *convalescent and retrospective cases*, did help to extract meaningful knowledge from the continuum of seroresponses of *retrospective cases*. This included insight into their proportion of reinfections and the relatively long decay period in their IgG-Ptx levels, the latter being at least 3.5 years, which is longer than previously reported [11, 34].

Using our new cohort optimized 2D serological cut-offs, we estimated that the proportion of reinfections in the cohort of *retrospective cases* was 8.0%. This lower (re)infection prevalence in earlier proven cases as compared to the general population was found despite the fact that the SKI study took place in a time frame with relatively more pertussis notifications on a year base than in the PIENTER-2 time frame. This suggests that an even lower proportion of reinfections in the SKI cohort might have been anticipated, had the pertussis epidemiology matched. Natural infection has been suggested to confer a longer period of protection by inducing qualitatively different immune mechanisms compared to vaccination only [6, 35]. This was recently supported in the baboon model, showing superior protection from colonisation and disease upon challenge in animals recovered from primary infection [36]. We propose that the (slightly) lower occurrence of reinfections estimated for the retrospective SKI cases as compared to population prevalence might relate to a differential immune status as a result of their (additional) symptomatic natural infection. Evidence for this needs more study of prevalence and immunology in cohorts of proven ex-patients. The identified reinfected cases did not report recent pertussis symptoms, implying that reinfections are mostly asymptomatic. A direct 2D comparison of seroprevalence with the population test was not possible since IgA-Ptx data were not generated for PIENTER-2. Our model optimized cut-offs are comparable with literature. The IgG-Ptx cut-off of 104 IU/ml approaches the 125 IU/ml cut-off for a infection in the past six months [11, 23]; however, we take our newly defined cut-off to evaluate sero-reconversions in the past year. Our IgA-Ptx cut-off of 10.5 IU/ml lies in the range 10–20 IU/ml, recommended by Guiso et al [37]. Other studies also have pointed out the additional value of IgA-Ptx measurements in serodiagnostics [28, 31, 38, 39]; the notion being that exposure to *B*. *pertussis* antigens through infection will lead to both IgA-and IgG-Ptx production [30] while exposure by (aP) vaccination alone will generate IgG but less IgA to Ptx [40]. However, this is the first cross sectional study of ex-pertussis cases that uses the faster decay of IgA-Ptx levels compared to IgG-Ptx levels, in combination with cut-offs optimized by two-component cluster analyses to determine a more credible estimate of the prevalence of reinfections in ex-pertussis cases than is possible when using a predetermined cut-off based solely on IgG-Ptx.

The modelling procedures and comparison of IgG-Ptx density distributions from time-shifted *retrospective cases* with population data gave us valuable information on the decay rates of IgG-Ptx in symptomatic ex-pertussis cases. Noteworthy was the gradual shift of *retrospective cases* towards the base-line distribution of population data in 5 years’ time. Most studies on the decay of IgG-Ptx, both after infection and vaccination agree on an initial peak in antibody levels that is reached within a few weeks. The subsequent decline has been described to be more or less exponential, involving a relatively fast decline 6–12 months after exposure [11, 41, 42]. Alternatively, the decline could be biphasic: a rapid decay after the initial peak and a slow phase some months later, resulting in measurable antibody-levels lasting several years [31, 34, 43]. We observed a slow decay of IgG-Ptx in symptomatically infected cases, matching a biphasic shape. Although IgG-Ptx peak levels show large individual variation, we did find on average higher maximum IgG-Ptx levels in the SKI cohort compared to the PIENTER-2 cohort (containing symptomatic but mostly asymptomatic cases) when considering all samples with IgG-Ptx levels >62.5 IU/ml (data not shown). This could mean that symptomatically compared to asymptomatically infected cases reach higher IgG-Ptx levels in the acute phase and therefore take longer to decay to base levels. An additional cause for the relatively high IgG-Ptx levels in our *retrospective cases* could be previous vaccination. The SKI cohort has an oversampling of children, most of whom had their child-hood vaccinations. Recent vaccination can confound serological and oral fluid assays targeting anti-Ptx IgG antibodies as a marker of recent infection [34, 44]. While others recommend one year [37], we chose for *retrospective cases* a limit of at least 2 years between either date of diagnosis or last pertussis vaccination and date of blood sampling, in order to avoid vaccination effects or remaining decay from the initial infection. The decay patterns in the SKI IgG-Ptx dataset show that this limit was most likely (still) too short. For diagnostic purposes, in the circumstances of a natural infection preceding an aP vaccination we recommend the confounding period to be at least 3 years after vaccination. The use of our two-dimensional (2D) serodiagnostic profile still has its limitations. Q_III_ is a challenging quadrant, cases herein either reflect re-seroconversions of IgG-Ptx levels in the preceding year, with a declined IgA-Ptx component, or a slow decay of the IgG-Ptx component originated from an earlier pertussis exposure. Cases falling into this quadrant need to be evaluated separately to determine which of these is the most likely scenario. Also, the use of IgA-Ptx in serodiagnostics may have its restraints. Others have seen either little or no response of IgA-Ptx in children < 12 years old [31, 45] or a stepwise increase of IgA-Ptx with age in pertussis cases [39]. Although there are IgA-Ptx responders in all of the SKI age groups, our SKI data did confirm this latter trend. This was illustrated by Q_I_ in our 2D serodiagnostic profile only containing elderly (>52 years old) *retrospective cases*. While children may fail to raise detectable IgA-Ptx, elderly may have a higher maintenance of the IgA-Ptx response. Considering this age-effect, the 4.4% reinfections found in Q_I_, and hence the total percentage of reinfections of 8.0% in *retrospective cases*, could be an overestimation. On the other hand, an eventual incapacity in IgA generation in younger *retrospective* cases could lead to an underestimation of reinfections in this 2D analysis. This issue needs further investigation. Finally, the 2D profiling method for IgG-Ptx and IgA-Ptx evaluation in ex-cases needs to be further refined by careful calculation of confidence intervals and maximized specificity and sensitivity values for cut-off levels. Until then any reinfection estimate based on cut-offs should be taken with caution, even when combining two parameters.

In conclusion, our study first revealed the shortcomings of regular serodiagnostic IgG-Ptx based methods for the calculation of proportions of reinfections in former symptomatic pertussis cases. Applying a 2D profile of model based IgG-Ptx and IgA-Ptx cut-offs can help solve this problem to a certain degree. We suggest more research on the use of IgA-Ptx in serodiagnostics and to apply this in population studies. Ex-patients seem to be at lower risk for seroconversion than the general population, substantiating the hypothesis that a clinical symptomatic pertussis infection provides a relatively long period of protection to renewed infection.

## Supporting Information

S1 Table IgG-Ptx and IgA-Ptx data of PIENTER-2 and SKI samples. (PDF)
