# Supplementary material for: The Use of Innovative Two-Component Cluster Analysis and Serodiagnostic Cut-Off Methods to Estimate Prevalence of Pertussis Reinfections
Source: PLoS One. 2016 Feb 5;11(2):e0148507. doi: 10.1371/journal.pone.0148507 (PMC4743910; doi:10.1371/journal.pone.0148507)
Supplement: S1 Table — (PDF) [file pone.0148507.s001.pdf]

SKI cohort of 292 (ex) symptomatic pertussis patients, n=331 samples

USN	Age (yrs)	Age group	Mnths p.exp.	last exposure Vacc. or Inf.	IgA-Ptx (IU/ml)	IgG-Ptx (IU/ml)
CI55099	1.0	under-fours	0.20	Vacc	0.2	36.0
HU11101	10.3	schoolchildren	0.30	Inf	14.6	300.8
HU21801	54.5	adults	0.33	Inf	37.7	146.5
HU21001	67.0	(pre-) elderly	0.39	Inf	357.5	308.6
GH16201	26.9	adults	0.39	Inf	8.2	219.0
HU21201	66.6	(pre-) elderly	0.39	Inf	41.1	149.0
HU07501	8.5	schoolchildren	0.43	Inf	6.7	215.2
HU21601	59.1	(pre-) elderly	0.46	Inf	8.1	467.7
HU20901	73.9	(pre-) elderly	0.46	Inf	1000.0	352.9
HU11001	13.3	adolescents	0.49	Inf	58.4	433.3
HU20801	78.9	(pre-) elderly	0.49	Inf	20.6	310.4
HU10901	12.5	adolescents	0.49	Inf	9.2	92.0
HU16901	52.4	adults	0.53	Inf	50.1	1606.9
HU01001	3.1	under-fours	0.56	Inf	12.3	642.4
HU01021	7.9	schoolchildren	0.56	inf	5.0	437.6
HU21761	63.9	(pre-) elderly	0.56	inf	9.9	186.9
HU21701	62.4	(pre-) elderly	0.56	Inf	32.4	142.3
HU08301	9.8	schoolchildren	0.59	Inf	19.0	382.3
HU16601	48.1	adults	0.59	Inf	2.1	261.9
HU20001	33.6	adults	0.59	Inf	22.4	172.9
HU11701	12.4	adolescents	0.62	Inf	16.1	217.6
HU05501	6.9	schoolchildren	0.62	Inf	3.4	190.4
HU15412	54.0	adults	0.62	inf	9.1	181.0
HU15401	53.0	adults	0.62	Inf	22.7	170.5
HU15501	49.6	adults	0.66	Inf	8.2	311.9
HU10501	16.2	adolescents	0.66	Inf	6.3	284.7
HU00501	2.3	under-fours	0.66	Inf	8.8	207.3
HU20301	59.8	(pre-) elderly	0.66	Inf	6.9	205.6
HU11301	11.4	adolescents	0.66	Inf	2.7	65.8
HU16101	49.2	adults	0.69	Inf	182.2	236.8
HU11401	11.1	adolescents	0.69	Inf	8.7	209.2
HU00601	3.8	under-fours	0.69	Inf	7.7	116.8
HU00701	3.3	under-fours	0.69	Inf	0.2	21.0
CI57299	3.8	schoolchildren	0.69	Vacc	0.5	20.2
HU22101	66.5	(pre-) elderly	0.72	Inf	113.0	861.4
HU10801	12.3	adolescents	0.72	Inf	22.8	743.5
HU15861	51.3	adults	0.72	inf	108.8	672.1
HU22161	67.7	(pre-) elderly	0.72	inf	148.9	484.5
HU10701	15.1	adolescents	0.72	Inf	67.5	290.8
HU21501	66.1	(pre-) elderly	0.72	Inf	8.2	231.0
HU15801	54.5	adults	0.72	Inf	24.9	200.2
HU15201	18.9	adolescents	0.72	Inf	3.4	62.6
HU22401	53.2	adults	0.76	Inf	24.9	281.6
HU15701	49.7	adults	0.76	Inf	14.6	240.5
HU16801	42.5	adults	0.76	Inf	6.6	142.8
HU05701	10.0	schoolchildren	0.79	Inf	197.5	690.2
HU16001	51.7	adults	0.79	Inf	42.0	395.0
SP16301	52.8	adults	0.79	Inf	49.0	366.4
SP10201	15.4	adolescents	0.79	Inf	13.8	335.3
HU05101	4.6	schoolchildren	0.79	Inf	3.4	198.8
HU11201	13.0	adolescents	0.82	Inf	5.2	205.0
HU17201	42.7	adults	0.85	Inf	17.9	568.5
SP10401	14.7	adolescents	0.85	Inf	14.8	400.7

HU22001	78.8	(pre-) elderly	0.85	Inf	60.6	290.2
HU15901	48.8	adults	0.85	Inf	43.0	212.5
HU20201	71.8	(pre-) elderly	0.85	Inf	28.7	199.0
HU20501	68.5	(pre-) elderly	0.85	Inf	23.2	117.3
HU11901	16.0	adolescents	0.89	Inf	58.3	883.8
HU10601	14.6	adolescents	0.89	Inf	8.1	422.1
HU21101	61.9	(pre-) elderly	0.89	Inf	11.4	335.1
HU11601	14.8	adolescents	0.89	Inf	4.8	322.2
HU17301	28.8	adults	0.89	Inf	5.5	112.5
HU16401	18.2	adolescents	0.92	Inf	13.8	362.9
HU15101	37.3	adults	0.92	Inf	6.8	336.2
HU16501	40.7	adults	0.92	Inf	10.6	70.3
HU11801	11.9	adolescents	0.92	Inf	2.5	63.2
HU16701	22.4	adults	0.92	Inf	6.6	38.1
HU10301	12.1	adolescents	0.92	Inf	8.8	23.3
HU00101	3.5	under-fours	0.95	Inf	4.4	576.6
HU05201	8.6	schoolchildren	0.95	Inf	13.3	279.9
HU12001	13.7	adolescents	0.95	Inf	52.9	270.8
HU21401	67.9	(pre-) elderly	0.95	Inf	20.7	234.6
HU20101	64.3	(pre-) elderly	0.95	Inf	12.7	230.1
HU10101	12.9	adolescents	0.95	Inf	44.1	97.2
HU15601	23.6	adults	0.95	Inf	1.2	38.4
HU07011	39.9	adults	0.99	inf	16.3	1578.6
HU07001	6.9	schoolchildren	0.99	Inf	10.6	1047.9
HU17001	47.9	adults	0.99	Inf	17.2	1042.7
HU20601	63.5	(pre-) elderly	0.99	Inf	471.0	834.2
HU21301	73.9	(pre-) elderly	0.99	Inf	127.6	320.8
HU11501	12.5	adolescents	0.99	Inf	0.1	63.3
HU20701	58.2	(pre-) elderly	1.02	Inf	69.0	153.1
HU05301	8.4	schoolchildren	1.02	Inf	3.3	82.9
HU15301	23.0	adults	1.02	Inf	5.4	49.3
HU00901	0.5	under-fours	1.05	Inf	0.1	34.9
HU50899	30.2	adults	1.08	Inf	28.1	342.8
HU06801	9.3	schoolchildren	1.08	Inf	5.3	202.6
HU22701	81.2	(pre-) elderly	1.12	Inf	40.8	949.9
HU51499	43.4	adults	1.12	Inf	21.9	467.2
HU21901	72.7	(pre-) elderly	1.12	Inf	7.4	327.4
HU22301	58.3	adults	1.12	Inf	3.7	143.7
HU01301	3.7	under-fours	1.12	Inf	11.3	93.0
HU53499	5.5	schoolchildren	1.12	Inf	3.6	86.2
HU23001	89.7	(pre-) elderly	1.15	Inf	472.6	1815.1
HU53299	40.2	adults	1.15	Inf	30.2	1620.9
HU53599	9.5	schoolchildren	1.15	Inf	18.0	532.5
HU06201	6.4	schoolchildren	1.15	Inf	4.1	296.5
HU20401	65.2	(pre-) elderly	1.15	Inf	15.8	144.1
HU51199	47.2	adults	1.15	Inf	4.0	84.2
HU08401	8.4	schoolchildren	1.18	Inf	3.0	230.9
HU22201	75.8	(pre-) elderly	1.18	Inf	47.0	854.5
HU22261	75.7	(pre-) elderly	1.18	inf	63.3	122.2
HU53199	15.1	adolescents	1.22	Inf	35.1	606.3
HU53099	11.0	adolescents	1.22	Inf	4.1	547.3
HU52999	48.4	adults	1.22	Inf	53.4	271.6
HU06501	7.2	schoolchildren	1.22	Inf	4.9	262.8
HU05401	9.2	schoolchildren	1.22	Inf	5.7	131.4
HU01201	2.1	under-fours	1.28	Inf	7.2	230.6
HU51299	36.3	adults	1.35	Inf	7.3	308.8

HU22901	84.6	(pre-) elderly	1.35	Inf	86.1	184.8
HU53399	16.3	adolescents	1.35	Inf	7.4	93.6
HU06001	9.7	schoolchildren	1.35	Inf	1.3	32.6
HU22601	82.4	(pre-) elderly	1.38	Inf	24.8	400.5
SP52199	56.0	(pre-) elderly	1.38	Inf	11.4	327.1
HU22501	78.7	(pre-) elderly	1.38	Inf	19.0	162.1
HU22801	76.5	(pre-) elderly	1.38	Inf	5.6	105.8
HU00401	2.0	under-fours	1.38	Inf	0.3	4.3
HU52899	46.0	adults	1.45	Inf	6.9	712.5
HU08501	4.8	schoolchildren	1.51	Inf	14.3	553.8
HU07701	4.2	schoolchildren	1.51	Inf	0.7	151.8
HU06401	9.3	schoolchildren	1.54	Inf	1.8	211.9
HU00801	1.1	under-fours	1.54	Inf	0.8	40.9
HU07601	7.3	schoolchildren	1.58	Inf	4.2	122.1
HU00301	1.2	under-fours	1.58	Inf	0.3	93.9
HU06601	6.4	schoolchildren	1.58	Inf	0.7	83.5
HU51699	58.5	(pre-) elderly	1.58	Inf	4.2	53.9
HU05801	7.3	schoolchildren	1.58	Inf	0.8	2.6
CI72199	1.2	under-fours	1.61	Vacc	11.0	698.0
HU54399	5.0	schoolchildren	1.61	Inf	2.6	50.2
HU06101	8.2	choolchildren_ non vac	1.61	Inf	8.6	43.8
HU07401	4.8	schoolchildren	1.61	Inf	1.1	12.2
HU51599	30.0	adults	1.64	Inf	2.8	38.2
HU07801	4.7	choolchildren_ non vac	1.71	Inf	2.5	28.2
SP51999	10.3	schoolchildren	1.74	Inf	1.0	117.1
HU53699	1.1	under-fours	1.77	Inf	1.1	101.2
HU22461	48.0	adults	1.81	inf	30.1	1398.3
HU06901	8.4	schoolchildren	1.81	Inf	23.4	544.3
HU08001	8.3	schoolchildren	1.81	Inf	3.1	272.9
HU51899	19.2	adolescents	1.81	Inf	6.2	152.5
HU51399	48.3	adults	1.81	Inf	7.5	106.1
GH07901	7.3	schoolchildren	1.84	Inf	7.4	115.9
HU07201	8.4	schoolchildren	1.87	Inf	2.6	493.1
HU07301	7.5	schoolchildren	1.91	Inf	4.7	94.6
HU54099	9.1	schoolchildren	1.94	Inf	13.1	1185.3
HU07101	7.6	schoolchildren	1.97	Inf	7.0	84.8
SP52399	13.7	adolescents	2.00	Inf	2.2	27.0
GH50399	0.8	under-fours	2.04	Inf	0.1	10.4
HU08201	9.7	schoolchildren	2.07	Inf	22.4	125.6
HU05901	8.2	schoolchildren	2.07	Inf	8.3	84.9
HU52499	28.3	adults	2.10	Inf	3.5	18.5
CI54499	0.9	under-fours	2.14	Inf	0.1	5.4
HU52799	16.8	adolescents	2.17	Inf	2.9	189.9
HU52299	53.6	adults	2.30	Inf	73.5	130.0
HU54199	10.4	schoolchildren	2.43	Inf	7.8	240.8
HU54299	8.0	schoolchildren	2.43	Inf	4.6	142.8
HU54999	10.2	schoolchildren	2.66	Inf	6.0	559.2
HU52599	3.6	under-fours	2.66	Inf	4.5	128.2
HU54899	10.2	schoolchildren	2.66	Inf	0.3	58.2
HU52699	9.7	schoolchildren	2.66	Inf	3.2	51.4
CI54799	0.7	under-fours	2.79	Inf	0.2	5.8
CI58099	4.2	schoolchildren	2.99	Vacc	0.1	120.5
HU71999	4.9	schoolchildren	3.15	Inf	1.2	111.5
HU50599	11.0	adolescents	3.32	Inf	11.4	142.2
CI54599	0.7	under-fours	3.52	Vacc	0.9	143.1
HU50699	40.4	adults	3.81	Inf	2.4	201.3

HU54699	3.3	under-fours	3.94	Inf	0.8	57.0
CI53999	0.7	under-fours	3.98	Vacc	8.8	427.4
HU50099	15.0	adolescents	4.07	Inf	0.5	13.1
HU51799	65.3	(pre-) elderly	4.11	Inf	75.6	336.3
HU51099	4.1	schoolchildren	4.17	Vacc	1.0	49.0
HU53899	56.6	adults	4.86	Inf	4.5	41.9
HU50999	72.9	(pre-) elderly	5.62	Inf	4.9	35.9
GH50199	42.9	adults	6.01	Inf	4.8	156.4
HU71899	4.3	schoolchildren	6.51	Inf	4.7	80.7
CI56799	1.6	under-fours	6.60	Vacc	0.2	40.8
CI72299	0.8	under-fours_ non vacc	6.80	Inf	0.6	45.4
HU50799	13.0	adolescents	7.95	Inf	3.7	34.3
HU50499	37.2	adults	8.02	Inf	4.8	16.3
CI56599	2.0	under-fours	11.01	Vacc	1.6	100.1
HU55699	65.4	(pre-) elderly	12.48	Inf	35.5	139.0
CI57599	2.0	under-fours	13.24	Inf	0.3	4.2
HU22702	82.2	(pre-) elderly	13.54	Inf	21.1	66.2
HU22602	83.4	(pre-) elderly	13.80	Inf	12.1	49.6
HU22302	59.5	adults	14.55	Inf	4.4	121.5
CI57499	2.3	under-fours	15.64	Inf	0.2	18.2
HU56299	38.9	adults	15.84	Inf	8.0	217.3
GH55399	16.5	adolescents	16.00	Inf	1.3	13.6
HU56399	11.7	adolescents	16.43	Inf	3.8	198.2
CI70999	5.4	schoolchildren	18.10	Vacc	0.5	32.0
CI57399	2.5	under-fours	18.92	Vacc	0.1	4.7
CI57199	2.6	under-fours	19.38	Vacc	0.1	4.3
CI63699	5.7	schoolchildren	19.65	Vacc	10.7	312.1
CI63799	5.5	schoolchildren	19.81	Vacc	25.8	281.9
CI71399	5.6	schoolchildren	20.76	Vacc	0.4	20.3
CI56999	3.2	under-fours	24.05	Vacc	1.4	101.0
CI56899	3.0	under-fours_ non vacc	25.17	Inf	0.3	5.8
CI56699	3.1	under-fours	25.56	Vacc	0.2	8.4
CI64299	6.0	schoolchildren	25.79	Vacc	2.6	98.2
HU56199	11.3	adolescents	26.32	Inf	2.3	45.4
CI64799	6.4	schoolchildren	28.42	Vacc	2.6	63.7
CI70399	6.4	schoolchildren	28.62	Vacc	0.7	45.0
CI71499	6.3	schoolchildren	29.24	Vacc	1.8	59.8
CI71099	3.4	under-fours	29.37	Vacc	1.4	10.3
GH62499	6.7	schoolchildren	29.50	Vacc	0.1	0.1
CI57999	3.6	under-fours	30.16	Inf	0.4	5.6
CI58299	3.5	under-fours	30.23	Vacc	0.1	6.2
CI57099	3.6	under-fours	30.46	Inf	0.1	7.5
CI71599	6.4	schoolchildren	32.20	Vacc	2.5	1.1
HU06802	12.0	schoolchildren	32.56	Inf	0.7	26.7
HU55299	61.7	(pre-) elderly	32.69	Inf	4.0	40.9
CI70199	6.7	schoolchildren	32.82	Vacc	0.6	26.6
HU16502	43.4	adults	33.18	Inf	2.3	15.5
HU22102	69.3	(pre-) elderly	34.27	Inf	13.2	22.0
CI58199	3.9	under-fours	34.46	Vacc	2.3	33.2
CI65099	7.0	schoolchildren	34.89	Vacc	1.5	39.9
HU05702	12.9	schoolchildren	35.09	Inf	7.5	55.2
HU11502	15.4	adolescents	35.42	Inf	0.1	11.4
HU10802	15.2	adolescents	35.48	Inf	3.6	98.4
GH16202	29.8	adults	35.61	Inf	0.6	14.8
CI72099	6.9	schoolchildren	35.75	Vacc	4.6	66.8
HU20502	72.2	(pre-) elderly	35.84	Inf	2.4	5.6

HU55899	48.2	adults	35.91	Inf	0.2	5.5
HU16702	25.3	adults	36.34	Inf	1.0	5.7
HU06502	10.1	schoolchildren	36.44	Inf	1.2	40.4
HU57699	42.0	adults	36.70	Inf	5.0	80.2
HU61799	7.7	schoolchildren	36.86	Vacc	14.6	517.4
HU11402	14.1	adolescents	36.99	Inf	0.9	21.3
HU06902	11.3	schoolchildren	37.03	Inf	1.1	38.3
HU55999	13.5	adolescents	37.06	Inf	0.4	12.5
CI57799	3.6	under-fours_ non vacc	37.68	Inf	1.1	74.6
HU05802	10.4	schoolchildren	39.03	Inf	0.4	1.5
HU21702	65.6	(pre-) elderly	39.16	Inf	6.5	17.6
HU21770	67.1	(pre-) elderly	39.16	inf	1.9	14.3
HU05502	10.2	schoolchildren	39.56	Inf	2.1	12.4
HU05902	11.4	schoolchildren	39.59	Inf	2.0	11.3
HU56099	37.8	adults	39.75	Inf	2.6	55.2
HU11802	15.2	adolescents	39.95	Inf	0.7	7.1
HU21202	69.9	(pre-) elderly	40.21	Inf	5.1	18.4
HU21902	76.0	(pre-) elderly	40.44	Inf	2.6	9.4
GH55199	12.0	adolescents	40.74	Inf	1.7	9.6
CI70499	7.1	schoolchildren	41.04	Vacc	4.9	98.3
HU15870	54.8	adults	42.02	inf	9.2	40.7
HU15802	57.9	adults	42.02	Inf	6.1	10.3
CI70599	7.7	schoolchildren	42.28	Vacc	1.4	49.6
GH55599	15.9	adolescents	42.32	Inf	1.4	18.9
HU21002	69.5	(pre-) elderly	42.35	Inf	54.6	34.2
HU15302	26.5	adults	42.81	Inf	1.0	4.0
HU16902	56.0	adults	43.11	Inf	2.9	34.1
HU10902	16.0	adolescents	43.37	Inf	2.0	5.6
HU20602	67.1	(pre-) elderly	44.45	Inf	11.7	61.4
HU21102	65.6	(pre-) elderly	45.24	Inf	5.1	311.3
HU16402	21.9	adults	45.37	Inf	3.4	42.9
HU20302	63.6	(pre-) elderly	46.46	Inf	6.1	142.2
HU20702	62.0	(pre-) elderly	46.72	Inf	25.6	25.1
HU16002	55.5	adults	46.88	Inf	5.0	48.0
CI64899	8.3	schoolchildren	46.95	Vacc	0.9	99.5
HU15902	52.6	adults	46.95	Inf	6.2	23.7
CI71799	7.9	schoolchildren	47.84	Vacc	2.7	9.6
HU16102	53.2	adults	48.33	Inf	47.3	25.2
SP16302	56.8	adults	48.40	Inf	7.1	33.9
CI64499	7.9	schoolchildren	48.53	Vacc	0.4	39.9
CI64199	8.0	schoolchildren	50.30	Vacc	1.4	24.5
HU11302	15.6	adolescents	50.33	Inf	0.3	3.6
CI71199	8.1	schoolchildren	51.55	Vacc	0.4	19.2
HU15502	53.9	adults	51.88	Inf	3.1	31.0
CI72399	8.1	schoolchildren	52.21	Vacc	0.5	18.8
CI64099	5.7	schoolchildren	54.93	Vacc	4.7	60.9
HU20102	68.9	(pre-) elderly	55.98	Inf	4.4	51.2
GH62399	8.6	schoolchildren	56.71	Vacc	0.2	36.9
HU15202	23.6	adults	56.90	Inf	0.8	11.1
HU63499	42.7	adults	57.46	Inf	5.5	0.9
CI64599	8.8	schoolchildren	59.57	Vacc	2.0	51.2
CI64699	9.5	schoolchildren	59.63	Vacc	5.6	235.1
HU63399	9.0	schoolchildren	60.65	Vacc	0.3	11.1
HU61899	64.8	(pre-) elderly	61.87	Inf	5.0	7.8
CI63999	5.5	choolchildren_ non vac	62.56	Inf	3.5	74.1
GH60599	9.8	schoolchildren	67.12	Vacc	1.7	57.2

CI70699	9.5	schoolchildren	67.68	Vacc	0.7	21.0
CI70799	9.9	schoolchildren	70.87	Vacc	6.9	67.4
GH60199	9.4	schoolchildren	73.17	Vacc	1.4	52.8
CI64999	10.1	schoolchildren	74.68	Vacc	2.0	19.3
GH62299	15.8	adolescents	74.78	Inf	1.4	27.4
HU61599	45.9	adults	75.47	Inf	3.1	203.9
GH62999	10.3	schoolchildren	75.83	Vacc	1.0	17.3
CI70899	10.0	schoolchildren	76.22	Vacc	2.5	26.5
GH60399	11.1	adolescents	77.83	Inf	1.7	15.5
GH62599	11.6	adolescents	78.16	Inf	0.5	9.3
GH63099	14.2	adolescents	78.23	Inf	1.7	5.4
GH63199	15.4	adolescents	78.23	Inf	2.1	1.1
GH62899	12.3	adolescents	78.23	Inf	0.4	0.2
HU63299	11.5	adolescents	78.82	Inf	0.3	0.5
GH60299	16.5	adolescents	78.92	Inf	1.5	4.3
CI71299	7.0	schoolchildren	81.41	Inf	0.2	10.8
CI66599	11.2	schoolchildren	83.02	Vacc	4.1	14.0
GH62699	13.6	adolescents	83.09	Inf	2.8	60.0
CI66799	10.7	schoolchildren	83.25	Vacc	1.8	21.5
CI66999	11.2	schoolchildren	85.09	Vacc	2.3	67.6
HU61399	15.9	adolescents	86.84	Inf	1.5	2.3
GH61699	15.5	adolescents	88.28	Inf	2.8	30.8
CI67299	11.5	adolescents	90.22	Vacc	1.3	30.8
CI66699	11.7	schoolchildren	92.03	Vacc	2.5	56.9
CI67499	12.1	schoolchildren	92.88	Vacc	0.3	73.1
CI67199	11.8	schoolchildren	95.64	Vacc	0.7	34.2
GH61199	14.6	adolescents	98.93	Inf	1.3	29.2
GH60799	12.2	adolescents	99.29	Inf	1.4	35.1
GH60699	14.2	adolescents	99.29	Inf	0.9	1.6
GH61499	15.3	adolescents	100.44	Inf	1.2	10.3
CI67599	12.1	schoolchildren	104.64	Vacc	0.4	73.7
GH61099	17.1	adolescents	107.30	Inf	0.5	0.5
CI70299	9.1	choolchildren_ non vac	108.16	Inf	1.2	10.1
GH62099	13.1	choolchildren_ non vac	108.55	Inf	1.3	16.7
GH61999	11.9	choolchildren_ non vac	108.55	Inf	0.6	5.9
GH60499	12.2	adolescents	111.38	Inf	1.2	2.0
GH63599	15.6	adolescents	111.77	Inf	0.4	1.6
GH65299	11.0	adolescents	119.13	Vacc	2.9	59.2
GH61299	11.5	adolescents	121.56	Inf	2.1	8.2
CI67099	11.3	choolchildren_ non vac	122.84	Inf	1.4	79.1
GH65399	52.8	adults	134.77	Inf	25.4	5.5
GH65499	18.0	adolescents	134.97	Inf	1.2	8.7
CI67399	11.6	choolchildren_ non vac	135.00	Inf	3.9	77.3
CI66899	12.3	adolescents	135.26	Vacc	1.8	6.9
GH65799	17.2	adolescents	135.82	Inf	1.2	44.2
GH65699	16.5	adolescents	137.76	Inf	0.4	5.0
GH66099	16.2	adolescents	150.77	Inf	0.4	21.4
GH66199	14.1	adolescents	150.77	Inf	0.1	4.3
GH66499	17.9	adolescents	152.84	Inf	0.8	1.9
CI66399	14.0	adolescents	154.55	Vacc	1.8	14.8
CI66299	14.0	adolescents	154.55	Vacc	1.2	8.6
GH65899	17.3	adolescents	173.60	Inf	1.9	12.4
GH65599	26.2	adults	181.95	Inf	2.2	3.6
GH65999	18.7	adolescents_ non vacc	191.54	Inf	3.4	26.4

PIENTER-2 data : Random Survey of Dutch population without oversampling
n= 5740 (2608 =male, 3132=female)

SampleCode	Age (yrs)	IgG-Ptx (EU/ml)
13600002	47.6	88.6
13600007	74.3	150.1
13600008	74.3	29.9
13600011	49.3	2.4
13600013	0.9	3.7
13600014	29.4	5.7
13600015	10.4	1
13600016	6.1	1.4
13600019	6.6	2.7
13600020	11.3	80.6
13600021	0.9	447.9
13600022	12.3	18.3
13600024	6.3	7.8
13600031	14.7	36.1
13600033	16.2	3.7
13600035	9.7	18
13600039	8.6	1
13600041	9.5	1
13600049	51.7	6.2
13600050	58.9	50.7
13600056	45.9	1
13600057	59.9	13
13600058	4.9	1.7
13600062	45.9	218.2
13600066	41.1	15.3
13600069	13.1	1
13600071	46.5	40.4
13600073	43.3	1.9
13600074	56.3	77.2
13600076	77.2	16.6
13600079	75.1	27.7
13600085	71.6	12.8
13600088	69.5	6.2
13600094	18.0	1
13600096	71.7	18.1
13600099	66.3	10.1
13600101	65.1	33.6
13600103	65.9	23.6
13600104	61.9	14
13600105	63.4	3.8
13600106	17.4	1
13600107	64.4	15.6
13600109	62.3	1
13600110	63.7	19.2
13600113	56.2	10.4
13600117	60.9	3.7
13600119	59.7	14
13600120	28.7	93.6
13600122	53.6	89.7
13600124	53.5	1
13600127	4.6	1.4
13600128	3.6	37.6
13600129	22.6	3.4

13600130	39.0	4.3
13600135	29.0	7.4
13600136	14.6	7
13600137	72.3	7.2
13600138	78.9	10.8
13600139	0.5	11.8
13600140	75.6	10
13600141	3.1	1.3
13600144	65.7	4.9
13600146	17.3	21.9
13600147	61.4	6.1
13600148	15.3	1
13600151	28.0	2.5
13600154	47.9	56.2
13600157	1.6	3.5
13600159	0.5	65.9
13600161	0.9	14.5
13600165	48.4	3.5
13600166	24.4	139.2
13600168	0.4	288.3
13600169	5.6	5.9
13600171	11.1	6.5
13600173	6.5	2.6
13600174	9.8	7.8
13600178	37.1	1
13600179	14.0	21.7
13600185	65.0	11.1
13600187	39.1	19.8
13600188	9.5	1
13600195	26.3	14
13600198	2.6	1.5
13600199	40.8	9.6
13600200	1.4	26.1
13600201	44.6	12.2
13600202	27.7	15
13600203	58.5	20.4
13600206	51.8	20.3
13600207	49.4	31.3
13600208	49.7	1.4
13600209	56.6	57.5
13600211	32.2	5.8
13600213	70.6	9.5
13600218	34.9	1.6
13600223	43.4	8
13600224	69.7	1.5
13600225	19.2	10.6
13600226	36.7	34.5
13600229	12.6	1.9
13600232	11.1	2.6
13600234	8.8	6.8
13600240	0.5	80.9
13600244	34.8	20.5
13600246	3.6	1
13600248	45.8	7.6
13600249	9.1	1
13600253	40.7	37.3
13600255	50.6	26.4

13600258	3.4	1.3
13600262	79.0	23.6
13600264	77.7	3.7
13600265	77.0	1
13600266	0.5	1
13600267	2.9	1
13600268	0.7	20
13600269	0.8	58.6
13600271	72.2	126.6
13600272	70.0	6.2
13600274	69.1	8.7
13600275	72.7	41.4
13600278	71.0	39
13600282	68.2	16.3
13600283	66.6	10.4
13600284	66.8	53.3
13600287	15.4	170.7
13600288	65.7	119
13600289	64.8	1
13600296	45.9	12.7
13600306	63.9	2
13600316	4.3	2.7
13600323	65.9	3.6
13600327	39.0	6
13600328	43.6	5.5
13600331	5.0	7.9
13600333	9.4	16.7
13600335	21.0	6.8
13600336	3.3	1
13600337	3.3	1
13600339	73.9	2.6
13600341	58.7	17.5
13600342	11.1	1.8
13600344	0.6	33.5
13600346	52.2	46.7
13600348	5.5	1
13600355	0.9	7.2
13600357	4.4	2.3
13600359	13.4	2.1
13600360	40.9	1.4
13600361	9.7	101.2
13600362	13.1	14.4
13600363	38.8	59.7
13600364	17.0	2.4
13600366	43.2	14
13600369	29.3	14
13600372	33.1	92.3
13600373	22.3	5.7
13600374	54.5	7
13600375	54.7	32.9
13600377	6.5	23.3
13600378	24.1	34.9
13600379	44.1	16.8
13600380	46.9	17
13600436	17.7	1
13600439	48.0	29.7
13600440	12.2	1.1

13600441	49.2	1
13600443	15.5	26.3
13600444	30.3	19.7
13600445	57.6	9.9
13600447	23.0	2.2
13600450	34.1	11.1
13600451	60.1	5.5
13600454	64.3	29.8
13600456	63.8	7.3
13600458	71.2	14
13600462	2.7	13.6
13600466	33.7	152.3
13600467	23.9	18.3
13600468	67.7	66.2
13600469	17.4	21.1
13600473	39.8	6.3
13600477	58.6	33.9
13600478	33.7	8.3
13600483	43.2	17.6
13600488	66.7	3.6
13600493	0.5	43.9
13600495	16.6	6.1
13600497	33.3	7.7
13600498	3.0	1
13600503	79.6	91.3
13600504	76.1	1.5
13600505	77.9	4
13600506	0.4	92.4
13600507	76.4	33.2
13600508	75.9	16.7
13600509	3.4	1.7
13600510	3.4	1.3
13600515	64.7	42.1
13600517	60.3	2.5
13600518	61.7	5.3
13600521	63.4	4
13600527	71.6	34.7
13600528	68.8	8.7
13600530	7.8	24.9
13600533	65.8	4.5
13600535	16.2	6.1
13600537	13.5	18.3
13600542	36.5	11.9
13600543	57.9	11.4
13600549	47.9	7.8
13600550	20.4	30.4
13600551	0.8	2.8
13600559	73.2	1
13600560	1.0	10.8
13600604	53.6	3
13600606	11.5	1
13600607	55.9	44.8
13600608	0.3	1.9
13600616	43.2	9.4
13600618	32.8	1.2
13600619	7.0	17.4
13600620	40.9	5.2

13600623	48.1	11.3
13600624	78.1	10.5
13600625	27.5	6.4
13600627	42.6	4.1
13600629	73.3	20.6
13600630	71.1	70.2
13600631	64.2	158.5
13600632	67.9	13.8
13600633	60.6	51
13600634	69.5	2.6
13600677	68.8	13.2
13600678	68.1	15.6
13600679	70.4	1.2
13600683	3.1	1
13600686	72.1	45
13600690	0.2	1
13600692	57.0	14.4
13600693	2.8	11.1
13600694	75.4	12.1
13600697	70.1	23.5
13600698	70.4	30.1
13600699	2.2	5.2
13600700	4.0	188.4
13600701	61.7	8.3
13600706	60.9	16.9
13600709	1.1	195.8
13600712	13.1	205.3
13600719	66.9	49.3
13600721	65.6	1474.4
13600723	23.6	3.4
13600724	58.4	1
13600725	50.3	61.5
13600727	23.8	2.4
13600728	43.1	5.7
13600750	63.9	9.3
13600751	2.6	2.2
13600754	11.5	2.1
13600756	12.1	8.3
13600760	12.8	1
13600762	5.8	94.7
13600764	11.5	14.7
13600765	15.7	2.5
13600766	11.7	1.1
13600769	12.0	1
13600771	15.7	1.1
13600779	16.6	18.9
13600782	45.6	42.7
13600787	20.2	33.9
13600788	57.7	315.3
13600789	7.1	1
13600790	77.5	16.2
13600792	2.1	1.9
13600795	2.7	1
13600797	75.1	98.8
13600800	37.9	1
13600801	33.9	4.3
13600804	17.1	5.4

13600805	63.4	136.5
13600806	65.6	4.9
13600811	76.1	18.6
13600812	55.4	15
13600815	2.7	1
13600816	71.7	22.6
13600817	3.4	60.4
13600818	71.9	35
13600820	67.8	103.8
13600823	65.5	173.3
13600824	62.5	18.2
13600825	61.5	16
13600826	1.6	31.2
13600828	48.3	27.9
13600829	15.4	6.4
13600831	60.5	20.6
13600836	78.2	6.6
13600838	20.8	38.5
13600842	16.2	2.8
13600844	45.5	8.6
13600845	33.8	28.1
13600846	46.3	3.2
13600847	56.8	45.4
13600851	26.4	7.1
13600852	46.5	45.2
13600855	61.1	11.7
13600856	1.1	45.7
13600861	43.6	35
13600864	61.1	13.4
13600865	3.0	1
13600866	0.4	116.9
13600867	53.2	27.8
13600869	28.5	42.3
13600874	57.0	35.3
13600876	47.8	32.5
13600877	21.4	22.6
13600882	0.9	23.3
13600885	10.5	31.4
13600886	56.0	7.2
13600888	2.6	1
13600893	11.5	38.7
13600894	15.2	69.3
13600895	13.4	28.9
13600897	8.4	184.1
13600898	14.0	1
13600899	0.8	23.3
13600901	8.5	8.4
13600903	1.6	1.5
13600905	6.7	53
13600907	7.1	3
13600908	13.5	3.5
13600911	7.8	1.9
13600912	52.8	13.1
13600914	52.8	34.7
13600919	45.5	31.1
13600923	18.1	5.9
13600924	42.3	21.7

13600927	38.1	10.4
13600931	1.2	111.7
13600932	1.3	93.9
13600934	75.8	6
13600937	5.4	8.5
13600939	11.0	8.3
13600940	12.1	10
13600941	7.9	1
13600942	6.4	90.1
13600943	9.0	3.8
13600944	17.2	9.7
13600949	76.1	8.7
13600950	75.4	24
13600951	0.2	5.8
13600954	0.4	506.5
13600955	5.4	5.9
13600958	6.8	20.3
13600959	2.7	1.3
13600960	13.0	7.2
13600962	14.7	1
13600966	16.5	1.7
13600967	71.3	36.3
13600970	65.7	34.3
13600978	4.2	21.8
13600980	0.8	24.5
13600981	70.5	226.2
13600982	68.2	3.9
13600983	4.4	3.1
13600988	4.5	38.2
13600989	65.7	4.9
13600991	5.0	1.7
13600992	12.8	5.1
13600993	63.7	12.7
13600996	61.0	28.5
13600997	62.2	16.3
13600999	59.3	1.7
13601000	54.8	1
13601001	56.4	318.9
13601005	10.4	1.5
13601006	18.3	6.8
13601009	25.9	2.2
13601014	20.2	21.1
13601018	16.1	30.9
13601020	17.2	31.6
13601023	46.7	13.9
13601027	0.9	9.9
13601028	54.0	15.2
13601031	46.1	41.4
13601033	46.1	18.6
13601034	29.1	29
13601037	40.1	2.3
13601038	40.1	2.5
13601039	6.6	1.1
13601041	8.1	3.3
13601042	22.3	1
13601044	5.3	6.9
13601045	54.2	28.1

13601048	27.6	1.1
13601053	39.6	31.1
13601054	53.7	28.9
13601056	20.3	149.7
13601057	8.0	6.1
13601084	32.0	8.8
13601087	3.8	6.9
13601089	38.8	31.8
13601090	41.1	1
13601093	48.5	3.7
13601095	1.0	276.1
13601096	5.5	24
13601232	30.8	12.4
13601234	20.2	82.9
13601235	5.0	1.8
13601236	63.8	19.4
13601237	50.8	5.8
13601240	20.1	21.7
13601242	0.6	212.9
13601243	77.4	12.3
13601246	72.1	5.9
13601248	68.3	77.1
13601252	41.4	2.9
13601253	24.3	18.2
13601254	49.4	4
13601256	78.1	8.4
13601259	36.2	16.3
13601260	56.1	12.2
13601263	0.6	60.9
13601265	79.5	1.3
13601267	66.3	9.7
13601269	61.2	17.8
13601276	46.6	22.4
13601277	24.4	35.9
13601278	58.5	90.1
13601279	76.8	11.1
13601281	50.2	12
13601298	5.2	1.7
13601299	64.5	194.5
13601301	1.3	29.5
13601305	67.9	8.8
13601309	11.1	3.1
13601310	0.9	11.1
13601313	2.3	1
13601314	1.2	30.7
13601316	10.9	78.8
13601319	17.5	18
13601320	47.8	30.8
13601321	22.2	1.9
13601322	9.5	18.9
13601325	0.7	10.1
13601328	61.0	17.6
13601330	75.7	14.9
13601333	43.7	1.1
13601347	77.6	13.4
13601349	73.2	1.5
13601350	0.6	73

13601352	72.4	182.1
13601353	72.7	1.4
13601358	67.3	14.2
13601361	66.7	6
13601362	66.8	12.9
13601363	15.2	108.8
13601365	65.6	31.2
13601366	64.9	4.1
13601367	65.9	1
13601368	62.8	27.4
13601371	1.0	155.5
13601372	60.2	7
13601373	62.2	3.5
13601376	64.6	10.9
13601378	37.2	29.3
13601379	45.9	6.3
13601380	46.6	29.5
13601381	53.7	43.5
13601383	50.1	12.5
13601384	48.6	1
13601385	13.7	16.7
13601386	7.5	3.8
13601387	53.1	25.8
13601390	57.4	33.4
13601392	36.7	21.4
13601394	49.3	11
13601398	2.9	1
13601400	73.7	12.5
13601403	59.1	10.5
13601408	3.9	1
13601409	8.4	1
13601410	20.5	2.3
13601413	22.1	1
13601420	28.0	50.3
13601424	4.0	31.9
13601427	12.0	1
13601429	7.7	18.8
13601430	6.7	1
13601431	3.6	1
13601434	13.1	8.1
13601435	9.3	6.8
13601436	1.0	424.2
13601437	4.1	299.7
13601438	14.5	1
13601440	16.4	8.7
13601442	15.7	6.2
13601443	48.5	1.4
13601444	39.6	186.8
13601446	55.6	246.2
13601447	43.7	6.8
13601457	39.5	11
13601458	41.4	10.6
13601461	45.6	5.5
13601464	38.8	11.6
13601465	51.2	31.9
13601466	74.2	16
13601467	46.7	17.3

13601472	66.4	26.8
13601475	74.6	91.3
13601476	62.1	84.2
13601477	72.4	9
13601482	72.5	11.4
13601484	71.2	5.5
13601485	3.4	6.9
13601486	0.9	11.3
13601488	69.7	47.9
13601489	68.3	72.7
13601493	67.7	17.3
13601494	15.7	2.4
13601495	15.9	22.5
13601498	61.3	18.1
13601502	61.4	8.4
13601508	43.0	5.1
13601511	47.0	28.4
13601512	48.0	59
13601513	0.5	7.7
13601518	68.9	7
13601520	5.6	9.6
13601521	40.4	42.5
13601523	68.6	7.2
13601524	46.9	5.7
13601527	16.2	13.6
13601529	78.6	80.8
13601531	54.2	1
13601533	76.0	1
13601536	6.1	2.5
13601537	7.3	2.7
13601538	45.5	13.8
13601541	0.9	104
13601544	0.3	19.1
13601545	61.7	14.7
13601546	77.6	12.3
13601549	49.5	3.8
13601550	2.1	2
13601552	0.7	32.3
13601554	77.7	57.5
13601555	4.2	6.3
13601556	14.1	1
13601557	15.7	110
13601558	39.9	5.9
13601560	13.5	3.2
13601561	54.5	22.8
13601563	47.7	3.4
13601567	32.3	14.9
13601568	22.8	16.1
13601572	18.1	1
13601575	41.6	2.4
13601577	14.1	5.6
13601578	38.9	48.2
13601583	3.2	1.3
13601584	13.5	35.5
13601585	53.0	16.4
13601586	0.9	21.7
13601588	0.3	30.2

13601592	75.5	1
13601600	79.7	41.2
13601603	77.8	27.6
13601606	75.7	12.4
13601612	11.0	10
13601617	11.8	1
13601624	70.3	70.1
13601626	68.3	32.2
13601629	9.4	7.2
13601630	37.8	16
13601632	63.1	11.8
13601636	14.6	3.3
13601637	13.4	1
13601639	13.6	1
13601641	74.4	19.1
13601646	2.1	5.8
13601647	68.9	28.1
13601650	73.7	40.6
13601651	69.4	38
13601653	0.5	45.6
13601654	4.8	2.9
13601655	51.8	1.2
13601657	0.5	19.5
13601658	12.8	5.3
13601659	46.2	2.2
13601662	51.5	14
13601671	59.3	7.2
13601673	22.8	71
13601674	15.3	7.1
13601679	61.3	15.1
13601680	60.5	6.2
13601683	39.6	1.3
13601690	43.4	7.7
13601694	17.7	1
13601695	16.6	3.5
13601705	49.9	1.1
13601708	22.8	1
13601715	25.1	18.8
13601720	71.3	3.6
13601721	71.0	7.4
13601723	62.3	12.2
13601725	44.7	3.3
13601729	8.4	3.2
13601731	2.9	1
13601732	0.9	46.3
13601733	4.2	61.9
13601736	75.8	6.1
13601742	67.7	12
13601745	62.2	20.8
13601747	61.2	9.8
13601748	67.3	24.6
13601749	62.6	24.6
13601752	44.9	117.4
13601753	16.1	58.8
13601756	18.0	1.7
13601759	33.1	1.6
13601761	21.6	36.1

13601762	48.9	32.1
13601763	35.8	11.2
13601764	42.0	5.1
13601768	38.9	142.6
13601779	58.2	19.2
13601781	48.5	16.5
13601783	14.3	6.6
13601785	11.0	3.6
13601786	0.5	46.3
13601787	71.3	8.7
13601789	29.3	64.6
13601790	32.1	6.3
13601796	0.4	104.2
13601797	2.7	1
13601799	57.7	29.7
13601800	0.8	27
13601801	43.4	17
13601803	4.7	1.4
13601804	1.0	12.1
13601805	50.2	4
13601806	11.1	19.4
13601807	10.3	28.6
13601808	12.0	11.6
13601809	12.6	20.3
13601810	13.7	2.9
13601811	12.6	4.4
13601812	50.2	22.5
13601816	9.1	1.6
13601817	50.4	1
13601819	44.6	43.9
13601821	19.0	2.1
13601826	21.1	6
13601827	34.9	5.8
13601828	21.2	6.5
13601829	39.2	11.1
13601830	51.3	11.5
13601835	74.1	2
13601838	18.6	7.9
13601839	12.0	15.2
13601842	2.3	7.6
13601845	2.8	4
13601851	73.6	26.2
13601852	56.5	5.5
13601853	73.8	29.2
13601855	74.2	36.8
13601856	72.8	56.1
13601858	67.3	19.7
13601859	63.8	8
13601863	64.6	8.4
13601866	62.7	1.3
13601875	59.2	6.7
13601879	48.7	4.1
13601880	45.8	4.5
13601881	45.7	10.7
13601882	35.6	7.3
13601883	26.7	10.1
13601885	59.5	20.2

13601888	45.3	5.6
13601889	33.7	82.8
13601896	34.7	2.4
13601899	29.4	38.7
13601900	47.9	5.6
13601901	48.4	27.1
13601902	13.0	1
13601905	12.4	1.1
13601907	0.9	20.1
13601908	28.7	4.7
13601909	60.3	14.8
13601912	30.7	18.6
13601917	1.2	57.1
13601922	5.8	3.5
13601924	10.8	1
13601925	23.5	79.3
13601929	5.7	44.4
13601930	10.9	1.3
13601931	7.4	1
13601934	7.1	22.1
13601935	27.7	27.4
13601936	0.5	67.4
13601938	9.4	1.2
13601939	9.7	1
13601941	3.5	1
13601942	14.7	5.1
13601946	0.9	5.6
13601947	4.8	30.2
13601951	14.5	1
13601952	19.8	2.1
13601953	4.3	14.7
13601954	4.5	104.3
13601956	49.9	9.4
13601960	35.6	1
13601962	5.3	1.1
13601963	22.3	7
13601964	50.4	14.7
13601966	11.4	27.3
13601971	39.1	28.7
13601972	18.8	13.7
13601974	15.8	27.6
13601978	1.8	35.4
13601979	30.0	29.1
13601980	57.9	6.9
13601982	0.3	1.5
13602040	24.0	19.6
13602042	1.2	80.8
13602043	43.3	1
13602044	44.3	9.1
13602047	0.6	18.8
13602052	44.1	475.9
13602055	6.2	2.5
13602057	40.8	26.4
13602058	56.7	99.9
13602060	0.3	1.8
13602434	51.9	14.3
13602435	0.1	17.6

13602438	79.5	283.8
13602439	0.1	1
13602441	0.2	1.6
13602442	64.3	5.2
13602444	74.1	21.3
13602445	70.0	33
13602447	2.5	1.4
13602453	0.9	28.1
13602455	62.4	90.7
13602459	76.8	18.5
13602460	62.9	8.5
13602461	71.9	2.6
13602462	13.0	1
13602463	17.0	95.7
13602465	73.3	35.9
13602467	65.6	3.9
13602468	16.3	1.2
13602469	15.4	3.8
13602470	66.3	2.4
13602478	44.2	4.6
13602482	40.8	2.3
13602483	47.3	42.6
13602484	48.4	7.4
13602485	52.8	5.4
13602486	37.6	24.4
13602487	50.4	55.2
13602489	43.1	197.3
13602491	31.0	23.1
13602498	52.4	81.5
13602501	14.4	2.7
13602502	23.5	1
13602505	11.1	13.9
13602508	0.9	8.8
13602509	66.5	8.2
13602512	7.3	2.5
13602513	7.3	3.1
13602517	0.7	11.5
13602525	7.2	1
13602528	15.5	13.7
13602529	5.8	1.5
13602532	13.8	3.5
13602536	25.3	12.9
13602538	3.7	1
13602543	51.9	8.2
13602546	23.1	56.1
13602551	0.7	24.4
13602552	21.2	2.1
13602553	29.5	24
13602554	41.4	9.9
13602556	39.6	33
13602557	44.5	2.4
13602560	29.3	12.4
13602563	0.8	10.7
13602567	54.3	3.4
13602569	27.2	26.6
13602571	45.1	8.1
13602574	12.7	9.3

13602578	25.7	3.2
13602579	25.0	1
13602580	77.0	22
13602587	75.6	222.8
13602589	71.1	9
13602592	27.6	8.8
13602597	35.4	22.3
13602598	23.8	26.2
13602601	52.2	3
13602604	35.4	2.9
13602606	18.4	1
13602607	48.7	67.5
13602609	72.3	9.1
13602612	21.4	4.6
13602613	55.5	9.3
13602616	4.5	7
13602618	0.4	70.6
13602619	0.4	102.2
13602622	3.4	1
13602623	64.1	13.9
13602627	4.2	12.7
13602629	10.9	3
13602630	74.5	12.9
13602632	11.3	40.1
13602635	13.6	17.7
13602640	16.5	106.5
13602644	10.4	64.4
13602645	54.9	1.7
13602646	43.9	47
13602653	0.8	23.6
13602660	29.3	20.7
13602661	8.6	1
13602662	1.8	50.4
13602663	49.0	11.2
13602664	11.7	35.3
13602667	14.1	18.1
13602669	0.7	23
13602671	76.3	8.2
13602672	32.3	58.1
13602673	62.6	30.4
13602676	77.2	87.8
13602679	61.4	8.7
13602681	77.3	12.8
13602687	75.8	16.9
13602691	61.8	27.5
13602693	79.0	2.1
13602695	69.0	42
13602696	13.4	2.2
13602697	14.0	3.3
13602705	48.6	12.8
13602708	43.4	2
13602712	30.6	22.4
13602719	49.2	6.5
13602723	38.8	1.6
13602726	8.1	1
13602730	65.2	123.2
13602731	10.6	1.6

13602733	38.6	1.2
13602735	46.8	47.4
13602737	72.8	15
13602738	24.3	16.8
13602739	6.8	7.9
13602741	12.0	1.6
13602743	80.0	1
13602745	0.4	26.7
13602748	0.6	136
13602749	1.0	64
13602751	69.3	27.3
13602752	62.7	1.7
13602757	4.5	36.8
13602759	3.6	2
13602762	36.7	2.5
13602771	11.0	36.1
13602773	1.0	14.6
13602774	18.1	17.6
13602775	53.3	1.1
13602776	25.9	23.8
13602777	54.4	1
13602780	55.1	14.7
13602782	55.6	122.3
13602783	39.0	12.6
13602785	35.7	9.6
13602786	21.6	46.6
13602787	53.2	6.6
13602790	57.6	13.9
13602794	56.2	14.1
13602796	48.4	13
13602797	29.5	14
13602799	12.0	2.3
13602802	67.4	30.8
13602805	77.6	12.9
13602807	36.5	5.3
13602811	0.8	8.2
13602813	64.5	77
13602814	73.5	14.7
13602816	72.3	15.6
13602818	76.8	38.5
13602819	75.7	9.3
13602824	13.1	1
13602828	24.2	1.8
13602830	52.8	13.6
13602831	56.5	15.2
13602834	72.8	16
13602835	50.6	4.4
13602836	30.4	11.6
13602837	43.5	5.6
13602839	26.4	10.8
13602840	65.2	20.4
13602841	17.1	9
13602842	54.8	4
13602843	29.1	20
13602850	29.0	14.3
13602852	25.9	3.2
13602854	14.1	77.1

13602855	0.2	12.9
13602857	60.3	142.4
13602860	0.4	25.2
13602863	0.5	100.6
13602864	0.4	71.7
13602866	0.4	101.8
13602867	69.9	28.5
13602868	61.9	95.5
13602870	8.1	9.3
13602873	8.9	25.3
13602874	10.2	4.6
13602880	6.7	4.5
13602881	15.5	1
13602882	60.5	22.9
13602888	68.3	13.8
13602893	2.0	6.7
13602894	0.3	19.5
13602895	1.1	13.8
13602898	0.6	14.5
13602902	5.0	1
13602903	41.6	187.5
13602907	18.2	1
13602910	18.3	1
13602914	54.0	16.5
13602917	23.3	1.6
13602918	38.4	11.2
13602922	60.3	8.9
13602924	29.6	9.2
13602927	59.9	16.4
13602928	16.3	1
13602930	11.0	1
13602936	62.3	2.9
13602937	26.4	19.3
13602939	12.7	1
13602940	13.1	10
13602943	9.1	115.4
13602944	50.7	11.2
13603025	45.5	92.2
13603028	2.7	1
13603029	5.4	3.1
13603030	34.8	2.4
13603037	40.2	109.8
13603038	47.8	7.4
13603128	1.0	1034.2
13603133	67.8	16.6
13603134	1.4	67.6
13603136	0.3	64.2
13603137	76.0	91.1
13603138	0.3	15.8
13603140	3.1	1
13603141	1.5	2.9
13603144	67.9	19.6
13603145	2.4	1
13603147	0.6	149.6
13603149	70.7	81.4
13603151	76.4	6.3
13603155	4.2	13

13603156	77.2	72.2
13603157	70.8	10.8
13603160	78.5	5.5
13603161	14.4	7.5
13603165	16.5	8.9
13603166	12.1	5.6
13603173	72.7	231.2
13603175	70.0	3.4
13603177	11.7	1
13603181	44.2	5.3
13603182	58.3	3.6
13603183	1.7	10.1
13603184	29.9	19.1
13603185	32.2	22.9
13603190	43.0	8.8
13603191	23.4	16.8
13603194	48.2	7.2
13603195	0.9	11.4
13603196	40.4	8.2
13603198	22.6	9.1
13603199	21.6	20.4
13603200	11.1	33.3
13603203	64.1	198.5
13603207	0.4	36
13603208	73.4	95.5
13603210	0.7	16.2
13603211	9.0	1
13603212	63.1	1
13603213	11.1	3.1
13603217	62.5	12.7
13603218	37.9	10.1
13603219	11.1	48.2
13603225	1.8	20.5
13603226	9.1	1
13603228	11.1	10
13603230	9.1	8.1
13603231	10.7	1
13603232	16.2	46.5
13603235	6.1	10.8
13603237	79.4	10
13603238	61.9	29.2
13603239	68.3	76.7
13603240	73.8	92.7
13603241	60.4	61
13603246	49.7	25.3
13603247	46.3	2.4
13603249	37.1	23.2
13603253	53.0	3.9
13603255	45.2	13.2
13603259	51.0	42.4
13603261	4.5	2.2
13603262	45.2	1.4
13603266	29.9	30.6
13603268	37.7	17.3
13603273	19.2	3.9
13603275	24.2	297.1
13603277	55.4	11.1

13603279	39.8	19.9
13603280	45.1	36.7
13603283	27.3	6
13603285	38.3	17.9
13603286	23.4	41.2
13603287	52.6	4.3
13603288	20.8	11.4
13603289	26.2	9
13603310	73.2	57.6
13603318	1.6	14
13603319	74.4	16.5
13603320	75.5	9.7
13603321	39.1	2.4
13603323	2.1	11.9
13603328	3.9	1
13603329	0.7	18.5
13603330	77.2	14.8
13603334	9.1	1
13603335	63.7	9
13603336	73.8	23.6
13603337	72.0	24.1
13603338	2.3	4.7
13603343	65.2	77.4
13603344	12.6	14.5
13603350	69.4	57.2
13603352	7.8	1.9
13603353	49.4	7.7
13603355	37.1	46.3
13603356	40.6	37.5
13603361	51.1	38.3
13603362	15.7	1
13603365	27.3	9.7
13603367	18.2	38.8
13603368	9.0	31.4
13603369	59.8	54.3
13603370	33.8	30.1
13603371	64.8	1.7
13603374	8.1	6.9
13603378	26.3	21.6
13603379	34.5	11.9
13603385	45.7	1.9
13603389	32.0	9.5
13603398	59.1	24.7
13603399	54.0	1
13603401	70.6	8.3
13603402	11.7	1
13603410	0.2	2.4
13603412	5.2	5.1
13603414	1.9	21.3
13603417	2.3	1
13603419	11.7	17.4
13603420	62.5	40.6
13603421	3.8	3.2
13603422	6.9	4.4
13603424	66.3	2.9
13603430	63.2	15.9
13603431	6.1	611.5

13603442	61.9	14.7
13603448	48.2	1.4
13603450	23.0	5.6
13603452	21.5	23.3
13603454	27.3	40.7
13603455	47.8	6.3
13603456	51.7	14.7
13603458	53.8	17.8
13603459	54.2	4.9
13603461	58.1	14.7
13603464	37.7	13
13603468	54.3	1
13603470	40.0	3
13603472	8.9	149.2
13603473	31.9	45.9
13603474	76.0	6.1
13603476	58.1	4
13603480	3.8	12
13603483	44.4	2.6
13603486	3.1	1
13603489	60.5	6.5
13603491	41.5	76
13603493	4.8	9.2
13603495	70.3	38.3
13603496	10.0	1
13603507	29.4	35.7
13603509	13.1	6.4
13603510	55.3	16.2
13603512	44.2	22.6
13603514	17.2	1
13603515	56.6	15.7
13603516	52.8	22.2
13603520	34.5	44
13603521	30.9	8.5
13603522	48.4	3.5
13603523	10.4	47.1
13603524	21.4	12.3
13603527	6.6	3
13603528	29.5	11
13603531	55.1	78.7
13603533	45.4	19
13603535	1.0	29.9
13603537	4.5	3.8
13603538	49.0	9.6
13603539	38.5	4.7
13603541	43.4	1
13603543	36.4	28
13603546	38.0	3.5
13603550	37.4	30.7
13603559	21.2	17.9
13603561	1.6	63.2
13603562	43.4	7.1
13603563	35.1	5.2
13603565	74.0	48.6
13603567	3.2	1
13603568	71.7	10.1
13603572	4.4	3.4

13603577	68.7	28.2
13603578	12.3	4.7
13603579	12.7	1
13603580	13.6	10
13603581	56.9	13.1
13603582	9.7	2.4
13603583	7.3	1
13603591	76.7	10
13603592	76.9	3.6
13603593	62.3	44.4
13603594	9.2	8.2
13603600	77.6	26.2
13603601	60.4	13.5
13603603	30.1	40.4
13603604	44.6	1.6
13603605	28.7	13.3
13603606	65.8	3.9
13603608	41.2	4.2
13603609	26.2	78.4
13603610	26.1	59.8
13603611	34.8	92
13603612	51.8	6.3
13603614	1.5	9.1
13603617	42.0	14.1
13603619	0.6	71.1
13603620	52.3	2.5
13603621	28.8	43
13603622	18.4	5.1
13603626	0.6	118
13603629	71.9	37.4
13603632	72.0	25.5
13603634	38.5	1.6
13603637	45.0	5.1
13603640	62.0	9.6
13603643	70.0	22.9
13603646	64.0	15.1
13603650	61.4	81.7
13603652	4.8	9.6
13603653	64.8	41.8
13603655	16.2	1.4
13603656	4.1	1169
13603657	4.9	6.1
13603658	15.6	1.7
13603659	12.9	1
13603660	12.5	9.5
13603661	79.8	75.8
13603665	52.8	3.6
13603666	76.0	25.4
13603667	32.9	5.5
13603670	50.0	63.6
13603676	35.4	10.5
13603677	3.1	1
13603678	22.8	4.9
13603680	21.5	21.2
13603682	33.6	2.5
13603685	44.3	4.4
13603686	41.2	12.6

13603689	45.2	44.4
13603696	54.3	2.1
13603697	24.7	1
13603704	62.3	56.1
13603706	50.4	52.9
13603707	56.0	4.1
13603712	6.3	1.8
13603713	66.7	105.3
13603714	9.6	5.9
13603715	4.9	1.5
13603716	69.0	43.8
13603717	6.7	1
13603719	2.4	1.1
13603729	74.2	60.7
13603733	6.4	5.6
13603734	67.4	25.3
13603738	11.6	4.2
13603741	71.3	12.1
13603742	9.8	21.7
13603744	71.8	24.3
13603746	72.2	1
13603750	2.2	4.4
13603756	37.8	16.6
13603758	28.7	4.6
13603759	43.9	19.9
13603762	39.6	67.9
13603763	59.0	122.1
13603765	27.2	85.9
13603767	55.4	33
13603770	13.0	22.4
13603773	0.8	8.9
13603775	33.7	26.2
13603777	47.9	30
13603778	19.4	2.7
13603780	23.3	20.1
13603782	26.3	3.2
13603784	4.5	1.1
13603788	27.6	376.3
13603789	65.3	9.2
13603790	37.6	29.8
13603792	14.6	4.8
13603794	29.2	50.8
13603795	21.9	1
13603797	7.7	2.5
13603798	77.6	33.8
13603799	44.7	4.1
13603802	0.6	207.2
13603807	55.4	5.2
13603808	72.6	133.2
13603809	61.4	2.8
13603811	1.9	15.5
13603813	3.7	1
13603814	2.1	23
13603816	0.4	32.2
13603817	62.6	61.2
13603823	79.1	43.9
13603824	67.2	49.2

13603828	13.5	4.6
13603832	15.6	23.5
13603835	15.3	15.1
13603837	27.9	9.1
13603841	56.5	1.1
13603843	22.3	112.4
13603850	20.5	5.3
13603851	44.0	6.2
13603854	43.5	23.1
13603858	0.7	13.8
13603859	17.9	1
13603862	67.5	17.9
13603868	1.8	14.5
13603869	25.8	123.7
13603871	39.4	1.5
13603872	24.3	5
13603873	13.4	1
13603874	7.6	11.6
13603877	75.6	1.6
13603880	72.5	18.2
13603881	6.5	5.7
13603882	5.2	9.4
13603883	8.7	4.5
13603884	8.4	14.9
13603885	71.5	34.9
13603887	10.3	17.7
13603888	75.6	11.7
13603889	64.3	27.2
13603891	8.1	1.2
13603893	8.6	5.3
13603894	77.8	37.6
13603898	11.7	6
13603903	37.2	9.3
13603904	73.4	19.9
13603905	17.3	1.3
13603912	67.9	36.9
13603914	43.0	13.3
13603916	66.5	32.6
13603918	31.7	33.1
13603920	43.7	4.3
13603921	42.5	10
13603923	13.8	24.6
13603927	26.1	20.6
13603929	57.6	1
13603935	77.5	5.6
13603936	70.9	4.3
13603937	54.8	20.3
13603938	18.5	95.6
13603939	62.4	26.2
13603941	1.6	38.3
13603945	2.9	3.3
13603946	35.6	14.7
13603948	0.4	66.3
13603949	3.2	3.2
13603950	12.6	125.6
13603951	57.3	11.8
13603952	1.9	8.5

13603962	53.9	6.6
13604011	61.3	21.1
13604039	66.9	71.1
13604088	13.4	4.7
13604125	76.4	40.5
13604147	75.0	9.3
13604150	53.4	17.2
13604178	66.9	27.4
13604183	11.9	36.6
13604185	56.7	35.7
13604189	15.4	1
13604193	26.0	3.2
13604197	2.4	2
13604201	76.4	31.5
13604210	13.8	33.7
13604245	45.2	3.3
13604248	10.0	189.3
13604256	1.8	11.2
13604260	9.4	1.4
13604278	5.9	31.5
13604298	60.8	32.1
13604312	57.1	5.6
13604313	24.2	3.2
13604314	60.8	160.2
13604316	46.8	16.5
13604324	15.3	11.6
13604327	9.0	1.4
13604328	28.9	10.9
13604329	66.3	24.8
13604368	21.2	32.1
13604373	66.8	14.7
13604377	79.9	1.1
13604378	78.4	38.9
13604379	0.9	10.2
13604380	61.7	11.5
13604384	78.0	51.9
13604385	75.9	15.3
13604387	4.8	22.9
13604394	49.6	2.2
13604397	54.4	60.5
13604402	54.1	1
13604404	56.7	2.2
13604405	45.2	18.6
13604407	38.1	14.4
13604409	10.6	70
13604410	48.7	36.3
13604413	38.6	26.9
13604416	37.1	44.6
13604419	8.9	3
13604424	17.7	7.7
13604425	53.1	52.5
13604429	38.8	2.3
13604430	62.4	8
13604431	43.2	3
13604432	57.7	4.9
13604433	40.7	13.2
13604435	72.7	4.6

13604438	54.7	7.5
13604443	47.5	18.3
13604444	5.8	1
13604448	73.9	1
13604449	11.0	62.9
13604457	6.0	14.1
13604459	17.8	628.5
13604460	75.0	10.5
13604462	15.2	2.5
13604463	13.1	3.3
13604464	65.4	34.4
13604465	14.9	1.4
13604466	71.4	8.7
13604467	62.2	28.9
13604468	69.4	42.5
13604469	61.6	20
13604472	67.9	177.1
13604474	34.2	8.1
13604476	4.0	15.2
13604481	13.7	1
13604485	37.3	11.3
13604487	21.5	16.2
13604491	38.0	10.8
13604492	46.1	5.5
13604493	29.9	16.2
13604494	26.2	8.6
13604497	57.4	64.1
13604499	35.2	18.4
13604503	12.3	1.3
13604507	73.2	4.9
13604508	79.8	3.2
13604513	57.5	7.9
13604514	66.7	38.1
13604516	62.4	21.9
13604519	70.5	50.2
13604524	74.5	17.7
13604526	44.0	101.6
13604527	4.5	3.2
13604529	3.6	49.2
13604531	73.8	15.2
13604534	5.0	1.2
13604537	67.1	37.8
13604540	53.9	21.9
13604543	4.4	6.6
13604546	4.9	15
13604548	51.6	1.7
13604549	9.8	5.1
13604550	76.2	20.6
13604554	37.1	26.4
13604555	47.8	34.6
13604556	0.4	28.5
13604558	27.2	5.6
13604563	13.4	38.2
13604564	40.9	3.3
13604565	22.2	7.7
13604567	42.9	17.2
13604568	10.6	1

13604570	0.7	34
13604571	59.2	24.7
13604572	0.3	3
13604576	63.8	19.9
13604578	76.0	26.3
13604580	9.8	1
13604581	60.9	11.8
13604582	0.3	24.6
13604583	45.7	61.3
13604585	31.7	1
13604588	47.5	14
13604589	38.0	14.7
13604590	22.3	43.8
13604596	77.3	8.4
13604599	6.9	1.5
13604600	62.7	4
13604601	9.8	1
13604603	6.1	1
13604604	3.8	1
13604605	11.4	1
13604606	0.8	88.3
13604608	7.6	1.8
13604609	61.7	2.9
13604610	24.7	39.7
13604612	69.6	125.2
13604615	74.7	55.3
13604618	73.6	13.4
13604621	47.9	21.7
13604622	40.3	22.4
13604625	26.4	2.5
13604626	32.0	2.8
13604630	1.8	15.1
13604631	23.0	15.1
13604633	20.6	32.8
13604634	47.5	13.7
13604635	51.9	14.9
13604636	35.0	1.9
13604637	34.4	23.6
13604638	36.3	294.4
13604639	65.9	3.9
13604640	49.1	10.1
13604641	32.6	10.9
13604642	17.2	1.3
13604643	2.9	4.7
13604644	56.0	17.8
13604645	33.3	45.3
13604646	39.6	14.8
13604648	14.5	11.4
13604650	47.8	56.2
13604653	33.9	7.1
13604654	47.9	15.2
13604657	50.9	13.5
13604659	0.6	59
13604662	0.2	1
13604664	74.3	10.2
13604666	1.7	163.4
13604668	61.7	17.2

13604671	1.6	4.8
13604673	71.4	2.3
13604675	75.6	21.1
13604677	12.1	1.8
13604685	66.7	13.5
13604692	1.4	80.2
13604695	57.6	10.5
13604698	29.1	16.1
13604699	55.8	1.6
13604700	70.4	7
13604701	18.4	7.9
13604702	4.5	18.9
13604704	7.5	1.2
13604705	39.7	29.9
13604706	45.0	5.4
13604708	31.1	4
13604710	38.2	2.5
13604711	69.9	57.3
13604714	48.8	13.6
13604718	51.8	1
13604719	28.7	1.5
13604720	38.9	1
13604727	10.6	3.2
13604729	40.2	9
13604730	67.4	11.9
13604732	39.6	63.6
13604735	29.7	38.4
13604736	21.0	3.8
13604737	26.3	1
13604738	0.8	7.1
13604739	22.2	14.8
13604742	8.8	2.3
13604745	11.4	25.1
13604752	61.7	20.2
13604753	43.7	16.7
13604757	4.1	64.3
13604758	33.5	68.9
13604763	3.0	4.5
13604768	9.5	1
13604775	2.6	1.5
13604776	7.4	6.3
13604777	6.5	9
13604780	22.8	2.6
13604785	33.6	5.7
13604789	20.3	1.2
13604790	31.5	31.1
13604792	35.8	37.9
13604795	55.8	12.6
13604796	52.4	7.3
13604797	44.3	8.8
13604801	13.5	5.7
13604805	75.9	35.5
13604808	70.6	5.6
13604810	40.5	1
13604811	0.8	6.2
13604812	70.1	198.9
13604814	0.7	26.1

13604816	3.4	1
13604818	3.9	1
13604819	0.6	14.6
13604822	62.4	4.5
13604824	13.8	123.2
13604826	76.8	56.2
13604827	15.7	16.7
13604837	53.0	21.1
13604839	49.9	10.1
13604843	59.4	31
13604845	27.5	15.8
13604847	28.0	3.2
13604848	52.7	44.5
13604854	43.1	12.1
13604855	77.2	17.6
13604856	50.9	26.8
13604857	47.7	4.6
13604858	41.6	8.5
13604859	38.4	33.9
13604860	7.8	9.1
13604861	26.4	7.9
13604863	15.5	67.9
13604864	58.6	2.5
13604867	71.3	140.4
13604868	55.9	39.9
13604870	12.6	1
13604872	33.5	18
13604874	30.9	4.2
13604875	45.7	52.5
13604876	36.0	15.2
13604877	73.2	9.9
13604882	48.5	1.6
13604883	17.2	1.7
13604885	22.4	91.5
13604887	2.4	1.9
13604889	43.9	1.3
13604890	38.6	64.9
13604894	54.6	101
13604895	32.0	1
13604897	26.5	3
13604898	38.8	29.6
13604899	52.3	9.1
13604901	74.2	27.4
13604914	62.2	12.6
13604915	76.0	2.8
13604918	37.9	4.4
13604920	6.4	4.8
13604924	14.9	4.7
13604926	0.7	30.2
13604928	66.1	8.6
13604932	20.5	1
13604934	15.6	36.1
13604937	24.3	13
13604940	20.5	10.4
13604946	36.5	1
13604947	34.8	7.1
13604948	28.8	23

13604952	29.0	6.4
13604954	22.4	1
13604957	27.6	1
13604958	37.1	5.1
13604960	51.8	9.2
13604961	62.0	3.7
13604962	36.9	26
13604964	41.2	12.6
13604965	36.4	18
13604966	52.8	12.9
13604968	60.6	4.9
13604970	30.8	8.2
13604973	39.1	4.6
13604974	63.7	20.6
13604977	31.1	53.2
13604984	36.9	16.1
13604985	53.2	1
13604986	32.7	23.7
13604988	16.7	28.7
13604990	74.5	20.3
13604992	5.8	1
13604995	29.3	37.9
13604997	36.9	1
13604999	42.9	24.2
13605000	73.0	4.4
13605001	73.0	12.7
13605009	74.7	5.3
13605012	72.2	10.6
13605015	70.7	22.8
13605017	1.9	1.3
13605018	56.2	61.3
13605020	66.7	132.5
13605022	74.5	129.1
13605024	77.5	120.7
13605027	77.4	7.4
13605032	68.5	59.5
13605035	67.6	23.1
13605047	8.0	23.7
13605048	10.5	24.2
13605053	7.1	1.5
13605057	15.1	51
13605058	0.9	23.1
13605060	5.6	21.7
13605062	11.8	4.1
13605070	65.8	7.8
13605071	58.4	1
13605072	45.1	80.8
13605073	16.9	1
13605076	40.0	15.4
13605077	45.5	9.8
13605082	22.4	7.6
13605084	4.5	4.4
13605086	26.1	10.2
13605089	24.2	8.8
13605091	33.1	3
13605092	17.7	8.9
13605093	26.8	6.5

13605094	60.2	13.2
13605096	14.6	5
13605098	55.6	17.1
13605100	52.0	21.3
13605102	59.1	32.9
13605104	36.0	1.8
13605106	71.6	28.5
13605108	75.7	10.3
13605110	0.5	25.1
13605114	75.1	12.3
13605117	9.2	47.6
13605118	7.9	11.1
13605120	51.3	20.6
13605124	3.0	5.2
13605135	7.7	192.4
13605136	23.4	1
13605137	3.8	1
13605138	76.4	204.8
13605142	6.6	2.1
13605143	5.6	1
13605147	20.7	2.8
13605148	52.7	33
13605149	73.5	1.1
13605151	59.7	3.1
13605153	73.0	48.9
13605154	73.8	26.1
13605155	1.0	352.4
13605157	56.4	102.9
13605159	56.7	1
13605161	0.3	55.1
13605163	73.3	12.7
13605167	48.2	39
13605168	57.8	12
13605171	26.7	10
13605175	19.2	36.8
13605176	22.2	12.8
13605177	34.4	92
13605181	36.4	46.3
13605183	22.9	14.5
13605187	33.5	6.6
13605192	39.7	53.7
13605194	24.4	1.9
13605195	18.3	3.2
13605197	19.6	11.9
13605199	65.9	6.2
13605210	64.9	21
13605213	77.6	3.8
13605215	2.6	1
13605216	68.5	168.3
13605217	2.9	1.1
13605226	61.0	6
13605228	10.3	23.8
13605229	11.6	1
13605230	4.7	135.5
13605233	4.4	55.5
13605234	64.8	31.9
13605241	17.0	10.8

13605242	14.0	3
13605244	62.9	34.9
13605246	27.4	9.2
13605247	47.3	1
13605249	44.1	25.4
13605251	38.8	2
13605252	51.8	191.2
13605253	3.2	1
13605256	22.8	16.8
13605257	56.4	26.3
13605263	0.3	9.5
13605265	20.8	24.2
13605269	17.0	3.7
13605272	48.1	8.2
13605273	32.0	96.9
13605274	8.4	5.4
13605275	34.7	48.6
13605279	21.3	13.6
13605284	73.8	18.9
13605287	11.4	1
13605291	0.2	8.4
13605292	0.3	9.7
13605294	74.6	7.8
13605301	4.4	20.5
13605304	4.9	1.9
13605306	71.3	27.7
13605309	15.0	339.8
13605311	48.6	84.6
13605313	0.2	1
13605314	33.7	71.1
13605319	39.5	100.2
13605320	48.2	7.1
13605323	12.2	1.6
13605325	32.5	1.2
13605326	57.0	42.7
13605328	20.3	1.9
13605335	35.7	3.4
13605336	58.6	17.6
13605338	73.1	1
13605343	12.4	9.1
13605344	67.2	66
13605345	0.9	8.8
13605346	1.4	134.9
13605352	6.8	195.7
13605353	58.1	65.2
13605358	28.1	48.5
13605359	52.0	3.1
13605368	71.2	42.9
13605371	0.3	5.2
13605373	37.4	44.2
13605375	73.9	23.4
13605376	0.4	126
13605378	0.7	10.8
13605381	75.0	12.7
13605385	3.3	1
13605386	3.0	1
13605387	1.0	63.6

13605389	79.8	3.6
13605391	74.0	1.5
13605392	75.8	6.7
13605393	15.7	34.9
13605394	64.4	19.6
13605395	62.1	1.4
13605396	62.2	33.5
13605398	4.9	23.3
13605403	61.6	41.1
13605404	14.4	3.4
13605405	8.0	9
13605406	41.9	6
13605408	16.1	25.3
13605410	64.4	98.4
13605412	35.4	25.8
13605416	36.9	10
13605419	50.3	5
13605424	38.1	3.3
13605425	34.5	219.6
13605427	69.2	11.7
13605429	37.0	8.2
13605430	60.1	6.9
13605432	47.0	1.1
13605437	8.9	28.3
13605439	64.8	3.4
13605442	74.5	8.5
13605444	29.4	3.7
13605446	78.1	12.5
13605457	35.7	16
13605459	66.9	3
13605462	56.7	5.6
13605463	53.3	53.2
13605465	0.3	1
13605473	38.4	32.4
13605474	2.4	9
13605475	34.5	3.6
13605476	2.8	1.1
13605477	0.6	20.3
13605479	70.9	33.7
13605480	25.8	2.6
13605482	76.7	46.1
13605483	15.5	21.2
13605484	79.0	4.9
13605489	4.3	1
13605494	12.9	1.5
13605495	11.0	1
13605498	68.1	1
13605502	5.3	2.8
13605505	8.9	18.2
13605507	6.1	1.3
13605509	12.0	2.2
13605510	5.3	1
13605511	8.1	1.3
13605513	9.8	3.8
13605514	77.8	54.8
13605516	13.6	1.2
13605517	65.5	17.4

13605518	43.3	51.2
13605519	56.5	10.2
13605520	18.7	1
13605521	37.7	1.2
13605530	32.4	19.7
13605533	58.2	2.1
13605534	33.7	2.4
13605536	30.3	5
13605537	32.0	1.1
13605540	36.8	7.9
13605542	74.5	25.9
13605544	75.1	59
13605548	46.8	23.5
13605551	0.7	18.9
13605553	0.7	17.4
13605554	72.6	3.3
13605558	60.9	3.1
13605559	62.6	13.2
13605561	77.0	3.8
13605563	77.5	63.5
13605564	8.9	17.4
13605568	15.0	38.1
13605569	3.4	1
13605572	4.6	1.3
13605573	4.1	1.2
13605574	37.2	15.2
13605577	60.9	140.9
13605578	65.8	10.8
13605583	13.0	31.3
13605588	75.0	18.3
13605589	40.4	1
13605590	0.9	26.3
13605593	60.0	18.8
13605595	39.7	1.3
13605596	39.6	11
13605597	60.9	29.7
13605598	43.5	18.1
13605599	56.8	37.8
13605602	19.5	4.7
13605607	4.6	40.5
13605608	33.9	13.5
13605609	40.8	4.3
13605611	34.0	7
13605612	35.1	6.6
13605615	32.0	7.8
13605616	29.7	5.3
13605617	24.0	38.9
13605620	27.6	1
13605623	55.0	16
13605627	53.0	4
13605628	20.0	1
13605630	13.4	1.6
13605631	69.2	4.3
13605632	6.3	11.9
13605634	60.7	5.3
13605635	0.9	6.5
13605636	66.3	2.5

13605640	9.8	6.3
13605642	9.3	4.3
13605644	62.6	11.5
13605645	63.2	19.9
13605646	6.7	1.1
13605648	57.7	17.9
13605652	67.9	5.1
13605653	0.8	28.5
13605654	1.0	584.5
13605655	4.0	50.6
13605656	77.3	11.5
13605657	30.2	6.8
13605658	9.5	1.1
13605659	11.0	50
13605660	5.4	3.9
13605662	69.7	14.5
13605663	2.1	8.5
13605665	21.4	7.7
13605675	54.4	1
13605676	31.6	60.8
13605677	35.6	3.1
13605681	28.1	7.4
13605683	55.5	1.9
13605684	51.6	19.8
13605689	21.5	2.3
13605694	41.8	3.3
13605695	73.0	52.1
13605696	71.8	11.1
13605698	53.4	4.9
13605699	1.0	185.4
13605703	40.9	4.8
13605704	30.4	74.5
13605707	38.2	28.7
13605708	0.5	139.6
13605710	25.3	22.9
13605712	0.6	12.9
13605713	3.4	1
13605714	51.1	168
13605715	15.9	1.1
13605716	26.4	14.4
13605718	66.2	40.3
13605727	2.1	2.6
13605730	35.0	32.4
13605733	71.7	29.3
13605736	62.9	22.3
13605737	60.6	15.7
13605747	13.8	48
13605750	10.7	1
13605751	39.3	14.9
13605760	11.1	14.6
13605761	7.8	43.6
13605764	19.3	2.2
13605768	47.0	3.8
13605770	35.8	55.7
13605779	33.2	68.9
13605781	24.3	1
13605791	33.2	20.3

13605793	54.1	16.8
13605794	71.8	115.8
13605795	32.5	12.2
13605796	1.7	15.9
13605799	36.8	114.5
13605801	51.8	1.9
13605802	38.8	5.1
13605803	44.7	35.8
13605804	27.7	12.6
13605806	27.0	86.7
13605807	38.4	25.4
13605808	58.1	36.9
13605814	41.1	87
13605818	63.5	24
13605820	36.1	16.2
13605835	17.9	1
13605848	4.6	28.8
13605851	57.5	19.5
13605854	69.1	29.1
13605859	6.9	522.2
13605866	6.5	2.6
13605871	2.4	31.5
13605874	3.7	1
13605876	58.9	7.4
13605877	56.1	12
13605886	76.2	3.2
13605892	72.9	9.4
13605893	3.1	14.2
13605894	76.8	19.9
13605900	41.5	3.2
13605902	37.5	4.4
13605905	45.7	3.8
13605910	31.1	5.9
13605914	17.7	17.8
13605925	35.1	44.1
13605927	59.7	7.1
13605930	62.4	6
13605931	42.7	7
13605944	7.4	8.1
13605948	60.9	11.2
13605955	68.9	1.2
13605961	58.2	16.5
13605965	42.2	138.4
13605967	53.2	24.8
13605973	40.8	23.8
13605977	4.3	10.8
13605982	5.4	7.1
13605988	75.9	63.4
13605991	70.8	59.2
13605996	20.8	19.5
13605997	70.1	23.1
13606005	40.2	2.7
13606014	51.8	15.4
13606018	28.8	10.8
13606019	32.3	57.3
13606020	35.2	42.7
13606027	62.7	160.7

13606030	70.7	20
13606032	39.0	14.1
13606034	64.1	27.5
13606037	71.9	1.2
13606040	65.3	5.8
13606041	65.9	3.2
13606042	67.9	22.8
13606044	70.0	37.3
13606046	61.3	64.9
13606049	73.6	17.5
13606051	0.3	117.7
13606054	72.1	93.3
13606057	12.2	27.1
13606061	34.5	9.3
13606064	13.4	17.8
13606066	37.4	6.2
13606067	37.4	6.2
13606068	21.6	33.9
13606071	29.7	51.3
13606072	27.9	7.5
13606073	21.2	1.2
13606079	20.9	70.6
13606082	16.9	12.5
13606084	24.8	17.4
13606086	23.7	1
13606087	49.0	18.2
13606092	60.3	4.4
13606101	13.4	1
13606103	27.2	6.9
13606104	45.2	48.3
13606105	7.6	16.5
13606110	7.6	54.7
13606112	45.1	13.4
13606113	23.4	8.5
13606116	25.2	36.6
13606118	16.5	11.3
13606119	56.2	21.8
13606120	47.1	7.4
13606124	36.9	4
13606125	74.4	108.6
13606126	65.8	10
13606127	10.7	6.2
13606134	7.7	22.6
13606136	58.5	133.4
13606137	48.4	40.5
13606138	52.3	31.9
13606139	62.5	52.3
13606142	2.9	2.7
13606143	2.9	2.8
13606144	14.6	13.3
13606146	3.6	4.1
13606148	7.6	13.2
13606152	11.9	11.4
13606154	61.0	6.6
13606156	1.3	47.4
13606157	0.9	33.2
13606158	15.1	1

13606164	7.6	7.5
13606169	37.2	13.3
13606170	47.8	19.9
13606171	61.0	8.6
13606172	41.3	9.3
13606174	56.9	67.1
13606178	34.0	17.7
13606182	42.7	63.7
13606186	2.5	2.6
13606190	54.3	8.7
13606192	36.5	12.9
13606193	32.3	31.8
13606194	39.4	8.5
13606195	37.9	1.4
13606196	28.8	15.1
13606201	3.0	2.1
13606204	23.2	23.4
13606205	29.7	28.2
13606207	39.3	9.1
13606217	20.0	2.2
13606225	34.1	4.6
13606240	2.1	53.3
13606242	33.9	23.9
13606245	79.3	17.5
13606248	57.6	19.2
13606249	0.7	20.8
13606250	59.4	2.7
13606256	12.9	56
13606258	64.6	40.9
13606259	70.5	3.3
13606261	7.2	2.1
13606262	64.7	26.2
13606263	14.1	145
13606265	16.6	4.7
13606267	23.4	1
13606268	41.6	12.5
13606270	19.8	325.6
13606271	24.3	1
13606272	43.4	2.2
13606274	21.8	65.7
13606275	21.3	19
13606279	29.8	3.1
13606282	55.2	28.1
13606283	58.9	98.6
13606285	31.0	3.8
13606286	39.8	1
13606287	52.6	25.2
13606292	36.4	14.9
13606293	39.9	1.4
13606295	43.5	6.7
13606298	43.1	9.8
13606299	9.1	5.1
13606300	49.5	10
13606301	12.2	18.3
13606302	7.7	2.4
13606306	0.2	18.7
13606311	47.4	21.5

13606315	0.4	41.5
13606316	17.1	4.4
13606317	52.3	7.2
13606318	33.7	11.8
13606320	43.3	42.1
13606321	34.4	9.1
13606322	36.5	22.2
13606329	0.2	2.7
13606330	66.7	51.3
13606333	2.3	5.8
13606334	14.7	34.3
13606336	66.6	3.2
13606337	58.0	10.5
13606339	54.4	1
13606344	64.6	10
13606348	7.6	12.4
13606350	39.6	4.7
13606351	78.5	1.8
13606352	1.2	123
13606353	9.3	1
13606355	10.5	1.9
13606356	9.4	1
13606361	65.0	7.3
13606362	69.3	6.7
13606363	79.7	24.6
13606364	42.3	3
13606369	27.6	27.6
13606370	60.7	16.4
13606372	41.0	8.3
13606374	20.6	1
13606375	43.8	31.3
13606376	37.9	1
13606378	58.6	68.4
13606380	38.7	8.3
13606382	46.3	25.2
13606384	48.5	21.2
13606385	21.9	41
13606392	35.2	114.1
13606393	31.9	1.9
13606396	53.3	19
13606401	72.1	10.9
13606402	72.5	12.7
13606404	75.2	4.3
13606405	3.4	1
13606406	6.4	5.3
13606409	74.9	2.9
13606411	63.9	29.4
13606413	72.5	2.4
13606416	75.3	20.1
13606420	16.6	1
13606421	0.9	10.1
13606422	64.5	42
13606424	16.3	8.3
13606427	14.1	31
13606432	19.3	130.6
13606434	46.1	2.3
13606435	33.9	3.2

13606436	21.2	5
13606437	27.5	1
13606442	14.5	1.1
13606444	41.7	75.5
13606449	0.8	22.3
13606450	49.5	1.1
13606453	44.3	57
13606456	55.6	15.6
13606457	35.1	16.2
13606459	29.6	65
13606461	45.3	13.9
13606464	24.8	15.7
13606467	34.8	19.1
13606469	23.6	7.2
13606470	31.6	11.8
13606473	39.6	5.5
13606477	0.1	70.4
13606478	59.3	21.2
13606481	12.3	1
13606483	8.6	1
13606485	31.3	33.2
13606488	35.6	36.5
13606489	55.9	43.7
13606491	77.0	235.7
13606492	36.8	22.4
13606494	28.3	6.5
13606497	36.8	45.1
13606498	11.8	13.9
13606499	25.9	49
13606501	58.8	30.7
13606502	0.6	17.9
13606503	29.9	218.8
13606504	32.9	786.4
13606506	20.5	13.3
13606509	69.1	32.6
13606513	75.8	34.6
13606516	60.4	47.3
13606518	65.7	68.9
13606519	0.3	21.2
13606531	5.6	6.3
13606534	49.6	3.2
13606536	66.9	2.4
13606539	43.6	23.1
13606540	75.7	46.7
13606542	7.5	3
13606544	15.3	6.8
13606546	75.7	18.7
13606547	72.6	22.8
13606548	10.8	1
13606549	51.4	22.4
13606552	62.8	1
13606556	1.1	192.6
13606557	37.4	61.9
13606559	52.8	242.7
13606562	22.3	25.6
13606563	46.5	3.6
13606566	44.4	7

13606567	48.9	5.4
13606571	54.6	34.8
13606575	57.3	10.9
13606579	5.7	2.7
13606582	19.5	2.4
13606584	28.5	40.9
13606586	34.4	1.9
13606587	42.1	59.4
13606588	20.3	32.2
13606590	6.7	51.9
13606591	41.0	6
13606592	28.1	2.9
13606594	75.8	1
13606596	0.4	35.5
13606597	70.0	8.5
13606598	63.6	10.9
13606599	70.4	1.5
13606600	8.6	31
13606603	65.9	10.3
13606605	32.7	2.4
13606607	71.6	5.8
13606608	68.2	11.6
13606611	69.4	33.3
13606612	4.3	6.6
13606613	61.3	48.2
13606614	4.7	2.4
13606616	7.4	11.3
13606619	2.3	228.3
13606621	44.0	18.7
13606622	15.3	3.5
13606628	37.2	5.1
13606631	26.6	15.4
13606634	38.5	27.7
13606636	26.6	4.9
13606643	79.8	47
13606644	62.4	13.2
13606647	60.7	6.7
13606648	2.1	5.6
13606649	16.4	1
13606650	3.8	1
13606652	3.6	1
13606653	63.4	10.9
13606658	48.3	20.5
13606660	49.0	8.2
13606661	0.2	4.7
13606663	52.3	133
13606664	50.0	7.7
13606665	50.5	13.5
13606666	0.7	22.4
13606669	33.8	18.3
13606673	57.0	8.9
13606675	16.3	1
13606678	20.2	195.1
13606683	18.4	27.8
13606684	51.6	44.1
13606686	20.6	4.4
13606688	6.9	5.7

13606689	27.8	2.4
13606690	4.9	25.7
13606699	60.1	20.2
13606704	70.5	39.8
13606706	60.7	7.1
13606708	71.8	5.3
13606713	70.1	10.4
13606714	2.1	9.9
13606715	41.6	4.9
13606717	22.6	15.4
13606718	63.9	45
13606719	72.1	10.4
13606725	12.6	1.3
13606726	69.0	46.2
13606731	15.0	3.8
13606732	45.1	46.4
13606733	13.4	38.7
13606735	31.4	2.7
13606737	49.7	25.3
13606738	55.9	57.3
13606741	25.2	1
13606742	27.4	4
13606744	37.5	1
13606747	52.0	12
13606748	66.0	13.1
13606749	34.6	120.2
13606756	14.3	2.3
13606761	57.4	4.3
13606763	13.3	3
13606766	38.4	7.6
13606769	53.0	8.5
13606772	64.1	20.4
13606773	69.2	107.4
13606774	56.1	9.8
13606775	25.4	25.6
13606781	35.9	2.6
13606784	4.6	1.9
13606787	1.7	114.9
13606788	37.0	34.3
13606791	20.0	2.3
13606792	21.1	1.7
13606794	21.5	2.6
13606795	7.6	3.2
13606797	13.5	7.9
13606800	41.9	11.1
13606802	4.7	29
13606805	9.5	1
13606807	65.6	15
13606808	36.5	1.2
13606809	77.6	4.3
13606810	69.7	107.5
13606812	71.9	33.8
13606813	71.8	13.8
13606814	69.7	10.1
13606818	32.4	1.2
13606823	21.8	2.3
13606826	41.5	11.5

13606828	49.3	92
13606829	18.3	2.5
13606832	45.4	5.7
13606835	59.6	2.7
13606837	33.8	5
13606838	35.1	5.6
13606844	21.2	3
13606845	59.7	60
13606847	30.7	11
13606850	46.6	23.6
13606851	67.5	4.7
13606855	20.7	23.6
13606860	53.4	3.6
13606862	32.8	11.7
13606863	33.1	114.9
13606865	27.0	6.7
13606866	65.3	62.2
13606868	1.2	93.7
13606871	78.8	18.2
13606873	74.9	51.7
13606874	68.2	12.3
13606875	60.7	10.7
13606876	6.0	4.3
13606878	69.7	77.5
13606881	3.9	95
13606882	0.3	26
13606883	59.3	2.4
13606884	65.2	8
13606886	79.4	10.8
13606887	48.0	75.4
13606888	32.8	71.4
13606889	42.1	10.6
13606892	0.4	29
13606893	24.6	1
13606894	51.9	3.1
13606897	35.2	12.5
13606898	75.9	99
13606899	78.6	41.3
13606901	72.0	7.9
13606905	63.0	4.7
13606906	71.6	12.4
13606910	61.6	6.9
13606911	30.2	17.4
13606914	76.8	24.7
13606915	71.3	12.5
13606916	55.4	191.2
13606918	32.4	50.3
13606919	2.8	1.1
13606922	23.3	2
13606924	29.6	28
13606925	50.7	5.2
13606929	42.9	1.1
13606930	60.0	12.5
13606931	53.2	15
13606932	18.0	10.4
13606933	75.0	12.5
13606935	25.3	4.6

13606936	27.2	7.6
13606938	12.7	49.2
13606940	1.0	8.2
13606941	1.1	115.2
13606944	62.2	1.2
13606946	4.7	4.6
13606954	12.4	1
13606955	2.3	13.2
13606960	76.8	40.3
13606964	70.8	1
13606966	0.5	20.9
13606969	4.8	1.5
13606972	0.8	1
13606973	64.0	2.9
13606976	25.4	4.3
13606977	26.0	35.4
13606978	1.0	120.7
13606979	56.2	4.2
13606983	35.8	32.1
13606985	59.0	54.6
13606988	21.2	4.7
13606989	57.9	174.6
13606991	20.9	19.1
13606993	26.2	39.7
13606996	29.5	35.7
13607000	39.1	14
13607001	45.8	10.5
13607110	2.5	3.6
13607119	27.9	1.4
13607123	59.1	1.5
13607124	70.1	139.6
13607125	38.2	13.4
13607126	2.0	28.5
13607130	23.5	102
13607136	42.5	17.2
13607295	61.1	45.2
13607297	67.9	25.7
13607298	0.9	9.1
13607300	1.3	61.8
13607301	66.4	32
13607303	0.2	2.7
13607304	0.5	22.2
13607305	45.0	6.4
13607306	5.4	1.1
13607307	0.2	1
13607308	69.0	10.7
13607309	0.2	2.2
13607311	0.8	9.5
13607312	52.3	22.5
13607315	2.4	26
13607317	68.7	23.8
13607319	0.6	14.8
13607322	12.2	1
13607324	31.3	4
13607327	0.5	71.5
13607328	11.4	61.2
13607329	7.0	20.6

13607331	9.2	5
13607332	6.3	1
13607335	60.6	25.8
13607338	8.3	88.6
13607339	10.3	13.9
13607341	71.6	8.6
13607344	62.7	8.9
13607345	79.4	41.7
13607347	11.1	1
13607348	8.6	22.5
13607351	32.3	5.6
13607352	0.8	20.4
13607355	2.1	21.1
13607357	67.2	14.2
13607362	27.5	10.2
13607367	32.9	1
13607369	39.1	33.9
13607370	54.7	83.9
13607376	0.5	111.2
13607378	3.7	1
13607379	38.4	4.4
13607381	37.7	26.8
13607382	13.8	16.6
13607383	39.9	4.1
13607384	46.8	12.1
13607419	54.2	69.3
13607422	67.7	4.6
13607424	0.3	18.8
13607425	15.9	17.2
13607428	0.7	57.5
13607429	55.8	61
13607434	3.4	148.5
13607444	72.8	1
13607450	70.6	1
13607454	12.6	7.2
13607458	1.8	16.1
13607465	1.5	53.1
13607469	33.1	40.4
13607470	0.7	27.4
13607480	31.8	8.9
13607482	25.7	4.4
13607486	36.6	17.9
13607487	20.3	1.4
13607490	34.3	2.7
13607491	28.2	4.7
13607706	65.7	5.9
13607707	77.4	40.9
13607711	22.6	40.4
13607712	45.3	2.9
13607717	78.5	49.5
13607721	27.4	89.4
13607722	13.1	5.1
13607723	35.0	4.6
13607812	0.7	56.2
13607815	67.8	16
13607823	4.7	40.2
13607824	54.4	16.7

13607826	0.3	8.6
13607829	78.1	13
13607833	41.0	5.7
13607835	66.9	12.6
13607836	75.5	19.4
13607837	5.6	231
13607838	5.1	236.1
13607840	0.5	161.6
13607845	68.4	3.6
13607846	67.8	19.6
13607847	62.3	1.7
13607851	14.2	107.5
13607856	16.9	95.3
13607858	4.6	8.6
13607859	16.9	25.7
13607860	13.7	11.4
13607864	16.8	20.8
13607867	16.5	2
13607868	61.4	15.8
13607869	13.0	21.6
13607870	72.0	4.7
13607872	14.9	13.7
13607875	73.8	21.7
13607876	64.7	28.9
13607878	16.0	1
13607881	12.6	9.1
13607882	60.9	9.2
13607883	39.3	29.2
13607884	0.5	110.7
13607886	48.9	9.7
13607890	32.0	95.7
13607893	30.3	44.7
13607894	70.7	4.5
13607895	21.4	4.6
13607896	23.4	7.1
13607898	0.8	20.1
13607900	47.0	141.2
13607902	7.0	1.4
13607903	45.6	8.1
13607904	49.2	14.3
13607905	58.1	52.3
13607907	29.5	17.5
13607908	3.6	34.6
13607910	55.0	6.5
13607911	71.0	21.3
13607912	35.4	2.7
13607913	35.4	6.5
13607914	3.7	1
13607919	66.1	5.2
13607921	19.3	5.5
13607923	33.4	25.1
13607924	0.2	17.4
13607925	73.8	9.5
13607933	2.8	1.8
13607941	45.4	2.6
13607942	0.1	22.7
13607944	0.3	18.9

13607947	69.2	25.2
13607948	16.1	2.9
13607950	79.8	21.3
13607951	19.9	15
13607952	0.5	149
13607955	73.8	114.4
13607956	68.1	23
13607957	0.7	84.6
13607958	11.0	9
13607959	0.1	6.2
13607964	0.7	35.6
13607965	40.4	6.2
13607969	0.7	3.9
13607971	77.2	149
13607973	2.7	1
13607976	5.8	2.7
13607980	8.0	4.6
13607982	11.6	1
13607990	37.0	20.6
13607992	26.7	1
13607994	40.1	97.6
13607995	18.6	28.8
13607997	24.1	15.7
13607998	41.7	23.9
13608001	47.2	4.8
13608003	0.3	2
13608006	49.1	19.5
13608010	45.8	16.4
13608018	35.0	16.6
13608020	57.8	44
13608021	23.4	6.3
13608023	25.3	1
13608025	22.3	11.1
13608071	5.8	49.8
13608072	34.8	1
13608073	39.6	9.5
13608074	0.7	2.8
13608077	23.1	25.4
13608085	20.3	7.8
13608087	66.3	8.4
13608092	30.8	25.3
13608094	9.0	10.9
13608099	0.1	90.7
13608102	55.9	24.5
13608103	33.9	16.2
13608104	55.9	15.6
13608106	54.2	8
13600001	0.3	1.4
13600003	1.9	2.2
13600004	10.5	2.4
13600005	48.0	48.3
13600006	35.0	4.1
13600009	34.7	58.1
13600010	2.9	1
13600012	3.9	2.7
13600018	10.9	1.1
13600023	8.5	1.9

13600025	0.9	1
13600026	11.8	1
13600027	11.6	2.7
13600028	21.1	17.1
13600029	10.5	10.1
13600030	18.8	112.5
13600032	9.7	20.2
13600036	14.2	203.2
13600037	13.1	1
13600038	9.6	5.8
13600040	12.2	9.2
13600042	13.3	1
13600043	18.0	1.7
13600044	18.0	114.4
13600045	4.2	60
13600046	37.7	14.4
13600047	38.5	1
13600048	21.3	7
13600051	51.7	38.3
13600052	25.1	1
13600053	26.4	1.6
13600054	59.8	12
13600059	24.0	12.1
13600060	41.2	2.5
13600061	52.5	39.8
13600063	24.6	29.6
13600064	41.7	13.4
13600065	57.4	2.2
13600067	37.7	9.5
13600070	71.9	1.1
13600072	56.3	12.4
13600077	68.3	35.9
13600078	71.4	13.2
13600080	76.4	11.9
13600081	0.6	9.6
13600082	71.5	1
13600083	66.6	31.7
13600087	69.0	14.6
13600089	72.2	18
13600090	69.5	4.2
13600091	15.6	36.8
13600092	69.6	55.2
13600093	15.1	20.1
13600095	65.2	2.5
13600098	17.1	27
13600100	2.0	1
13600102	4.0	1
13600111	63.3	1.5
13600112	64.7	9.6
13600114	48.3	19.7
13600116	27.7	1.5
13600118	28.4	15.5
13600121	34.2	24.9
13600125	45.1	50.4
13600131	7.9	5
13600132	72.1	2.7
13600133	20.1	2.4

13600142	4.0	13
13600143	67.7	7
13600145	65.8	1.2
13600149	4.3	45.9
13600150	59.2	3.3
13600152	45.9	63.5
13600153	42.2	2.5
13600155	52.7	16.7
13600156	59.1	18.9
13600158	1.9	1.7
13600160	57.4	7.5
13600162	36.6	17
13600163	39.8	1.3
13600164	78.2	47.1
13600167	59.0	44.4
13600170	12.2	8
13600172	11.7	1
13600175	57.4	38.7
13600177	0.7	17.6
13600180	16.5	9.8
13600181	21.0	1.7
13600182	61.4	49.6
13600183	13.8	1
13600184	9.1	1.5
13600186	14.5	2.9
13600189	8.0	5.5
13600190	39.5	10.7
13600191	8.0	26.9
13600194	64.3	21.2
13600196	49.7	1
13600197	21.3	44.2
13600204	43.5	28.6
13600205	49.3	14.1
13600210	65.1	4.6
13600212	26.1	20.7
13600214	36.9	19.8
13600215	15.2	34.1
13600217	59.3	57.6
13600219	48.3	4.4
13600220	51.4	9.7
13600221	51.0	1.9
13600222	39.2	44.1
13600227	0.4	82
13600228	13.4	6.1
13600231	12.1	2.8
13600233	11.7	4.4
13600235	10.3	8.4
13600236	6.5	1.9
13600238	11.2	8.2
13600241	9.4	2.1
13600243	33.9	39.2
13600245	1.0	48.9
13600250	11.9	2.7
13600251	4.6	31.6
13600252	54.9	1.6
13600254	47.0	17.8
13600256	47.5	22

13600257	12.5	2.3
13600259	0.4	115.6
13600260	79.6	39
13600261	1.6	47.8
13600270	3.6	1
13600273	1.6	21
13600277	71.0	4.4
13600279	4.4	11.9
13600280	2.5	2.6
13600281	67.6	20.5
13600285	66.7	3.6
13600286	4.5	10.1
13600290	64.2	26.2
13600291	17.7	1.1
13600292	63.5	1
13600293	65.0	11.4
13600295	18.0	27.4
13600297	30.8	14.2
13600298	15.4	31
13600299	39.1	1.2
13600300	59.6	19.5
13600301	52.0	2.6
13600302	1.3	17.7
13600303	37.4	11.6
13600304	5.2	1.2
13600305	58.9	96.2
13600307	4.2	215.9
13600308	20.9	100
13600309	40.7	1
13600310	59.6	28.6
13600311	19.5	131.4
13600312	5.0	4.6
13600313	51.9	11.5
13600314	14.1	12.5
13600317	1.4	22.1
13600318	48.3	111.6
13600319	8.0	10
13600320	6.0	13.4
13600321	0.9	9.2
13600322	36.2	2.7
13600325	9.7	17.2
13600326	10.6	1
13600329	47.7	1
13600330	10.5	1.3
13600332	33.5	2.2
13600334	37.9	20.1
13600338	4.5	4
13600343	27.8	19.2
13600345	48.1	1.5
13600350	79.7	2.2
13600351	10.1	1
13600352	10.4	1
13600353	1.0	483.8
13600354	12.4	4.3
13600356	39.6	5
13600358	12.7	11.2
13600365	14.2	35

13600367	56.5	1
13600370	52.3	1.5
13600371	42.2	66.6
13600376	37.0	4.3
13600446	53.1	7.9
13600448	29.7	47.3
13600449	25.2	18.4
13600452	62.5	24.2
13600453	4.9	1
13600455	65.5	1.6
13600457	67.1	31.4
13600459	71.8	36.2
13600460	0.7	12.3
13600461	74.0	4.6
13600463	78.8	12.8
13600464	51.4	17.3
13600465	78.5	140.1
13600470	14.9	2.5
13600471	33.2	13.1
13600474	58.2	16.9
13600475	56.2	2.2
13600479	46.6	7.3
13600480	19.5	22.7
13600482	34.1	10.3
13600484	62.7	6.1
13600485	70.2	18.2
13600487	38.8	8.1
13600491	3.9	1
13600492	40.6	11.3
13600494	32.2	851
13600499	20.6	42.2
13600501	44.1	12.4
13600502	80.0	5.3
13600511	66.4	14.7
13600512	64.4	3.5
13600513	65.3	12.1
13600514	65.5	40.1
13600516	62.6	2.6
13600519	16.9	1
13600520	53.7	7.7
13600522	73.3	13
13600523	74.8	44.5
13600524	73.6	2.5
13600525	72.4	1
13600526	70.0	13.1
13600529	65.1	13
13600531	0.4	137.6
13600532	15.8	32
13600534	65.0	20.1
13600536	63.5	7.6
13600538	60.7	7.5
13600539	60.6	47.4
13600540	34.6	52.8
13600541	47.8	1
13600544	55.2	3.4
13600545	38.9	286
13600547	54.2	6.7

13600548	51.3	3.2
13600553	14.0	13.4
13600554	43.1	4.5
13600555	18.9	61.5
13600601	4.3	1.9
13600602	70.7	6.6
13600603	49.5	29.4
13600605	29.7	9.6
13600609	19.5	8.4
13600610	41.1	9.5
13600611	43.1	133
13600615	73.4	27.1
13600617	61.5	23
13600621	54.8	4.8
13600622	43.6	5.4
13600626	64.4	43.9
13600628	34.7	3.3
13600635	14.5	1
13600636	41.4	2.7
13600675	66.4	3.8
13600676	66.8	12.1
13600680	71.1	1
13600681	71.6	27.4
13600682	72.7	3.7
13600684	0.8	55.5
13600685	73.2	24.8
13600687	37.2	5.6
13600688	77.3	14.5
13600689	21.0	36.7
13600691	73.1	11.1
13600695	2.1	5.3
13600696	3.8	1
13600702	62.8	6.7
13600703	61.9	11
13600704	61.3	230.5
13600705	42.2	4.3
13600707	43.4	25.7
13600708	48.9	13.9
13600710	28.6	3.6
13600711	58.7	25.4
13600720	66.1	14.1
13600722	36.8	8.8
13600726	58.3	15.2
13600730	59.1	106.2
13600731	2.7	1
13600742	0.9	3.7
13600743	22.9	7.9
13600744	11.4	19.4
13600745	3.5	4.7
13600748	0.4	95.3
13600749	75.1	48.4
13600752	11.4	1
13600753	7.7	79.1
13600755	31.4	16.9
13600757	16.3	6.6
13600758	42.0	30.3
13600759	7.9	3.6

13600761	0.7	44.1
13600763	11.4	27.1
13600767	7.9	1.3
13600770	12.7	1
13600772	52.5	10.2
13600773	46.9	8.4
13600774	53.9	53.6
13600776	40.4	1.5
13600777	6.6	12.2
13600778	42.9	1.5
13600781	58.4	1
13600783	50.4	10.4
13600784	20.5	2.1
13600785	56.5	3.9
13600786	27.3	4.3
13600793	76.7	12.5
13600794	66.5	30.4
13600796	36.5	4.1
13600799	38.3	42.5
13600803	0.2	5
13600807	70.4	6.8
13600808	71.0	14.8
13600809	1.2	11.3
13600810	78.4	2.8
13600813	0.8	20.2
13600814	73.8	43.3
13600819	67.3	1.7
13600821	65.5	13
13600822	65.7	7
13600827	61.7	3.8
13600830	61.4	26.6
13600832	60.1	19.6
13600833	23.8	4
13600835	58.7	5.9
13600837	27.5	2.1
13600839	70.5	21.9
13600840	42.5	36.3
13600841	50.9	34.4
13600843	53.4	1.4
13600848	51.7	2.2
13600849	25.0	21.3
13600850	43.8	49.7
13600853	10.9	1
13600854	36.7	20.7
13600857	2.1	20.9
13600858	57.1	1.1
13600859	32.6	1.1
13600860	11.4	26.1
13600862	55.4	28.6
13600863	11.5	360.1
13600868	0.2	1
13600870	10.5	44.6
13600871	44.0	106.5
13600872	57.1	33.2
13600873	66.6	47.5
13600875	60.7	36.7
13600879	5.2	13

13600880	10.2	1
13600883	3.3	1.4
13600884	6.7	47.8
13600887	59.4	68.9
13600889	5.7	12.6
13600890	62.8	4.5
13600891	5.9	42.7
13600892	11.4	23.9
13600896	12.5	29.1
13600902	16.3	1
13600904	52.8	35.2
13600906	7.0	2.7
13600909	1.0	107.7
13600910	58.8	3.8
13600913	20.4	52
13600915	30.3	4.7
13600916	4.0	17.2
13600917	53.7	56.2
13600918	22.9	23.5
13600920	20.7	5.4
13600921	35.8	4.4
13600922	57.2	1.4
13600925	50.8	5.7
13600926	55.6	6.8
13600928	3.3	1
13600929	47.0	8.4
13600930	41.4	117.5
13600933	4.7	29.3
13600935	73.5	8.6
13600936	74.8	64
13600938	51.7	28.2
13600945	3.9	1
13600946	76.6	31.5
13600947	78.2	11.9
13600952	74.5	45.9
13600956	72.6	3.4
13600957	12.2	8
13600963	74.0	47.2
13600964	3.4	1
13600965	14.8	11.4
13600968	71.9	10.7
13600969	42.8	3
13600971	66.3	3.1
13600972	65.3	18.6
13600973	64.7	32.5
13600974	43.0	36.2
13600975	60.7	30.8
13600976	34.3	1.1
13600977	5.5	6.3
13600979	19.1	9.7
13600985	67.2	1
13600986	65.5	13.7
13600987	16.4	14.5
13600994	61.2	12.7
13600995	61.7	1.3
13600998	60.9	18.1
13601002	16.9	9

13601003	58.8	4.8
13601004	2.9	1.7
13601007	32.8	3.9
13601008	25.5	8.5
13601010	39.5	1.2
13601011	49.1	8.4
13601012	66.8	5.2
13601013	57.2	41.1
13601015	23.4	11.7
13601016	11.4	133.6
13601017	10.5	4.4
13601019	49.8	3.8
13601021	35.9	39
13601022	55.0	1.5
13601024	25.5	2.4
13601025	31.1	2.9
13601026	55.2	1.6
13601029	24.1	13.6
13601030	49.4	19.8
13601032	54.9	8.3
13601035	44.2	4.9
13601036	26.8	1.1
13601040	28.9	162.5
13601043	24.2	6.8
13601046	23.6	5.9
13601047	44.9	4.9
13601049	76.3	17.1
13601050	33.5	24.5
13601051	23.5	47.3
13601052	53.4	13.1
13601055	59.0	40.1
13601058	4.2	112.3
13601059	21.5	8.6
13601061	16.1	13.1
13601062	18.1	21.7
13601063	31.8	4.8
13601085	41.1	6.1
13601086	0.4	85.3
13601088	55.3	46.1
13601091	12.1	42.6
13601092	8.7	1
13601094	1.0	61.1
13601097	68.2	16.9
13601098	50.8	15
13601213	1.0	182.1
13601214	25.4	258
13601230	47.0	55.1
13601231	64.5	4.5
13601233	44.8	18.6
13601238	21.8	111.9
13601239	37.1	1.6
13601241	63.2	3.2
13601244	3.3	1.4
13601245	74.8	17.5
13601247	72.6	108.6
13601249	66.6	5.6
13601250	64.7	42.9

13601251	23.6	35.7
13601255	52.6	8.1
13601257	69.6	24.9
13601261	39.9	13.6
13601262	72.3	11
13601264	67.1	22.8
13601266	71.8	22.8
13601268	66.2	86.9
13601270	22.4	28.8
13601271	20.1	1.5
13601272	40.9	1
13601273	59.4	8.7
13601274	25.5	1.9
13601275	35.3	3
13601280	11.9	21.7
13601282	42.1	3.8
13601283	30.0	88.5
13601297	67.6	34.4
13601302	4.7	89.6
13601304	9.2	1
13601306	9.8	13
13601307	12.6	1
13601308	6.3	37
13601311	11.2	2.4
13601312	9.6	1
13601315	63.3	135.3
13601317	57.0	8.9
13601318	17.1	1
13601323	31.6	58
13601324	43.9	23.9
13601326	27.9	11.3
13601327	6.6	2.1
13601329	78.1	52.3
13601331	77.3	5.6
13601332	46.9	9.6
13601334	48.6	15.6
13601335	43.5	8.9
13601336	37.8	7
13601337	0.2	17.1
13601338	19.7	6.3
13601339	73.1	3.1
13601340	0.5	83.3
13601341	73.7	5.7
13601342	72.8	3.8
13601343	72.3	19.2
13601344	72.8	4.7
13601345	0.6	59.9
13601346	72.2	62.4
13601348	77.8	78.1
13601351	9.0	2.1
13601354	60.3	7
13601355	73.0	28.3
13601356	60.8	9.6
13601357	67.4	95.7
13601359	18.2	9
13601360	69.2	5.3
13601364	65.0	20.9

13601369	58.5	21.6
13601370	48.4	4.8
13601374	61.6	2
13601375	63.4	22.5
13601377	60.5	9.7
13601382	22.4	3.3
13601388	40.5	22.7
13601389	51.3	3.9
13601391	47.9	3.4
13601393	58.1	26.7
13601395	35.4	1
13601396	41.6	2.9
13601401	0.8	15.5
13601402	30.4	18.4
13601404	52.3	16.4
13601405	8.4	29.8
13601406	5.3	1
13601407	34.2	21.5
13601412	20.2	60.4
13601414	45.5	6.2
13601415	10.7	36.1
13601416	10.2	21.2
13601417	3.1	1
13601418	20.5	262.9
13601419	58.5	13.7
13601423	47.9	1.7
13601425	53.1	156.5
13601426	11.1	29.3
13601428	11.7	199.2
13601432	13.3	338.3
13601433	8.9	1
13601439	10.0	76.7
13601441	18.4	7.3
13601445	38.8	10.9
13601448	28.0	16.3
13601449	1.9	1
13601450	40.0	2.3
13601451	55.7	18.6
13601452	23.6	1
13601453	55.3	7.4
13601454	56.5	1
13601455	45.0	257.8
13601456	20.6	2.9
13601459	36.3	16.3
13601460	59.5	1
13601462	27.4	17.6
13601463	51.3	8.7
13601468	27.8	1.6
13601469	79.2	61.6
13601470	59.6	3
13601471	42.6	29.7
13601473	76.5	3.5
13601474	77.0	98.4
13601478	71.3	23.7
13601479	69.2	3
13601480	65.1	1
13601481	0.6	30.7

13601483	3.3	6.7
13601487	71.2	51.7
13601490	4.8	1.8
13601491	0.7	13.4
13601492	67.9	15
13601496	64.0	8.7
13601497	61.1	4.7
13601499	23.1	3.2
13601500	33.0	2.5
13601501	60.6	7
13601503	50.6	132.3
13601504	28.7	85.6
13601505	42.3	6.7
13601506	28.5	2.8
13601507	53.0	4.9
13601509	23.9	1
13601510	29.7	2.2
13601514	20.2	20.6
13601515	26.6	35.5
13601516	21.7	20.4
13601517	44.2	6.8
13601519	58.5	10.6
13601525	39.0	8.8
13601526	63.6	24.1
13601528	67.9	5.2
13601530	56.2	4.7
13601532	35.7	25.2
13601534	58.1	68.9
13601535	3.2	1.8
13601539	59.0	25.3
13601540	37.3	21.1
13601542	60.9	26.9
13601543	23.7	25.6
13601547	63.2	9.1
13601548	63.9	44.1
13601551	0.9	17.2
13601553	11.0	9.9
13601562	51.2	14.6
13601564	28.8	5.5
13601565	22.2	23.8
13601566	52.3	46.4
13601569	4.4	19.1
13601570	63.2	8.6
13601571	1.8	2.3
13601573	11.1	8.8
13601574	31.0	12.8
13601576	27.2	14.1
13601579	57.8	71.2
13601580	0.6	53.5
13601581	57.5	7.7
13601582	60.1	3.1
13601589	76.7	3.4
13601590	77.0	15.3
13601591	76.5	83.4
13601593	2.9	1
13601594	0.9	9.9
13601595	0.5	107.6

13601596	0.5	27.5
13601597	11.5	98.4
13601598	2.4	1
13601599	11.7	10.1
13601604	78.6	47.1
13601605	76.8	11.5
13601607	60.1	209.9
13601609	5.5	30.1
13601610	2.9	1
13601613	36.0	4.8
13601616	9.9	167.4
13601618	4.1	30.8
13601619	38.7	53.4
13601620	14.8	24.7
13601621	29.2	38.5
13601622	74.0	7.4
13601623	67.2	21.2
13601625	54.1	3.1
13601627	62.1	1.6
13601628	47.7	5.8
13601631	11.8	27
13601635	12.3	1
13601638	45.8	23.3
13601640	19.3	1
13601642	71.4	1
13601643	70.2	1
13601644	72.9	7
13601645	70.2	23.9
13601648	0.5	5.8
13601649	69.3	6.2
13601652	42.3	4.3
13601656	8.1	3.8
13601660	22.6	22.8
13601661	65.5	1.8
13601663	25.2	35.6
13601665	42.5	17.2
13601666	40.3	5
13601667	60.8	314.9
13601668	47.0	6.7
13601669	38.1	6.1
13601672	58.7	3.4
13601675	16.2	4.7
13601678	66.9	20.2
13601681	22.3	3.6
13601682	49.2	61.8
13601684	45.9	19.2
13601685	0.5	54.1
13601686	27.2	1.4
13601687	34.0	21.8
13601688	47.3	252.5
13601689	48.9	23.4
13601691	13.4	2.4
13601692	26.3	4.4
13601693	21.2	9.3
13601696	46.5	61.2
13601697	33.4	7.2
13601698	35.7	4.7

13601699	15.0	5.8
13601700	50.5	8.5
13601701	44.8	2
13601702	33.0	8
13601703	20.0	6.1
13601704	4.1	64.8
13601706	55.3	51.6
13601707	0.7	43.3
13601709	0.4	87.2
13601710	31.5	10.8
13601711	6.4	9
13601712	25.5	20.6
13601713	57.2	143.6
13601714	7.7	27.5
13601716	32.0	28.8
13601717	74.2	8.3
13601718	39.2	1.9
13601719	69.6	12.7
13601722	66.8	16.9
13601724	60.5	56.3
13601726	9.4	1
13601727	43.7	8.9
13601728	22.9	1.6
13601730	78.2	1.6
13601734	75.7	58.1
13601735	69.7	2.3
13601737	73.6	1.6
13601738	0.4	32.8
13601739	7.0	33.5
13601740	67.1	22.5
13601741	70.1	4.8
13601743	73.6	232.4
13601744	55.2	25
13601746	64.7	19.8
13601750	60.9	4.6
13601751	60.5	7.8
13601754	15.7	1.5
13601755	18.8	29
13601757	34.2	13.6
13601758	20.1	2.9
13601760	27.8	1.2
13601765	27.9	13.6
13601766	53.6	1
13601767	51.8	10.8
13601769	26.8	1
13601770	37.1	3.5
13601771	34.1	2.7
13601772	20.7	41.2
13601773	26.8	7.4
13601774	3.4	1.3
13601775	57.5	4.4
13601776	39.1	4.2
13601778	32.8	9.1
13601780	49.7	13.2
13601782	25.1	1
13601784	55.5	4.4
13601788	45.9	1.4

13601791	37.8	152.1
13601792	71.1	2.8
13601793	39.0	14.4
13601794	1.4	103.2
13601795	49.1	12.4
13601798	0.4	162.2
13601802	51.6	2.7
13601813	8.3	38.7
13601814	9.6	22.2
13601815	42.1	94.7
13601818	21.8	77
13601820	18.8	13.7
13601822	19.0	103
13601823	21.8	8
13601824	49.9	1
13601825	13.0	2
13601831	28.6	3.9
13601832	22.7	1.4
13601833	11.7	49.2
13601834	42.3	116
13601836	55.3	1.2
13601837	6.4	8.1
13601840	67.9	23.5
13601841	11.8	16.7
13601843	20.7	28.4
13601844	77.3	10.6
13601846	29.8	66.3
13601847	17.1	1.6
13601848	73.1	16.3
13601849	16.2	6
13601854	2.6	1
13601857	69.4	1.9
13601860	63.2	4.2
13601861	63.4	7.1
13601862	65.4	27.3
13601864	68.0	14
13601865	60.2	3.9
13601867	61.5	96
13601868	23.9	10.2
13601869	51.6	7.1
13601870	53.6	6.9
13601871	40.3	1.6
13601872	33.8	2.8
13601873	17.1	48.8
13601874	17.5	1
13601876	42.7	107.3
13601877	41.1	222.2
13601878	23.2	26
13601884	27.0	4.7
13601886	32.0	11.8
13601890	60.1	13.8
13601891	52.0	24.9
13601892	26.6	5.7
13601893	15.6	1
13601894	48.5	6.8
13601895	26.2	9.3
13601897	20.6	5.4

13601898	20.7	22.5
13601903	30.6	8.4
13601904	16.5	4
13601906	29.6	134.2
13601910	25.8	1.6
13601911	21.8	31.4
13601913	28.5	5.4
13601914	36.7	47.6
13601915	0.2	3.9
13601916	57.3	11
13601919	24.5	5.2
13601920	1.6	3.5
13601921	0.3	100.6
13601923	2.4	3.8
13601927	1.8	3.2
13601928	6.2	11.5
13601932	8.3	4.1
13601933	2.5	1
13601937	11.1	23.4
13601940	3.2	2.1
13601943	11.4	1
13601944	49.3	9.4
13601945	6.3	6.7
13601948	55.8	12.4
13601949	11.1	1.4
13601950	12.9	1
13601955	20.5	1
13601957	53.9	3.6
13601958	58.5	4.7
13601959	33.2	35
13601961	48.7	18.2
13601965	42.8	20.5
13601967	20.4	28.7
13601968	25.8	24.8
13601969	38.3	24.5
13601970	23.4	3.2
13601973	42.1	11.2
13601975	48.7	1
13601976	56.5	19.7
13601977	2.4	439.3
13601981	54.9	12.1
13601983	20.8	4.7
13601984	45.4	11.9
13602041	43.4	7
13602045	40.1	18.1
13602046	23.1	11.2
13602048	36.5	6.6
13602049	51.9	43.2
13602050	54.9	40.3
13602051	13.2	2.8
13602053	67.5	2.5
13602054	56.0	1.7
13602059	21.8	22.6
13602061	51.5	96.4
13602062	30.1	15.9
13602436	40.2	206.8
13602437	73.1	32.8

13602440	64.2	23.1
13602443	63.9	18.4
13602446	74.7	4.8
13602448	61.3	20.9
13602449	76.4	15.5
13602451	66.5	38.3
13602452	70.0	9.3
13602454	66.0	1
13602456	67.8	32.1
13602457	68.2	9.2
13602458	75.9	235.1
13602464	46.8	58.5
13602466	76.5	10.1
13602471	72.0	2.5
13602472	53.1	22.6
13602473	49.9	14.4
13602474	48.8	61.5
13602475	27.9	6.5
13602476	19.0	17.7
13602477	44.1	35.1
13602479	46.9	13.9
13602480	25.3	1
13602481	33.0	23.2
13602488	52.8	1
13602490	54.8	1.2
13602492	0.6	44.9
13602493	44.1	20.4
13602494	21.0	3.6
13602495	23.9	1
13602496	36.3	49.4
13602497	57.0	4.6
13602499	28.5	7.9
13602500	26.8	2.5
13602503	42.9	1.1
13602504	70.4	3
13602506	9.8	1
13602507	71.2	3.2
13602510	10.0	5.5
13602514	52.9	5.9
13602515	6.6	1.8
13602516	3.4	1
13602518	6.5	10.7
13602519	7.7	1
13602520	70.4	3.2
13602522	21.8	1.3
13602523	9.4	42.8
13602524	3.1	1
13602526	69.0	4.5
13602527	58.2	4.7
13602530	14.1	42.8
13602531	14.6	1
13602533	13.6	36.8
13602534	54.8	3.1
13602535	54.2	93.3
13602537	25.8	10.6
13602539	49.3	4.5
13602540	24.5	40.6

13602541	14.5	1
13602542	52.1	36
13602544	59.4	4.3
13602545	20.9	6.1
13602547	49.1	7.7
13602548	21.5	145.8
13602549	18.4	1.8
13602550	16.1	10.2
13602555	31.1	18.2
13602558	26.2	1
13602559	43.4	12.1
13602561	29.6	1.5
13602562	12.9	190.6
13602564	0.7	28.4
13602565	20.0	11.7
13602566	47.3	74.8
13602568	65.2	5.9
13602570	14.9	26.8
13602572	5.9	11.3
13602573	37.7	8.7
13602575	31.4	11.4
13602576	63.2	2.4
13602577	48.6	75.4
13602581	62.9	6.7
13602582	66.6	14.8
13602583	68.4	7
13602584	0.9	184.3
13602585	2.0	6
13602586	45.8	5.7
13602588	64.5	4.5
13602590	70.3	36.9
13602591	12.0	7.5
13602593	58.7	11.4
13602594	47.6	21.2
13602595	24.3	10
13602596	57.4	6.4
13602599	56.5	2.2
13602600	32.7	192.4
13602602	52.6	14.1
13602603	55.8	2.1
13602605	51.0	15.6
13602608	45.5	31.3
13602610	69.2	10.5
13602611	3.1	1
13602614	77.2	1
13602615	4.8	7.8
13602617	6.1	2.7
13602620	61.3	6.5
13602621	0.8	15.4
13602624	61.7	12.2
13602625	73.4	11.9
13602626	5.5	1
13602628	0.2	4.4
13602631	7.2	6.7
13602633	43.4	18.2
13602634	16.0	6
13602636	75.8	10.4

13602637	9.9	1
13602638	15.9	49.3
13602639	15.0	1
13602641	14.1	1
13602642	62.7	7.1
13602643	36.9	3.2
13602647	44.9	42.4
13602648	27.8	27
13602649	31.0	15.3
13602650	53.9	15.8
13602651	26.9	18.8
13602652	4.8	3
13602654	12.8	7.2
13602655	30.7	2.6
13602656	30.4	206.5
13602657	46.2	4.6
13602658	8.2	9.4
13602659	23.5	25.9
13602665	59.2	17
13602666	21.9	10.6
13602668	0.6	14.1
13602670	22.7	20.8
13602674	65.0	58.7
13602677	64.2	1
13602678	65.9	7.8
13602680	3.1	1
13602682	4.1	13.4
13602683	2.2	2097.9
13602684	40.2	1
13602685	69.7	15.4
13602686	0.9	26.6
13602688	61.3	9.3
13602689	67.2	14.4
13602690	76.7	27.6
13602692	26.7	15.9
13602694	67.7	1.5
13602698	56.7	25.4
13602699	17.2	33.7
13602700	56.8	9.1
13602701	50.6	9
13602702	27.1	8.3
13602703	53.9	37.5
13602704	42.6	13.1
13602706	26.3	10
13602707	29.9	5.1
13602709	28.3	11
13602710	41.5	20.1
13602711	51.1	6.2
13602713	30.5	3.2
13602714	29.0	19.1
13602715	21.1	102.8
13602716	22.9	9.6
13602717	56.3	33.3
13602718	48.2	15.7
13602720	3.6	1
13602721	65.9	40.5
13602722	23.4	15.1

13602724	19.4	2.2
13602725	35.8	8.2
13602727	64.7	5.2
13602728	3.3	1
13602729	17.4	4.1
13602732	57.4	50.4
13602734	66.7	58.7
13602736	20.7	1.7
13602742	1.3	15.8
13602744	11.5	3.1
13602746	65.6	1
13602750	9.6	2.7
13602753	5.3	5.6
13602755	76.1	10
13602756	61.2	28
13602758	16.0	51.9
13602760	68.0	28.9
13602761	9.3	4.7
13602763	29.5	68.6
13602764	48.9	177.1
13602765	1.2	37.4
13602766	59.8	14.5
13602767	51.2	1.7
13602768	19.8	4.2
13602769	37.2	94.9
13602770	54.6	8.6
13602772	49.2	4.5
13602778	21.8	18.6
13602779	51.5	4.1
13602781	52.6	14.1
13602784	23.7	6.3
13602788	55.8	1.8
13602789	54.3	70.9
13602791	2.1	13.8
13602792	22.0	19.6
13602793	42.1	22.6
13602795	12.9	31
13602798	30.2	2
13602800	78.9	6.1
13602801	67.3	49.9
13602803	63.2	4.2
13602804	41.7	108.9
13602806	63.0	10.3
13602808	2.5	1
13602809	65.0	8.2
13602810	73.6	3.2
13602812	71.4	14.4
13602815	3.5	1.1
13602817	62.2	8.7
13602820	76.1	68.6
13602821	14.5	1
13602822	11.9	7.7
13602823	66.7	2.4
13602825	46.3	6.4
13602826	13.3	17.8
13602827	23.7	37.5
13602829	25.9	10.1

13602832	22.2	1
13602833	55.5	1.4
13602838	53.9	18.9
13602844	46.5	3.2
13602845	22.8	191
13602846	55.7	8.6
13602847	38.8	21.1
13602848	23.0	65
13602849	42.2	3.7
13602851	46.0	40.8
13602853	5.0	2.5
13602856	57.5	50.6
13602858	5.8	4
13602859	2.0	13.9
13602861	1.0	108.8
13602862	78.5	13.2
13602865	66.5	6.4
13602871	4.7	143.5
13602872	11.5	1
13602875	11.2	8.3
13602876	10.8	1
13602877	5.8	5.2
13602878	6.0	9.8
13602883	10.9	17.7
13602884	76.1	21.5
13602885	12.6	1
13602886	9.9	1
13602887	16.5	1
13602889	16.7	6.7
13602890	32.4	10.5
13602891	26.8	1.8
13602892	58.1	53.6
13602896	25.8	8.8
13602897	45.3	11.4
13602899	25.0	1
13602900	0.6	28.9
13602901	47.6	20.2
13602904	49.4	6.6
13602905	26.3	179.2
13602906	59.5	40.3
13602908	17.7	15.6
13602909	57.6	1
13602911	56.6	11.7
13602912	27.2	1
13602913	58.7	114.1
13602915	43.9	4.2
13602916	13.0	26.9
13602919	32.1	5.1
13602920	17.7	1.9
13602921	31.5	1
13602923	43.2	5.3
13602925	58.5	5.2
13602926	33.7	1.1
13602929	56.8	5.9
13602931	30.7	24.8
13602932	29.0	15.2
13602933	28.9	29.9

13602934	43.7	1
13602935	51.6	4
13602938	11.8	236
13602941	6.9	40.4
13602942	27.7	3.4
13602945	7.4	1
13602946	60.4	29.7
13603021	46.0	1.6
13603022	56.7	36.5
13603023	42.6	68.1
13603024	66.6	1
13603026	33.5	4.6
13603027	26.1	4.1
13603031	45.6	12
13603032	9.8	1
13603033	0.9	124.6
13603034	62.4	80.1
13603035	38.3	5
13603036	52.2	14.6
13603129	60.9	1.1
13603130	0.2	1
13603131	60.9	10.5
13603132	66.7	2.6
13603135	76.7	1
13603139	61.2	61.6
13603142	0.3	32.8
13603143	0.2	1
13603146	67.9	515.4
13603148	61.4	103
13603150	0.9	8.6
13603152	0.3	12.1
13603153	74.6	2.3
13603154	19.1	14.3
13603158	60.6	2.2
13603159	4.2	84.2
13603162	61.9	23
13603163	13.1	29.5
13603164	15.1	2
13603167	15.1	1.3
13603168	14.3	20.7
13603169	12.4	5.9
13603170	72.0	6.5
13603171	12.8	28.6
13603172	67.3	17
13603174	6.7	2.2
13603176	65.4	5.9
13603178	40.7	27.1
13603179	55.1	16
13603180	28.9	35.5
13603186	63.7	14.4
13603187	47.6	1.3
13603188	20.1	19.7
13603189	59.9	9
13603192	34.9	60.2
13603193	52.0	6.2
13603197	12.2	6.7
13603201	13.3	32.4

13603202	14.3	14.9
13603204	62.2	18.6
13603205	24.9	9.8
13603206	23.3	8.3
13603209	71.6	105.4
13603214	9.3	46.4
13603215	3.3	4.6
13603216	48.8	33.9
13603220	10.4	21.9
13603221	69.1	22.8
13603222	77.3	65.2
13603223	9.2	13.5
13603227	6.6	6.9
13603233	7.2	1.7
13603234	11.2	26.9
13603236	69.4	5.7
13603242	55.5	4.2
13603243	29.4	5.8
13603244	38.7	7.1
13603245	0.9	1
13603248	58.4	55.8
13603250	29.6	9.2
13603251	25.8	12.4
13603252	6.2	2
13603254	28.4	283.3
13603256	41.4	150.8
13603257	40.9	8
13603258	56.3	51.8
13603260	55.7	1
13603263	34.3	20.6
13603264	43.2	2.8
13603265	14.1	21.2
13603267	32.5	11.1
13603269	20.5	2.9
13603270	49.0	139
13603271	20.9	33.2
13603272	35.2	4.2
13603274	51.9	12.3
13603276	31.3	1
13603278	55.4	17.2
13603281	26.5	3.1
13603282	53.4	4.1
13603284	3.2	1.4
13603309	68.4	15.5
13603311	62.1	6.4
13603312	3.3	1
13603313	52.3	4.7
13603314	40.3	18.3
13603315	61.3	16.3
13603316	52.6	13.8
13603317	28.5	19.3
13603322	73.3	13.1
13603324	78.9	1
13603325	64.0	32
13603326	61.0	17.4
13603327	68.3	2.6
13603331	70.6	26.8

13603332	70.0	32.5
13603333	67.0	1
13603339	14.3	1.2
13603340	14.8	1.3
13603341	0.6	79.9
13603342	66.3	8.7
13603345	65.1	82.6
13603346	73.8	197.1
13603347	15.0	12.8
13603348	65.0	21.6
13603349	73.2	2
13603351	72.3	18.6
13603354	60.5	123.1
13603357	57.4	6.4
13603358	32.6	1
13603359	18.9	1
13603360	11.7	3.2
13603363	39.3	18.3
13603364	43.4	14.6
13603366	52.6	18.9
13603372	39.7	2.9
13603373	51.3	15.2
13603375	54.7	1
13603376	53.6	3.7
13603377	46.4	18.7
13603380	33.8	56.1
13603381	45.4	17.5
13603382	30.1	4.3
13603383	24.6	1
13603384	27.5	1.5
13603386	44.5	3.7
13603387	55.2	59.8
13603388	28.3	39
13603390	26.6	59.8
13603391	23.6	11.2
13603392	19.0	30.2
13603393	46.1	6.6
13603394	17.7	33.9
13603395	25.3	9.6
13603396	36.3	3.6
13603397	28.6	2.5
13603400	34.8	93.3
13603403	67.0	21.3
13603404	64.3	17.2
13603405	9.5	1
13603406	7.5	2.5
13603407	35.2	23.8
13603408	39.3	1
13603409	69.0	37.4
13603411	8.9	1
13603413	68.4	12.2
13603415	2.5	4.2
13603416	6.1	2.6
13603418	0.6	13.3
13603423	34.4	50.2
13603425	14.9	1.6
13603427	17.0	21.7

13603428	8.7	15.3
13603429	3.3	2.2
13603432	67.4	21.6
13603433	77.2	4.4
13603434	63.4	1.9
13603435	63.9	21.4
13603436	6.5	2.3
13603437	45.5	53.6
13603438	10.8	1
13603439	64.7	37.5
13603440	24.6	22
13603441	41.0	441.5
13603443	43.5	65.2
13603444	13.7	1
13603445	23.0	19.6
13603447	53.1	2.3
13603449	21.2	162.6
13603451	56.5	7.3
13603453	24.6	36.7
13603457	49.7	3.3
13603460	27.1	2
13603462	17.7	17.6
13603463	42.4	5.1
13603465	30.8	2
13603466	23.4	25.9
13603467	41.1	15.4
13603469	14.1	8.2
13603471	15.2	4.9
13603475	67.8	7.2
13603477	56.0	5.2
13603478	1.3	27.2
13603479	6.6	1
13603481	7.4	8.1
13603482	0.6	28.2
13603484	10.6	3.6
13603485	6.8	11.6
13603487	63.4	7.3
13603488	12.0	2.6
13603492	72.2	17.8
13603494	25.3	2.8
13603497	65.7	12.5
13603498	60.1	43.4
13603499	35.7	4.3
13603500	7.0	1.5
13603501	78.6	3.8
13603502	57.7	23.6
13603503	25.7	3.2
13603504	1.4	61.3
13603505	18.4	10.2
13603506	29.0	65.6
13603508	8.9	2.6
13603511	27.8	6
13603513	26.7	18.3
13603517	51.5	21.8
13603518	27.9	30.4
13603519	18.2	4.6
13603525	59.3	1

13603526	12.0	9.7
13603529	43.4	2.2
13603530	26.8	3.2
13603532	21.6	10.8
13603534	17.8	2.3
13603536	20.1	2.3
13603540	54.5	4.5
13603542	42.3	46.4
13603544	53.6	4.1
13603545	37.9	8
13603547	34.0	49.4
13603548	32.7	1.4
13603549	29.4	4.6
13603551	50.7	25.3
13603552	23.7	4.6
13603553	27.8	7.4
13603554	35.7	5.4
13603555	24.9	8.2
13603556	30.2	15.3
13603557	39.5	1
13603558	1.2	17
13603560	43.1	6.4
13603564	60.0	7.7
13603566	60.6	107.3
13603569	74.9	13.1
13603570	14.5	1
13603571	14.7	3.6
13603573	13.9	81.3
13603575	15.9	32.8
13603576	29.4	6.2
13603584	0.9	26
13603585	64.2	15.1
13603586	64.2	24.5
13603587	28.5	4
13603588	0.3	35.2
13603589	67.9	2.9
13603590	59.5	62.5
13603595	40.2	3.4
13603596	40.8	17.8
13603597	16.8	1
13603598	25.1	1
13603599	55.9	6.5
13603602	4.3	2.2
13603607	57.1	3.1
13603613	38.9	4.3
13603615	31.7	13.2
13603616	1.0	202.6
13603618	1.4	142.3
13603623	77.2	2.3
13603624	35.1	1.2
13603627	77.0	75.3
13603628	62.7	7
13603630	59.1	21.5
13603631	42.2	26.7
13603633	76.8	83.9
13603635	40.0	1.7
13603636	40.1	11.7

13603638	62.9	7.1
13603639	39.3	1
13603641	75.2	14.7
13603642	64.5	1.5
13603644	61.0	111.6
13603645	71.3	12.3
13603647	75.7	28.3
13603648	78.1	67.6
13603649	26.2	1
13603651	69.4	13.9
13603654	76.3	411.4
13603662	62.8	55.6
13603663	72.2	1.6
13603664	37.3	7.8
13603668	64.8	15.5
13603669	20.1	1.9
13603671	48.0	2.6
13603672	12.0	1.1
13603674	26.4	1.1
13603675	23.5	6.7
13603679	1.2	180.9
13603681	28.2	3.2
13603683	47.6	3.2
13603684	26.4	7.6
13603687	26.8	5.2
13603688	26.3	4.6
13603690	4.1	8.8
13603691	49.8	8.6
13603692	19.7	7.6
13603693	26.8	9.9
13603694	0.2	6.3
13603695	69.3	20.3
13603698	20.9	1.1
13603699	25.3	1.4
13603700	23.5	49.4
13603701	58.9	17.8
13603702	0.8	18
13603703	50.1	30.4
13603705	18.3	1
13603708	28.0	7.4
13603709	17.1	34.5
13603710	62.0	18.2
13603711	22.6	4.8
13603718	69.3	1.1
13603720	28.3	13
13603721	23.9	28.5
13603724	55.5	8.6
13603725	9.0	119.7
13603726	26.6	4.2
13603727	4.6	75.1
13603728	76.0	51.2
13603730	0.9	40.4
13603731	6.8	3.2
13603732	68.7	1.6
13603735	33.1	13.6
13603736	71.3	1
13603737	78.4	2

13603739	8.0	10.6
13603740	5.0	1
13603745	14.2	4.7
13603747	67.7	34.6
13603748	44.1	156.8
13603749	42.7	5.3
13603751	69.3	2
13603752	34.3	57.5
13603753	48.2	6
13603754	11.3	11.2
13603755	8.8	1
13603757	46.1	37.3
13603760	3.6	2
13603761	4.8	5.1
13603764	26.3	20.3
13603766	32.1	122.4
13603768	52.3	10.3
13603769	18.1	945.7
13603771	34.1	28.2
13603772	50.9	8.7
13603774	20.2	31.2
13603776	22.8	7.4
13603779	32.4	3.5
13603781	28.8	1
13603783	27.4	12.1
13603785	59.5	4.6
13603786	18.0	7.4
13603787	4.5	32
13603791	33.2	74.5
13603793	22.5	14
13603796	20.5	9.4
13603800	59.4	3.7
13603801	65.3	2.9
13603803	76.7	5.5
13603804	40.3	16.8
13603805	64.5	7.1
13603806	76.8	9.4
13603810	61.3	33.3
13603812	69.7	50.3
13603815	23.9	8.4
13603818	32.1	21
13603819	0.7	20.9
13603820	66.2	20.1
13603821	46.6	14.4
13603825	78.3	3.5
13603826	47.5	38.6
13603827	66.2	25.1
13603829	75.7	13.8
13603831	16.6	165.6
13603833	71.0	42.1
13603834	68.8	1.3
13603836	61.7	1
13603838	28.2	8.4
13603839	66.6	1
13603840	52.7	36.6
13603842	28.2	12.7
13603844	43.6	21.9

13603845	46.7	32.5
13603846	21.1	20.1
13603847	38.5	1
13603848	21.8	4.5
13603849	23.8	17
13603852	25.1	23.2
13603853	59.6	2.3
13603855	54.2	1.5
13603856	50.7	12
13603857	49.4	23.7
13603860	68.2	2.6
13603861	67.8	8.9
13603863	67.8	23.5
13603864	37.6	1
13603865	22.8	83.3
13603866	49.3	25.1
13603867	41.8	10.9
13603870	1.3	15.9
13603878	0.8	2.9
13603886	17.9	16.1
13603890	42.1	131.4
13603895	8.1	5.4
13603897	10.2	19.6
13603899	73.5	9.6
13603900	10.7	3.1
13603901	6.5	2.4
13603902	5.5	24.7
13603906	49.5	6.3
13603907	1.6	8.8
13603908	44.4	8
13603909	27.5	53.4
13603910	45.2	2.5
13603911	63.5	4.9
13603913	48.6	7
13603915	46.0	95.3
13603917	18.7	6
13603919	70.5	86.5
13603922	76.5	1
13603924	33.6	24.8
13603925	47.2	37.2
13603926	35.6	1
13603928	16.3	14.7
13603930	72.6	6.8
13603931	35.2	9.3
13603932	27.3	18
13603933	40.5	13.2
13603934	39.8	12.5
13603940	58.6	24.3
13603942	20.5	32
13603943	72.6	4.1
13603944	21.0	1
13603947	37.8	14.9
13603956	14.2	11.6
13603970	4.8	12.9
13603972	59.9	16.6
13603994	2.1	1.7
13604033	6.6	12

13604057	12.9	2.7
13604072	62.7	50
13604078	28.3	3.1
13604096	71.6	28.9
13604103	75.3	7.8
13604110	14.3	2.5
13604111	66.1	4
13604122	62.7	17.1
13604129	54.3	9.4
13604135	73.4	46.2
13604138	37.3	12.1
13604162	8.7	9.8
13604172	74.3	6.6
13604173	16.9	1.9
13604176	51.5	24.9
13604179	29.8	1.2
13604182	27.4	10.6
13604191	5.5	9.4
13604208	54.4	10.5
13604212	28.2	16.9
13604218	17.8	2.9
13604219	28.2	6.2
13604222	56.8	33.7
13604223	12.6	4.1
13604224	64.3	7.7
13604237	0.7	25.4
13604242	10.9	161.5
13604243	37.4	39.9
13604293	69.9	12.8
13604295	42.5	45.9
13604296	1.0	35.8
13604301	0.8	158.9
13604302	19.1	9.2
13604303	31.6	13.7
13604304	46.8	1.8
13604305	18.5	9.7
13604306	41.9	65.5
13604308	56.9	1
13604311	60.6	1
13604315	55.0	4.7
13604323	2.5	10.5
13604337	18.7	19.7
13604352	72.3	2.6
13604357	25.3	2.2
13604360	63.0	5
13604367	49.7	107.7
13604369	50.3	54.9
13604370	53.2	13.1
13604371	26.3	2.3
13604372	36.2	12.2
13604374	68.5	28.4
13604375	70.1	23.8
13604376	67.8	23.4
13604381	64.7	14.2
13604382	60.1	2.5
13604383	67.9	7
13604386	16.0	1.2

13604388	16.1	1
13604389	13.0	43.9
13604390	41.7	4.1
13604391	7.2	2.1
13604392	7.1	1.7
13604393	32.7	14.7
13604395	54.5	53.6
13604396	20.4	3.9
13604398	42.4	19.2
13604399	19.3	16.1
13604400	71.3	8.1
13604401	5.7	556.5
13604403	23.1	25.4
13604406	35.3	11.9
13604408	35.0	9.3
13604411	26.7	4.9
13604412	12.6	10
13604414	39.7	5
13604415	21.3	6.1
13604417	34.7	15.8
13604418	28.2	1.1
13604420	8.9	1.3
13604421	24.5	1
13604422	43.5	29
13604423	57.7	12.6
13604426	20.7	1.9
13604427	4.0	1
13604428	14.1	15.2
13604434	39.4	3.6
13604436	51.6	14.9
13604437	18.7	1
13604439	33.8	4
13604440	5.2	5.9
13604441	43.3	75.1
13604442	60.7	55.8
13604445	12.0	1.7
13604446	23.4	1.7
13604447	68.3	1.6
13604450	39.6	21.5
13604451	5.0	6.1
13604453	1.0	106.5
13604454	74.1	2.7
13604455	67.5	10.1
13604456	62.4	63.5
13604461	1.1	119.1
13604470	60.0	7.2
13604471	12.0	29
13604473	10.2	8
13604475	27.4	1
13604477	59.5	9.4
13604478	37.7	3.6
13604479	9.5	59.6
13604480	21.0	100
13604482	30.1	16.3
13604483	56.5	14.7
13604484	31.9	1
13604486	33.0	11.2

13604488	59.1	6.3
13604489	31.3	38.6
13604490	21.5	1
13604495	18.0	11.1
13604496	18.9	1
13604498	13.2	2.9
13604500	36.5	18.2
13604501	32.6	14.8
13604502	53.0	18.2
13604504	43.7	3.7
13604505	77.3	3.6
13604506	66.2	2.8
13604509	61.0	30
13604510	50.6	9.6
13604511	36.4	8.5
13604512	38.5	2.2
13604515	68.9	35.5
13604517	66.6	30.9
13604518	21.8	1
13604520	0.7	12.3
13604521	48.3	11.6
13604522	21.6	2.8
13604523	75.7	11
13604525	78.0	4.8
13604528	39.6	684.2
13604530	27.5	1.9
13604532	77.3	28.4
13604533	61.2	7.6
13604535	55.5	184.8
13604536	66.8	25
13604539	61.0	9
13604541	40.8	12.1
13604542	59.7	16.5
13604544	49.2	1.9
13604545	39.9	6.9
13604547	13.9	3.7
13604551	46.2	13.2
13604552	9.1	1
13604553	29.9	29.4
13604557	29.2	1
13604559	26.4	87.7
13604560	0.6	26.9
13604561	21.0	51.7
13604562	20.3	81.5
13604566	44.5	22
13604569	25.8	49.7
13604573	76.4	6.5
13604574	39.8	11.7
13604575	39.8	4.7
13604577	36.2	9.8
13604579	35.3	26.2
13604584	15.3	10.2
13604586	38.1	24.9
13604587	60.6	11.9
13604591	31.4	1.7
13604592	0.3	3.1
13604593	1.2	88

13604594	4.8	106.9
13604595	54.9	1.7
13604597	50.2	109
13604598	2.5	1
13604602	73.4	26.8
13604611	11.6	1
13604613	4.7	1.1
13604616	78.2	7.1
13604617	75.9	7.6
13604619	12.1	2.4
13604620	62.3	63.8
13604623	36.1	42.1
13604624	34.9	13.5
13604627	55.4	3
13604628	21.1	1.1
13604629	33.9	5.6
13604632	49.6	11.7
13604647	48.6	31.5
13604649	28.2	4.2
13604651	58.8	11.6
13604652	17.6	5.3
13604655	40.4	1.9
13604656	61.7	2.3
13604658	68.9	10.6
13604660	72.5	11.7
13604661	0.3	7.8
13604663	61.1	5.4
13604665	64.3	6.1
13604667	67.8	67.8
13604669	1.9	9.6
13604670	29.8	1.2
13604674	45.2	6.5
13604678	67.4	2.2
13604679	14.3	6.1
13604680	12.3	108.4
13604681	13.0	3.7
13604682	78.1	28.2
13604683	66.9	42.9
13604684	12.2	47.9
13604686	6.7	1.2
13604687	62.5	3.8
13604688	36.0	4.7
13604689	78.8	15.1
13604690	44.7	8.8
13604691	25.8	3.4
13604693	70.3	11.4
13604694	62.7	12.6
13604696	15.4	9.6
13604697	54.1	42.2
13604703	26.1	5
13604707	38.1	41.1
13604709	28.8	4.1
13604712	0.7	4.3
13604713	58.9	117
13604715	15.5	24.6
13604716	23.4	35.3
13604717	19.8	1

13604722	16.1	7.4
13604723	32.4	2.7
13604724	76.9	6.2
13604725	31.9	1
13604726	3.8	1
13604728	18.6	1.4
13604731	55.3	2.9
13604733	69.1	32.3
13604740	33.3	13.8
13604741	35.5	26.1
13604743	29.1	1
13604744	79.5	3.9
13604746	11.5	1
13604747	46.1	3.8
13604749	79.2	12.3
13604750	62.6	12.4
13604751	47.5	60.3
13604754	78.5	9.4
13604755	1.5	7.6
13604762	75.4	22
13604764	10.9	14
13604767	57.8	1.1
13604769	24.2	23.6
13604770	30.9	4.3
13604778	22.9	1.8
13604781	28.3	1.8
13604783	9.0	3
13604784	38.8	9.2
13604786	51.9	2.4
13604787	52.5	4.2
13604788	33.0	28.1
13604794	28.0	4
13604798	9.7	60.2
13604799	24.6	18.7
13604800	31.8	115.6
13604803	52.2	10.2
13604804	66.0	75.5
13604806	70.0	134.3
13604807	58.1	1
13604809	60.3	22.4
13604813	77.9	71
13604817	4.1	4.6
13604820	3.9	2.1
13604821	76.1	18.6
13604825	10.7	7.9
13604828	14.1	28.4
13604830	71.2	20.3
13604831	75.7	42.6
13604832	9.2	206.9
13604833	67.3	23.4
13604834	28.1	2.9
13604835	73.9	1
13604838	56.4	3.3
13604840	35.7	6.4
13604841	16.8	3.5
13604842	8.9	15.9
13604844	28.2	4.3

13604846	31.2	165.8
13604849	53.8	4.3
13604850	25.4	280.1
13604851	34.7	7.1
13604852	29.5	22.3
13604853	42.5	6.6
13604862	47.8	1.5
13604865	0.2	1
13604866	71.9	83
13604871	54.9	74.9
13604873	10.4	21.9
13604878	78.6	2.2
13604880	3.5	1.2
13604884	3.2	3.9
13604886	9.3	1
13604888	26.8	27.2
13604891	21.8	17.7
13604892	34.5	12.8
13604896	20.5	9.6
13604900	66.1	2.7
13604902	69.1	18.7
13604903	43.8	11.4
13604904	53.0	25.1
13604905	78.0	60.3
13604906	38.5	3.7
13604907	29.8	19
13604908	62.4	2.3
13604909	70.6	1.3
13604910	65.4	1
13604912	78.8	26.9
13604913	60.6	1.4
13604916	68.7	32
13604917	72.3	86
13604919	4.9	12
13604921	12.5	6.4
13604922	52.8	9.3
13604923	15.6	1
13604925	67.4	30.7
13604927	70.8	5.9
13604929	41.7	23.3
13604930	66.0	3.4
13604931	12.5	7.4
13604933	50.7	3.5
13604935	47.5	2.3
13604936	13.5	17.6
13604938	48.1	10.8
13604939	30.5	16.3
13604941	37.1	45.2
13604942	18.8	1
13604943	46.2	6.6
13604944	19.6	69.4
13604945	34.8	7.9
13604949	51.8	1
13604950	58.7	28.3
13604951	56.3	28.3
13604953	15.3	23.4
13604955	34.5	114.4

13604956	65.3	22.4
13604959	34.8	20.5
13604963	46.8	13
13604967	17.0	6.3
13604975	31.3	5.3
13604976	55.7	5.4
13604978	26.0	1
13604979	35.5	17.8
13604980	29.8	41.8
13604981	0.2	7.9
13604983	40.6	148.6
13604989	10.6	34.2
13604991	0.8	13.2
13604993	9.9	19.5
13604996	23.7	2.2
13605002	54.1	1.4
13605005	60.3	3.2
13605006	26.1	5.3
13605007	68.3	8.7
13605008	20.5	11.4
13605013	37.4	4.9
13605016	42.9	3.2
13605023	23.2	3.2
13605026	58.5	9.9
13605028	35.8	171
13605033	14.7	4.1
13605034	31.9	6.1
13605042	47.5	8.5
13605044	28.5	27.9
13605049	53.5	22.3
13605051	2.7	2.3
13605052	20.9	24.8
13605054	8.0	3.4
13605055	1.4	21.8
13605056	76.8	4.4
13605061	20.9	55.4
13605066	14.4	11.4
13605067	65.7	7.1
13605068	21.1	25.8
13605069	14.7	199.5
13605074	54.4	1.3
13605075	48.2	6.5
13605078	67.5	1.9
13605079	19.6	8.6
13605081	25.3	1.4
13605083	42.2	1
13605085	40.0	5.6
13605087	18.8	25.4
13605088	50.6	3.8
13605090	0.8	11.9
13605095	12.5	1
13605097	50.6	1
13605099	0.6	20.2
13605101	50.1	1.5
13605103	39.6	2.1
13605105	70.8	10
13605107	21.7	9.4

13605109	2.9	1.2
13605111	1.4	7.4
13605112	70.8	1
13605113	33.0	30.2
13605115	10.4	4.2
13605119	67.1	15.5
13605121	27.6	4.8
13605123	7.2	2.3
13605125	41.9	839
13605126	10.7	43.4
13605127	58.2	49
13605129	79.0	5.3
13605130	68.5	5.2
13605133	10.8	86.3
13605134	38.1	1.9
13605139	0.3	10
13605141	10.9	7.4
13605144	32.5	43.1
13605145	10.6	1
13605146	31.2	2.6
13605150	32.9	5.1
13605152	9.1	4.9
13605156	43.0	26.6
13605158	24.4	13.8
13605160	23.1	20
13605162	22.9	1.6
13605164	34.3	17.8
13605165	59.9	24.7
13605166	28.8	8.4
13605169	47.8	41.3
13605170	46.8	25.8
13605172	49.0	14.3
13605173	0.3	60
13605174	49.7	28.9
13605178	60.5	67.9
13605179	23.3	19
13605180	49.0	8.2
13605182	20.5	9.6
13605185	31.4	83.5
13605186	57.0	4.9
13605188	0.2	3
13605189	26.9	11.7
13605190	18.9	94.9
13605193	32.8	33.7
13605196	38.3	12.1
13605198	32.6	27.5
13605202	64.3	81.4
13605203	69.4	18.3
13605205	63.1	32.1
13605206	35.5	2.7
13605207	63.6	3.4
13605209	65.2	128.6
13605211	73.2	25.8
13605212	77.9	4.5
13605214	2.8	1
13605218	55.0	2.9
13605219	79.9	15

13605220	60.6	7.7
13605221	77.0	141.4
13605222	62.7	22
13605224	65.4	1.6
13605225	69.8	29.7
13605227	11.9	209.9
13605231	7.6	51.8
13605232	9.2	38.8
13605235	8.5	95.3
13605236	6.5	13.7
13605237	4.9	4.3
13605238	12.7	54
13605239	4.8	15.2
13605240	16.6	1
13605243	29.2	6
13605248	39.0	20
13605250	33.8	14.9
13605254	55.7	17.3
13605255	38.9	3.3
13605258	45.8	19.6
13605259	74.5	2.2
13605260	30.7	30.2
13605261	51.0	9.7
13605262	45.7	1
13605264	44.9	17.8
13605266	20.7	9
13605267	38.6	4.8
13605268	29.5	14.9
13605270	29.9	1
13605271	56.0	4.7
13605276	62.7	21.7
13605277	1.8	12.4
13605280	19.4	1.1
13605282	53.5	47.1
13605283	31.8	26.1
13605285	63.1	9.9
13605286	68.5	5.2
13605289	68.4	17.3
13605290	47.5	5.4
13605295	8.8	23.5
13605299	11.3	2.5
13605300	50.0	5.2
13605302	42.1	10.3
13605303	16.7	24.6
13605305	12.2	2.5
13605307	21.2	77.1
13605308	65.5	1
13605312	18.1	8.7
13605315	32.9	7.7
13605316	32.4	3.2
13605317	39.2	7.4
13605318	26.4	17.9
13605324	28.3	5.2
13605327	52.2	53.2
13605329	54.7	26.7
13605330	26.8	13.7
13605331	5.4	3.8

13605332	19.9	1.6
13605333	37.2	1.5
13605334	59.3	6.7
13605339	69.7	1
13605340	43.0	1
13605341	49.9	24
13605342	25.6	15.4
13605347	57.6	5.9
13605348	49.9	138.6
13605349	55.9	13.6
13605350	76.7	2.1
13605351	46.9	14
13605354	3.6	1
13605355	28.5	4.9
13605356	43.3	3.9
13605357	26.5	2.1
13605361	60.3	1.5
13605362	41.5	34.2
13605363	20.1	26.8
13605364	36.0	130.2
13605365	51.8	34.7
13605366	40.9	39.2
13605367	58.0	13.5
13605369	69.9	12.9
13605370	0.2	1.5
13605372	44.0	169.8
13605374	37.7	3.7
13605377	37.5	10.8
13605379	41.7	8.9
13605380	66.0	3.9
13605382	62.8	11.9
13605388	67.6	1.4
13605390	67.8	205.1
13605397	45.5	9.5
13605399	48.6	3.2
13605400	16.0	5.8
13605401	13.0	2.8
13605402	14.1	19
13605407	64.0	13.3
13605409	74.1	2.2
13605411	16.5	33.2
13605413	49.1	6.9
13605414	22.6	3.4
13605415	54.2	22.5
13605417	42.5	13.7
13605418	63.4	4.5
13605420	56.4	3.4
13605421	53.3	18
13605422	55.4	13.5
13605423	57.9	38.7
13605426	40.2	2
13605428	17.4	10.2
13605431	37.0	324.9
13605433	36.9	157.1
13605435	38.7	76.3
13605438	20.5	5
13605440	33.4	83.9

13605441	32.1	46.6
13605443	55.7	16.7
13605445	41.7	33.5
13605447	0.9	4.1
13605451	38.4	1
13605452	34.0	3.1
13605454	33.0	13.9
13605455	75.0	35.3
13605456	47.6	8.7
13605458	36.0	1
13605464	2.0	3.3
13605466	47.9	26.2
13605467	64.6	2.8
13605468	45.9	2.9
13605469	1.1	46.2
13605470	79.4	5.1
13605471	39.6	31.3
13605472	65.1	1.5
13605481	77.8	7.1
13605485	68.4	38
13605487	4.6	1.4
13605488	6.7	1
13605490	16.8	1
13605496	58.3	24.5
13605499	4.6	1.9
13605500	19.4	531.1
13605501	28.7	2.4
13605503	7.8	9.9
13605504	10.5	11.1
13605506	44.9	1.9
13605508	5.2	2
13605512	35.0	1574.5
13605515	33.5	13
13605522	49.1	42.8
13605523	55.0	3.8
13605524	20.5	7.6
13605525	1.7	454.7
13605526	30.8	22.4
13605527	37.9	2.6
13605528	20.7	4.4
13605529	52.1	16.9
13605531	50.1	3.6
13605532	31.1	23.4
13605535	39.3	10.6
13605538	20.9	10.8
13605541	65.8	7.3
13605543	6.9	1.4
13605545	76.5	36.6
13605546	0.5	147.9
13605547	68.2	61.1
13605549	50.1	42.3
13605550	71.8	37.6
13605552	60.9	75.7
13605555	68.5	1.3
13605556	72.7	54.6
13605557	65.0	4.1
13605560	76.5	33.9

13605562	32.3	20.3
13605565	72.9	37.7
13605566	69.0	18
13605567	77.7	1.1
13605570	4.6	1
13605571	8.3	33.3
13605575	13.1	1
13605576	7.6	31
13605579	61.2	9.1
13605580	14.7	56.9
13605581	63.9	36.3
13605582	15.9	7.6
13605584	16.9	12.6
13605585	60.2	3.7
13605586	65.7	1
13605587	63.6	2.7
13605591	21.7	3
13605592	26.6	3.9
13605594	44.1	42.1
13605600	58.3	8.7
13605601	36.5	4.3
13605603	52.9	35.1
13605604	22.2	1
13605605	25.8	10
13605606	49.9	8.9
13605610	48.1	59.5
13605613	3.8	2.9
13605614	24.9	9.2
13605618	21.9	12.9
13605619	39.4	63.1
13605621	37.9	42
13605622	27.1	33.2
13605624	34.4	1.7
13605625	55.7	2.3
13605626	42.0	199.9
13605629	37.4	48.7
13605633	11.2	1
13605637	0.6	13.5
13605638	32.3	59.9
13605639	10.0	20.8
13605641	54.3	40.3
13605643	59.3	15.7
13605647	32.1	21
13605649	11.3	1
13605650	7.3	9.9
13605651	79.5	134.5
13605661	47.0	8.7
13605664	25.5	16.4
13605666	60.0	1.3
13605667	33.6	2.2
13605668	35.7	18.7
13605669	47.1	6.1
13605670	32.8	6
13605671	32.9	8.5
13605672	77.1	6.5
13605673	30.2	1.4
13605674	52.1	12

13605678	17.2	94.2
13605679	59.3	14.6
13605680	24.3	4.6
13605682	45.2	15.3
13605685	38.4	16.3
13605686	20.4	6
13605687	30.9	1
13605688	20.0	4
13605690	41.8	63.1
13605691	24.2	7.2
13605692	36.7	32.3
13605693	28.6	7.2
13605697	0.6	16.3
13605700	3.2	1
13605701	0.4	77.2
13605702	9.3	237.6
13605706	26.0	1
13605709	44.2	114.4
13605711	44.7	37
13605717	21.4	3.2
13605720	69.0	4.5
13605723	57.1	44.4
13605726	74.3	1.4
13605728	62.8	6.3
13605729	0.8	11.1
13605738	75.4	12.6
13605739	35.4	4.2
13605740	60.1	22.8
13605741	64.6	3.3
13605745	43.3	14.3
13605752	33.0	32.9
13605757	0.1	5.1
13605758	22.9	19
13605759	1.9	55.7
13605762	24.3	3.7
13605765	32.5	14.2
13605767	31.1	14.6
13605776	56.6	5.7
13605777	0.9	12.4
13605780	56.1	35.2
13605783	54.7	7.3
13605784	55.9	1.7
13605787	4.4	3
13605789	30.5	10.1
13605790	36.8	39.2
13605792	10.9	1
13605797	31.7	1
13605798	35.6	30.7
13605800	21.0	1
13605805	52.7	1.2
13605816	10.0	115.1
13605828	59.8	4.1
13605829	43.1	26.7
13605830	53.9	1
13605832	67.6	6
13605834	32.4	17.7
13605837	23.6	2.7

13605841	62.2	2.1
13605842	39.7	69.8
13605844	49.1	11.5
13605849	40.1	27.6
13605850	10.8	31.4
13605855	3.7	10.1
13605878	61.3	2.6
13605881	67.1	7.8
13605882	62.1	15.4
13605884	60.6	94.9
13605891	67.0	1
13605896	24.7	1
13605898	48.2	1.5
13605901	52.6	1.6
13605903	54.1	253.6
13605904	51.6	24.3
13605907	49.5	12.2
13605908	47.9	56.3
13605911	32.0	7.5
13605916	32.5	25.8
13605920	18.5	2
13605928	44.3	163.8
13605932	56.8	1
13605946	1.9	92.2
13605947	46.8	10
13605953	36.5	70.1
13605954	1.1	272.6
13605960	46.6	9.3
13605972	10.8	5
13605976	10.4	6.7
13605980	12.5	2.2
13605986	13.0	2.3
13605993	66.4	3.7
13606002	39.2	4.4
13606006	37.3	4.5
13606007	30.4	11.7
13606009	17.9	1.4
13606010	49.6	3.9
13606013	32.3	42.9
13606016	35.7	7.2
13606017	45.8	32.9
13606021	37.5	23.3
13606023	21.5	1
13606025	69.4	46.6
13606026	0.9	7.2
13606028	68.3	35.2
13606029	60.3	15.5
13606031	79.6	7.1
13606033	24.1	6.3
13606035	40.5	5.9
13606036	17.2	2.8
13606038	2.5	9.6
13606039	31.6	4.3
13606043	61.6	4.1
13606045	19.6	1.2
13606047	4.0	2.6
13606048	64.2	159.9

13606050	70.9	40
13606052	63.3	42.6
13606053	66.2	49.5
13606055	4.4	11.9
13606056	16.6	85.7
13606058	59.5	55
13606059	67.5	17.1
13606060	2.1	8.7
13606062	30.2	1.7
13606063	36.7	11.7
13606065	18.2	11
13606069	68.1	18.8
13606070	59.6	10.4
13606074	52.5	24.1
13606075	2.9	1.1
13606076	3.8	14.4
13606077	59.3	21.5
13606078	0.5	46.6
13606080	39.9	13.7
13606081	47.9	5.4
13606083	52.0	7.6
13606085	52.9	26.4
13606088	23.4	24.4
13606089	57.2	31.1
13606090	17.0	5.9
13606091	35.8	2.7
13606093	45.8	31.2
13606094	54.4	5.3
13606095	56.8	145.9
13606096	19.9	2.4
13606097	45.2	5.1
13606098	36.2	61.4
13606099	35.0	3
13606100	20.1	7.9
13606102	31.7	22.2
13606106	38.5	17.8
13606107	55.9	42.2
13606108	45.3	15.4
13606109	21.0	2.7
13606111	37.2	161.6
13606114	53.5	1
13606115	36.2	7.6
13606121	30.0	17.6
13606122	21.5	9.9
13606123	51.4	3.4
13606128	8.5	1
13606129	60.1	1
13606130	11.5	2.4
13606131	0.2	1
13606132	30.7	7
13606133	0.2	2.6
13606135	8.7	2.5
13606140	9.9	19.7
13606145	66.5	12.7
13606147	43.2	93.1
13606149	43.3	20.4
13606150	4.2	17.6

13606151	34.9	34.4
13606153	63.3	9.2
13606155	14.7	1
13606159	30.6	28.4
13606160	20.2	13.5
13606161	0.6	29.7
13606162	38.7	3.8
13606163	54.1	1.1
13606165	26.6	1
13606166	51.9	4.5
13606167	48.7	33.5
13606168	63.9	1
13606173	32.2	8.6
13606175	23.0	45.3
13606176	30.3	37.8
13606177	23.8	1
13606179	37.2	6
13606180	55.8	98.6
13606181	17.6	2
13606183	53.6	3.5
13606184	37.1	8.8
13606185	57.4	10.4
13606187	44.6	2.4
13606188	77.0	36.1
13606191	39.3	19.2
13606197	34.8	3
13606198	28.9	12.5
13606199	21.8	17.4
13606200	0.5	44.6
13606202	25.5	1
13606203	22.9	1
13606208	3.4	1
13606209	21.1	1
13606215	3.8	1
13606216	60.7	32.4
13606221	33.0	7
13606224	58.0	6.4
13606229	0.4	3.5
13606230	34.1	111
13606231	7.8	5.2
13606233	17.6	3.6
13606236	38.0	11.2
13606237	10.6	3
13606238	10.1	4.8
13606239	31.1	35.7
13606241	34.6	1.2
13606243	60.5	11
13606244	20.6	119.6
13606246	37.9	13
13606251	0.8	9.8
13606252	70.3	1
13606253	54.2	2.5
13606254	73.1	37.4
13606255	56.1	5.6
13606257	4.7	1.2
13606260	24.5	1
13606264	17.5	23.5

13606266	64.2	8.5
13606269	52.9	9.1
13606273	28.6	4
13606276	52.9	18.8
13606277	32.2	2.5
13606278	37.1	6.8
13606280	49.2	4.3
13606281	0.7	15.5
13606284	60.6	18.6
13606288	9.8	21.9
13606289	49.3	1
13606290	30.5	8.1
13606291	38.3	6.3
13606294	31.3	12
13606296	51.8	15.9
13606297	29.9	38.3
13606303	27.6	6.9
13606304	44.8	6.4
13606305	77.6	26
13606307	27.7	29.9
13606308	2.8	2.2
13606309	41.4	1.8
13606310	29.3	5.1
13606312	65.7	32.8
13606313	7.6	6.3
13606314	42.3	14.6
13606319	30.4	14.6
13606323	39.3	1
13606324	71.6	4.2
13606325	65.0	44.1
13606326	0.2	1.8
13606327	8.4	106.1
13606328	7.1	8.6
13606331	16.8	9.8
13606332	70.3	24.8
13606338	0.3	2.3
13606340	0.7	72
13606341	63.2	2.9
13606342	36.5	12
13606343	4.4	1
13606345	78.0	94.7
13606346	8.6	3.1
13606347	46.4	8.7
13606349	0.5	64.2
13606357	3.2	3.2
13606365	49.7	18
13606366	16.2	304.8
13606367	31.8	4.3
13606368	23.3	6.6
13606371	36.2	4.1
13606373	53.2	15.7
13606377	34.6	1
13606379	26.3	35.3
13606381	6.6	10.3
13606383	19.4	20.5
13606386	65.8	282.4
13606387	39.6	14.2

13606389	37.7	6.3
13606390	0.4	41
13606391	20.9	1.6
13606394	51.4	1.4
13606397	51.9	8.2
13606398	59.6	97
13606399	61.2	3
13606400	28.5	45.9
13606403	34.1	16.8
13606407	72.4	43.7
13606408	24.4	30.9
13606410	72.1	1.3
13606412	3.5	1
13606414	76.6	1.5
13606415	78.5	1.5
13606417	78.2	7.2
13606419	49.9	3.3
13606423	3.3	8.8
13606425	4.9	5.1
13606426	63.0	15.4
13606428	5.7	6.2
13606429	46.5	3.1
13606430	58.9	8.9
13606431	36.0	2.9
13606433	0.4	18.2
13606438	52.7	1
13606439	53.2	1
13606440	0.9	4.1
13606441	28.2	19.8
13606443	37.4	4.2
13606445	51.9	2.1
13606446	34.7	18.1
13606447	17.4	53.4
13606448	29.5	1
13606451	15.1	5
13606452	43.9	43.4
13606454	26.4	17.7
13606455	43.0	3.5
13606458	30.5	9.1
13606460	27.6	1.6
13606462	55.8	12.2
13606463	56.9	2.5
13606465	57.6	17.8
13606466	51.6	24.1
13606468	32.4	35.1
13606471	19.5	1.9
13606472	24.5	9.4
13606474	22.9	86.3
13606475	52.9	10
13606476	27.6	1.4
13606479	6.8	21
13606480	51.6	11.5
13606484	44.8	8.7
13606486	30.7	7.6
13606487	14.5	3.3
13606490	66.3	46.5
13606507	37.5	41

13606508	50.2	6.3
13606510	5.1	11.8
13606511	39.6	16.2
13606512	68.8	2.3
13606514	43.7	14.6
13606515	3.1	1
13606517	14.0	1.8
13606520	39.1	17.5
13606521	72.1	11.8
13606522	25.1	39.4
13606523	22.1	3.9
13606524	0.6	42.7
13606525	3.5	1.6
13606526	62.7	5.6
13606527	0.7	6.2
13606528	2.2	18.4
13606529	36.3	1
13606530	60.2	47.7
13606533	0.7	21
13606535	61.3	112.1
13606537	4.2	2.8
13606538	9.9	36.5
13606541	9.6	11.5
13606543	10.4	2.8
13606545	10.7	42.5
13606550	69.5	49.6
13606551	75.0	6.9
13606553	20.7	23.8
13606554	29.2	18.1
13606555	10.7	112.5
13606558	52.6	295.2
13606560	41.3	3.2
13606561	25.7	15
13606564	47.5	7.5
13606565	18.2	1.1
13606568	61.4	3.4
13606569	35.9	9.5
13606570	36.2	25.8
13606572	3.4	1
13606573	36.8	120.3
13606574	37.4	4.7
13606576	17.5	8.4
13606577	27.9	73.3
13606578	50.2	7.3
13606580	0.9	7.3
13606581	21.9	12
13606583	41.7	2.3
13606585	31.2	4.4
13606589	43.7	6.2
13606593	60.0	17.6
13606595	73.2	182.4
13606601	65.9	168.4
13606602	77.2	1.3
13606604	0.3	22.3
13606606	63.2	1
13606609	15.7	4.7
13606610	61.0	1

13606615	13.6	7.8
13606617	14.0	3.7
13606618	13.0	1
13606620	45.1	22.6
13606623	7.8	5.3
13606624	34.2	17.7
13606625	20.5	176.8
13606626	37.2	33.5
13606627	55.7	33
13606629	22.2	2.2
13606630	20.9	14.4
13606632	51.7	34.6
13606633	63.1	3.3
13606635	15.3	14.3
13606637	33.0	12.3
13606638	30.1	2.3
13606639	55.4	1.3
13606640	31.5	2.6
13606641	68.4	12
13606642	10.8	1
13606645	59.5	32.1
13606646	73.8	4.2
13606651	3.2	4.1
13606654	53.6	342.8
13606655	1.0	16.6
13606656	31.3	24.6
13606657	42.8	10.6
13606659	43.0	9.3
13606662	9.4	2.4
13606667	20.6	41.5
13606668	32.8	96
13606670	26.0	6.2
13606671	33.8	12.1
13606672	13.0	73.8
13606674	37.9	1
13606676	4.5	5.3
13606677	12.4	31.7
13606679	49.1	49.9
13606680	31.7	1
13606681	22.0	28
13606682	24.6	24
13606685	44.0	17.8
13606687	71.3	36.1
13606691	30.0	4.8
13606692	10.4	6.7
13606693	34.4	35.4
13606694	14.9	6.3
13606695	24.4	3.1
13606697	59.6	1.1
13606698	4.1	16.2
13606701	70.1	243.7
13606702	2.4	39.4
13606703	49.3	15.3
13606705	33.5	11.9
13606707	0.2	1.6
13606709	79.9	35.1
13606711	43.9	63.5

13606712	63.4	19.4
13606716	60.8	11.6
13606720	60.8	7.6
13606721	13.5	242.9
13606722	15.5	1
13606723	30.3	1.9
13606724	14.1	2736.5
13606727	2.9	9.4
13606728	15.7	1
13606729	77.6	87.1
13606730	68.9	4.9
13606734	55.1	27.5
13606736	56.1	13.7
13606739	2.5	8
13606740	0.9	8.9
13606743	61.0	10.1
13606745	60.4	10.2
13606746	31.4	4.2
13606750	38.3	9.9
13606752	32.7	6.2
13606753	46.1	11
13606754	68.2	7.1
13606755	7.2	464.1
13606757	16.2	31.6
13606758	55.7	15.9
13606759	39.0	4
13606760	31.3	1.8
13606762	29.3	18.7
13606764	75.3	16.8
13606765	41.2	1
13606767	18.2	192.2
13606768	35.4	10.7
13606770	10.6	134.8
13606771	35.3	3.9
13606776	25.6	22.1
13606777	43.5	5.4
13606778	17.0	53.2
13606779	72.0	1
13606780	7.6	13.9
13606782	12.6	11.2
13606783	2.8	2.7
13606785	71.7	19.9
13606786	36.2	4.6
13606789	0.3	1.4
13606790	8.0	5.3
13606793	76.8	1.8
13606796	6.6	8.4
13606798	71.3	24.2
13606799	0.8	29
13606801	9.2	5.8
13606803	4.7	146.5
13606804	2.0	18.9
13606806	15.5	8.5
13606811	49.0	56.1
13606815	18.4	12.3
13606816	61.0	6.3
13606817	0.1	20.2

13606819	30.4	48.9
13606820	8.6	10.7
13606821	49.4	24.7
13606822	35.2	1
13606824	70.0	11.7
13606825	37.9	15.4
13606827	43.9	24.5
13606830	34.5	3.6
13606831	50.6	2.8
13606833	24.5	22.4
13606834	58.9	4.7
13606836	40.5	71.7
13606839	29.0	31.4
13606840	25.1	43.8
13606841	31.9	10.1
13606842	43.1	44.3
13606843	26.3	9.5
13606846	27.1	3.9
13606848	35.4	19.8
13606849	37.5	1
13606852	0.8	7.7
13606853	51.7	29.3
13606854	62.9	11
13606856	0.6	38.3
13606857	40.6	13.1
13606858	31.6	72.2
13606859	24.3	19.8
13606861	71.6	5.7
13606864	4.5	6.9
13606867	9.7	1
13606869	69.1	7.7
13606870	0.5	27.8
13606872	54.9	73.9
13606877	62.2	3.2
13606879	34.1	24
13606880	2.6	16.4
13606885	36.5	115
13606890	60.4	95.1
13606891	67.4	311
13606895	78.5	4.4
13606896	4.7	7.4
13606900	53.7	34.4
13606902	0.6	32.3
13606904	29.7	11.4
13606907	3.9	713.1
13606908	13.6	15.7
13606909	48.1	24.9
13606912	2.5	24.2
13606913	0.6	32.1
13606917	55.0	22.7
13606920	4.4	3.9
13606921	18.8	130.4
13606923	20.3	211.5
13606926	31.2	140.3
13606927	31.6	158.4
13606928	22.8	1
13606934	30.1	103.8

13606937	26.3	4.4
13606939	35.4	18.3
13606942	0.2	9.8
13606943	8.7	13
13606945	30.8	4.5
13606947	13.2	74
13606948	21.3	51.4
13606949	11.5	1
13606950	7.0	15.9
13606951	0.2	5.1
13606952	27.6	1
13606953	49.2	29.4
13606956	11.7	2.4
13606957	59.3	70.1
13606958	71.1	2.7
13606959	52.0	17.5
13606961	64.3	38.4
13606963	20.6	15
13606965	23.6	9.2
13606967	68.4	45.6
13606970	51.6	18.9
13606971	0.6	102.8
13606974	0.8	37.4
13606975	12.6	1
13606980	61.9	5.8
13606981	20.0	17.9
13606982	60.8	155.1
13606984	3.1	1
13606986	4.2	18.8
13606987	50.5	44.9
13606990	19.5	1
13606992	52.0	295.7
13606994	39.4	1.6
13606995	27.8	8.1
13606997	0.8	6.4
13606998	0.5	43.2
13606999	15.0	6.3
13607002	49.4	9.5
13607111	28.2	6.9
13607112	61.2	4.4
13607113	9.4	1
13607114	43.1	5.8
13607115	21.5	11.8
13607116	35.9	119.3
13607117	34.3	11.1
13607118	3.5	1
13607120	19.2	1
13607121	24.9	82.3
13607122	3.8	1
13607127	0.6	23.2
13607128	35.7	1
13607129	43.5	20.7
13607131	40.3	1.9
13607132	31.9	2.3
13607133	36.3	1.5
13607134	37.5	13.4
13607135	39.6	20.5

13607296	47.1	30.6
13607299	0.2	1.9
13607302	20.0	16.6
13607310	2.5	12.7
13607313	32.7	1
13607314	31.5	1.7
13607316	11.6	2.9
13607318	10.0	19.2
13607320	0.4	74
13607321	21.2	1.1
13607323	65.5	14.8
13607325	9.5	1.3
13607326	46.5	10.5
13607330	74.6	5.9
13607333	0.6	29.4
13607334	0.7	26.5
13607336	4.8	6.3
13607337	4.7	1
13607340	9.8	7.4
13607342	10.6	16.8
13607343	0.7	5.2
13607346	74.0	24.6
13607349	15.0	1.3
13607350	7.6	8.6
13607353	1.0	59.4
13607354	13.6	2.4
13607356	17.1	36.7
13607358	42.5	67.3
13607359	8.0	3.1
13607360	65.2	10.5
13607361	49.0	10.9
13607363	22.8	12.5
13607364	13.3	1
13607365	63.8	18.3
13607366	53.4	4.9
13607368	54.5	5.6
13607371	47.5	17.8
13607372	23.2	1.6
13607373	2.9	1.3
13607375	44.0	34.6
13607377	17.6	80.3
13607380	16.7	1
13607385	19.6	5.6
13607420	24.8	8.3
13607421	55.3	57.1
13607423	0.3	3.9
13607426	49.5	7.8
13607427	2.3	3.9
13607430	48.8	4.4
13607431	2.7	6.5
13607432	43.5	2.4
13607433	40.9	10.5
13607435	60.0	38.4
13607436	0.5	42.9
13607437	8.8	1
13607438	48.3	35.9
13607439	34.7	1

13607440	4.0	11.3
13607441	75.2	1
13607442	65.0	55.1
13607443	4.5	15.2
13607445	71.1	3.2
13607446	7.8	2
13607447	70.4	9.6
13607448	53.9	1
13607451	45.6	7.8
13607452	29.4	10.6
13607453	0.8	9.4
13607456	19.3	3.4
13607457	14.8	18.3
13607459	24.1	2.9
13607460	12.4	21.3
13607461	45.8	5.1
13607462	29.3	40.9
13607463	3.6	1
13607464	58.2	86.4
13607466	42.3	31.4
13607467	39.7	121.3
13607468	4.0	9.1
13607471	39.5	13.3
13607472	31.2	20.2
13607473	33.9	87.6
13607474	18.8	1.8
13607475	0.6	18.9
13607476	20.8	1
13607477	0.6	45.9
13607478	53.2	7.1
13607479	26.0	1
13607481	28.1	18.7
13607483	22.0	1.4
13607484	17.8	107.2
13607485	43.1	129
13607488	37.1	1.8
13607489	30.5	7
13607703	29.8	212.1
13607704	35.2	27.7
13607705	77.9	10.9
13607708	18.5	1.3
13607709	65.9	4.9
13607710	60.8	49.6
13607713	0.8	22.6
13607714	0.7	55.5
13607715	34.2	9
13607716	48.1	65.9
13607718	72.3	39.6
13607719	73.3	14.3
13607720	11.9	113.1
13607811	68.9	9.7
13607814	74.7	10.1
13607816	77.6	4.8
13607817	51.6	5.4
13607818	0.9	7.4
13607819	1.7	39.2
13607821	38.6	21

13607822	51.1	2.2
13607825	35.4	4.2
13607827	43.8	8.7
13607828	39.9	28.1
13607831	0.3	10.6
13607832	78.3	4.6
13607839	71.3	11.2
13607842	29.5	22.2
13607844	4.3	2
13607848	0.8	2.6
13607849	78.9	11.1
13607850	1.6	6.5
13607852	63.5	1.1
13607854	77.7	77.8
13607855	48.6	22.8
13607857	16.9	38
13607861	14.8	1.4
13607863	10.4	36
13607865	16.9	3.6
13607866	12.7	115.7
13607871	65.1	48
13607874	25.5	12.1
13607877	16.3	11.3
13607879	77.0	36.8
13607880	25.4	3.1
13607885	60.0	15.9
13607887	37.9	15.1
13607888	51.7	5.7
13607889	36.3	12.5
13607891	55.9	8.5
13607892	27.7	1
13607897	12.1	7
13607899	55.2	26.9
13607901	0.7	53.6
13607909	45.0	2.8
13607915	5.3	1.7
13607917	48.4	9.1
13607918	29.1	14.8
13607920	22.7	1
13607922	7.2	1.7
13607927	1.2	41.2
13607931	47.6	32.4
13607934	3.0	3.1
13607938	36.5	1.2
13607939	31.0	22.2
13607940	32.4	8.9
13607945	62.4	10.9
13607946	0.3	1.1
13607949	76.5	111.2
13607953	30.3	3.8
13607954	27.3	16.3
13607960	2.9	1
13607961	21.2	2.2
13607962	65.8	13.2
13607966	7.2	8.3
13607968	38.1	62.2
13607970	61.7	24.4

13607972	41.6	5.1
13607983	54.9	6.8
13607984	1.5	24.8
13607987	53.2	114.9
13607988	2.3	4.8
13607989	0.3	34.3
13607993	23.3	2.9
13607996	48.1	4.9
13607999	50.2	6.1
13608000	21.1	38.5
13608002	42.9	1.6
13608004	26.7	24.8
13608005	32.2	5.7
13608007	29.6	12
13608008	41.2	18.9
13608011	37.6	5.2
13608012	31.6	112.3
13608013	53.4	13.1
13608014	5.2	176.8
13608015	49.8	6
13608016	43.7	139.8
13608017	22.4	3.9
13608019	0.4	88.5
13608022	0.4	59.8
13608024	35.0	16.3
13608026	29.3	22.9
13608070	65.1	22.3
13608078	2.8	1
13608079	26.3	1.9
13608080	24.3	10.6
13608083	9.4	134.2
13608088	44.6	140.8
13608089	36.5	13.2
13608090	23.5	1
13608091	47.0	311.7
13608093	4.0	9.2
13608095	38.8	1.2
13608096	52.8	1
13608100	54.7	22.3
13608101	37.2	16.5
13608107	35.8	9.2
13608108	29.1	11.7
13608109	31.0	2.5
13608111	35.0	4.1
